# The *Plasmodium falciparum* transcriptome in severe malaria reveals altered expression of genes involved in important processes including surface antigen–encoding *var* genes

**DOI:** 10.1371/journal.pbio.2004328

**Published:** 2018-03-12

**Authors:** Gerry Q. Tonkin-Hill, Leily Trianty, Rintis Noviyanti, Hanh H. T. Nguyen, Boni F. Sebayang, Daniel A. Lampah, Jutta Marfurt, Simon A. Cobbold, Janavi S. Rambhatla, Malcolm J. McConville, Stephen J. Rogerson, Graham V. Brown, Karen P. Day, Ric N. Price, Nicholas M. Anstey, Anthony T. Papenfuss, Michael F. Duffy

**Affiliations:** 1 Bioinformatics Division, Walter and Eliza Hall Institute of Medical Research, Parkville, Victoria, Australia; 2 Department of Mathematics and Statistics, University of Melbourne, Victoria, Australia; 3 The Eijkman Institute for Molecular Biology, Jakarta, Indonesia; 4 School of Biosciences, Bio21 Institute, University of Melbourne, Melbourne, Victoria, Australia; 5 Department of Medicine and Radiology, Royal Melbourne Hospital, University of Melbourne, Melbourne, Victoria, Australia; 6 Timika Malaria Research Program, Papuan Health and Community Development Foundation, Timika, Papua, Indonesia; 7 Global and Tropical Health Division, Menzies School of Health Research, Charles Darwin University, Darwin, NT, Australia; 8 Department of Biochemistry and Molecular Biology, Bio21 Institute, University of Melbourne, Melbourne, Victoria, Australia; 9 Peter Doherty Institute for Infection and Immunity, Melbourne, Victoria, Australia; 10 The Nossal Institute for Global Health, University of Melbourne, Parkville, Victoria, Australia; 11 Centre for Tropical Medicine and Global Health, Nuffield Department of Clinical Medicine, University of Oxford, Oxford, United Kingdom; 12 Peter MacCallum Cancer Centre, Victorian Comprehensive Cancer Centre, Melbourne, Australia; 13 Department of Medical Biology, University of Melbourne, Parkville, Victoria, Australia; 14 Sir Peter MacCallum Department of Oncology, University of Melbourne, Parkville, Victoria, Australia; Stanford University, United States of America

## Abstract

Within the human host, the malaria parasite *Plasmodium falciparum* is exposed to multiple selection pressures. The host environment changes dramatically in severe malaria, but the extent to which the parasite responds to—or is selected by—this environment remains unclear. From previous studies, the parasites that cause severe malaria appear to increase expression of a restricted but poorly defined subset of the PfEMP1 variant, surface antigens. PfEMP1s are major targets of protective immunity. Here, we used RNA sequencing (RNAseq) to analyse gene expression in 44 parasite isolates that caused severe and uncomplicated malaria in Papuan patients. The transcriptomes of 19 parasite isolates associated with severe malaria indicated that these parasites had decreased glycolysis without activation of compensatory pathways; altered chromatin structure and probably transcriptional regulation through decreased histone methylation; reduced surface expression of PfEMP1; and down-regulated expression of multiple chaperone proteins. Our RNAseq also identified novel associations between disease severity and PfEMP1 transcripts, domains, and smaller sequence segments and also confirmed all previously reported associations between expressed PfEMP1 sequences and severe disease. These findings will inform efforts to identify vaccine targets for severe malaria and also indicate how parasites adapt to—or are selected by—the host environment in severe malaria.

## Author summary

Infection by *Plasmodium falciparum*—the parasite responsible for malaria in humans—can result in a severe disease that can be fatal or in an uncomplicated disease that can be resolved by the host immune system. However, whether the parasites causing severe disease differ from those causing uncomplicated disease is unknown. Several strands of evidence have suggested that parasites causing severe disease may express a restricted set of the *Plasmodium falciparum* Erythrocyte Membrane Protein 1 (PfEMP1) proteins. PfEMP1 proteins are expressed on the surface of the infected red blood cells and elicit protective immunity. We compared the transcriptomes of parasites causing severe and uncomplicated malaria to determine whether these parasites differed in the genes they expressed. We found that the parasites causing severe malaria had altered expression of genes involved in basic metabolism, nuclear processes, and surface expression of PfEMP1. The parasites causing severe malaria had up-regulated expression of a set of PfEMP1 proteins. Some of these PfEMP1s had been previously implicated in severe malaria, lending support to our data. Multiple associations identified between severe malaria and expressed PfEMP1 sequences were novel. These novel, severe disease–associated PfEMP1 sequences could be useful for informing design of vaccines targeting severe malaria disease.

## Introduction

*P*. *falciparum* is the leading cause of fatal malaria and is responsible for the death of over 400,000 people annually, primarily in sub-Saharan Africa [1]. However, severe disease also occurs in Southeast Asia, and Papua is the Indonesian province with the highest prevalence of malaria [1]. Severe malaria due to *P*. *falciparum* can manifest as multiple, diverse clinical syndromes [2], but a critical common feature is the sequestration of erythrocytes infected with mature parasites in the microvasculature (reviewed in [3]).

Comparative genome-wide analyses of parasite isolates that cause severe and uncomplicated malaria can be used to identify genes associated with parasite virulence and pathology. This knowledge could inform therapy and the design of vaccines targeting severe disease. Although previous microarray studies of ring-stage–and/or ex vivo–cultivated mature parasites [4,5] showed no differences between parasites causing severe and uncomplicated malaria, transcriptomic differences indicative of fundamental metabolic variations between clinical isolates have been reported. These differences were apparent in clinical isolates segregated by transcriptional profile alone [6] or by direct [7] or surrogate measures of parasitemia [8]. Limitations of these studies included the need to cultivate the isolates prior to analysis, the absence of clinical severity phenotype [8], and the inability to directly compare severe and uncomplicated malaria in the same study population [7]. In the current study, we used massively parallel sequencing technology to undertake comparative analysis of transcriptomes from parasites associated with uncomplicated and severe malaria in the same population. We found a unique parasite transcriptional profile that was associated with severe malaria. While elements of this profile were congruent with reported profiles of clinical isolates [6,8], these have not been previously linked with severe malaria phenotype. Genes deregulated in severe malaria were involved in pathways including central carbon metabolism, folate biosynthesis, histone methylation, chaperone function, and surface expression of *P*. *falciparum* Erythrocyte Membrane Protein 1 (PfEMP1).

PfEMP1 is the immunodominant, variant surface antigen of *P*. *falciparum* [9]. PfEMP1 is expressed on the surface of the infected erythrocyte (IE), where it mediates adhesion to diverse host receptors. PfEMP1 binding to receptors on endothelium leads to the pathogenic sequestration of IEs in the microvasculature (reviewed in [10]). The resulting obstruction is probably exacerbated by IEs binding receptors on uninfected erythrocytes to form ‘rosettes’ [11]. By switching between single, expressed PfEMP1 variants, the parasite can change receptor specificity and also avoid the acquired immune response, leading to chronic and recrudescent infections. A parasite’s genome contains approximately 60 *var* gene copies that code for PfEMP1 [12], and immune pressure has driven evolution of extreme diversity in PfEMP1s such that there is very little overlap in *var* repertoires [13–16].

Even with such large sequence diversity, *var* genes can be classified into three broad groups based on their upstream sequence (UPS; A, B, and C) [12,17]. Group A *var* genes appear to have diverged from groups B and C in their binding properties [18]. Expression of group A and B *var* genes has been associated with clinical malaria in Papua New Guinea [19,20], severe malaria in Africa [21], and cerebral malaria in Africa [22–24]. The PfEMP1 ectodomain contains multiple, semiconserved Duffy binding-like (DBL) domains and cysteine-rich interdomain regions (CIDRs) that mediate adhesion to host receptors [25]. These domains have been classified into major types—DBLɑ, β, γ, δ, ε, ζ, and x and CIDRα, β, γ, and δ [14,26,27]—and into 147 further subtypes, e.g., CIDRα1.1 [14]. Multiple domain cassettes (DCs) containing conserved, sequential arrangements of 2 or more domain subtypes have also been identified [14].

A conserved group of variant surface antigens that are presumably a subset of PfEMP1s appear to be expressed by parasites causing severe disease. These antigens are encountered early in life and are recognised more widely by sera from semi-immune children than antigens expressed by parasites causing uncomplicated disease [28,29]. The expression of a conserved subset of PfEMP1s by parasites that cause severe malaria probably explains why immunity to severe malaria is acquired more rapidly than immunity to uncomplicated malaria [30,31]. The conserved PfEMP1 VAR2CSA is expressed by parasites causing malaria in pregnancy, but associations with entire PfEMP1s have not been detected for other severe malaria disease syndromes.

However, at a finer resolution than whole PfEMP1s, some of the PfEMP1 domains that bind specific host receptors and/or are expressed in severe malaria have been identified. CIDRα1 binds endothelial cell protein C receptor (EPCR) [32], and its expression has been linked to severe malaria in children and adults [33–35], whilst rosetting is associated with severe malaria and expression of the DBLα1-CIDRβ/γ/δ head structure [11,36,37]. DBLβ5 from group B *var* genes and specific motifs in DBLβ1 and DBLβ3 from group A *var* genes bind intercellular adhesion molecule 1 (ICAM1) [38–42], and cerebral malaria has been associated with ICAM1 binding [43,44] and expression of group A carrying tandem CIDRα1-DBLβ1/3 domains [42]. DBLβ12 binds the host receptor gC1qR, and its expression is also associated with severe malaria [45]. Elevated expression of a number of DCs has also been associated with severe malaria; these included DC8 (DBLα2-CIDRα1.1-DBLβ12-DBLγ4/6) [34,45–49], DC13 (DBLα1.7-CIDRα1.4) [32,49], DC4 (DBLα1.4-CIDRα1.6-DBLβ3) [39], DC5 (DBLγ12-DBLδ5-CIDRβ3/4) [48], and DC6 (DBLγ14-DBLζ5-DBLe4) [34].

Due to the immense diversity seen in *var* gene domains, attempts have been made to investigate them by concentrating on more conserved sequence or homology blocks [14,50]. Few studies have attempted to link these conserved blocks with disease severity, although one study found an association between homology blocks 219 and 486 and rosetting, whereas homology block 204 was associated with impaired consciousness [51].

All of the previously reported associations between severe disease and *var* gene expression relied on PCR using primers derived from *var* sequences of laboratory isolates. In contrast, RNA sequencing (RNAseq) of clinical samples can be used to assemble all expressed *var* sequences, regardless of their homology to the *var* genes of sequenced laboratory isolates. In the current study, innovative bioinformatic approaches were used to identify multiple novel associations between severe disease and differential expression of *var* gene sequences at the multi-, single-, and sub-domain levels. Furthermore, we recapitulated all previously described associations between expressed *var* gene sequences and severe malaria. These novel, severe malaria–associated *var* sequences have relevance to efforts to design vaccines targeting severe disease.

## Results

### Malaria patients

Parasites were isolated from the venous blood of 23 patients with severe malaria and 21 patients with uncomplicated malaria (Table 1). Patients with severe malaria tended to be older than those with uncomplicated malaria, but there were no significant differences in *P*. *falciparum* density, haemoglobin (Hb) concentration, or gender. Among patients with severe malaria, 19 had presented with a single diagnostic criterion [2], including 4 with cerebral malaria, 3 with jaundice, 8 with hyperparasitaemia, 3 with prostration, and 1 with acute renal failure. Four patients had 2 or more manifestations of severe malaria: 1 patient with jaundice and acute renal failure, 1 with acute renal failure and acute respiratory distress syndrome, 1 with jaundice and hyperparasitaemia, and 1 with hyperparasitaemia and prostration. The parasite biomass marker Histidine Rich Protein 2 (HRP2) was present at higher concentrations in the plasma of patients with severe malaria than those with uncomplicated malaria (*p* = 0.02). None of the patients had severe malarial anaemia (defined as Hb < 5 g/dL in children <12 years old; Hb < 7 g/dL in adults; Table 1) [2]. These findings suggest that severe *P*. *falciparum* malaria in these patients was associated with sequestration rather than anaemia due to repeat infections [52].

**Table 1:** Patient data.

Variable	Severe malaria median (IQR) *n* = 23	Uncomplicated malaria median (IQR) *n* = 21	*p* (Mann Whitney U test)
Patient age in years	28 (20, 36)	22 (18.5, 24.5)	0.0524
[Hb] g/dL	11.6 (9.6, 13.7)	12.4 (10.6, 14.3)	0.4246
Parasites/ul	39,400 (6,600; 259,378)	30,520 (15,690; 52,800)	0.8726
[Glucose] mg/dL	^§^124 (101.5, 188.3)	nd	
Gender	M 13 F 10	M 9 F 12	0.5647*
HRP2 ng/ul	848 (244, 2398)	391 (114, 541)	0.0213
Number *P*. *fal* mapped reads	5.2e06 (4.8e06, 6.5e06)	6.2e06 (3.9e06, 9.4e06)	0.9

* Fisher’s exact test.

^§^ *n* = 12

Abbreviations: Hb, haemoglobin; HRP2, histidine rich protein 2; IQR, interquartile range; nd, not done; *P*. *fal*, *P*. *falciparum*.

### Preprocessing

RNA quality was assessed using the BioRad Experion system (Fig A in S1 Fig). The median RNA Quality Index (RQI) value was 7.75, and the interquartile range (IQR) was 7.175 to 8.55. Transcriptome libraries were constructed for 44 patient samples (Arrayexpress accession: E-MTAB-5860). Library sizes ranged from 17,054 to 247,859,790 sequence reads (Fig B in S1 Fig). The libraries were aligned to the *Homo sapiens* (GRCh38), *P*. *vivax* (PlasmoDB-11.1 Sal1), and *P*. *falciparum* (PlasmoDB-11.1 3D7) reference genomes, and the proportion of *P*. *falciparum* in the libraries ranged from 0.11% to 88.44% (S1 Table). To identify significant features distinguishing severe and uncomplicated malaria transcriptomes, the transcriptome libraries were subjected to a series of sequence and expression analyses (Fig C in S1 Fig).

### De novo assembly of *var* genes

**Fig 1:**
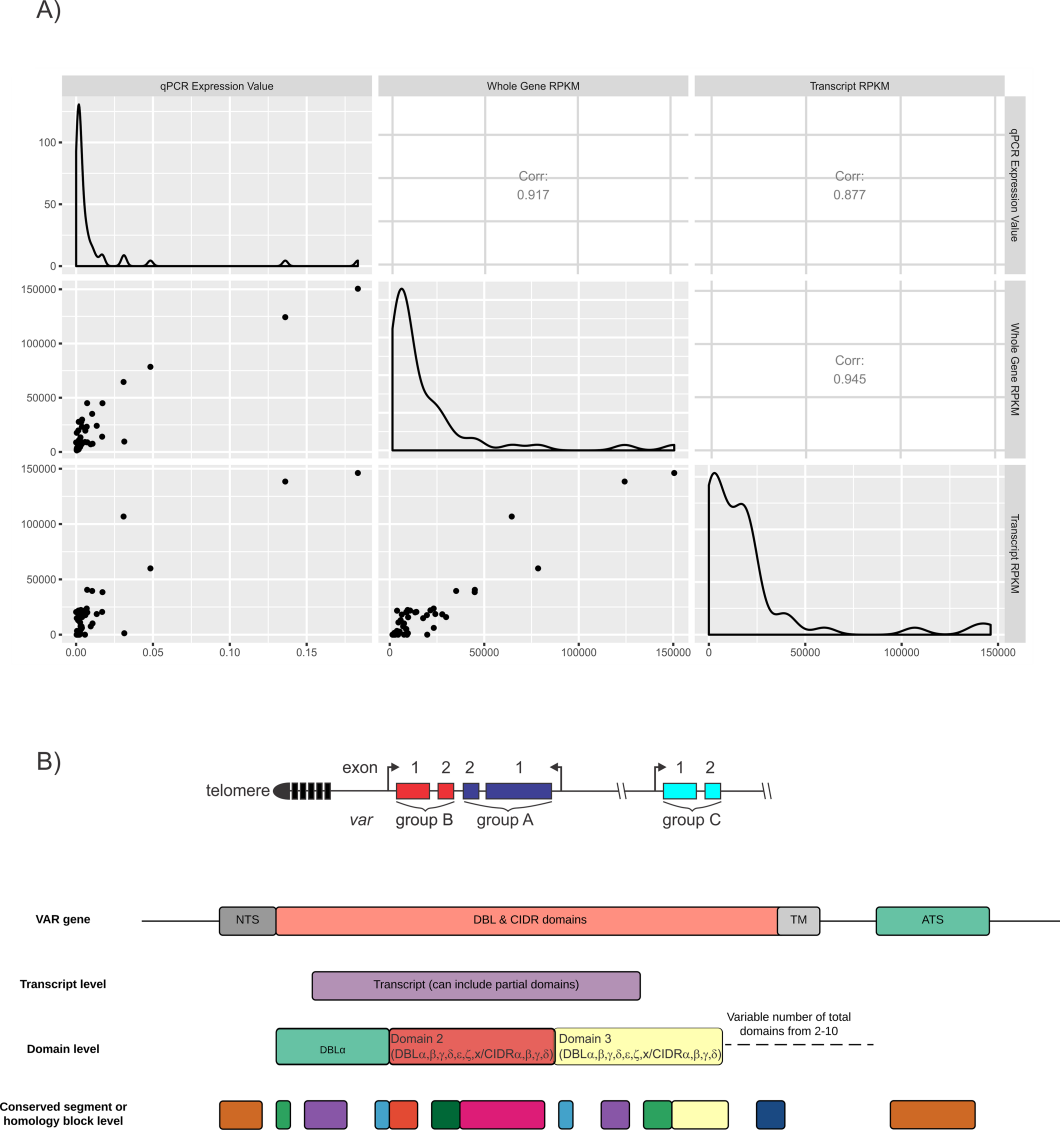
De novo assembly of *var* gene sequence. (A) Expression profiles for the ItG subclone E8B. The assembled transcripts were annotated with their closest BLAST match to the IT4 (a clone of the ItG isolate) sequences from the database of [14]. The expression levels in RPKM are then compared to RPKM levels of reads annotated directly to the whole gene DNA sequences of [14] and to those obtained using qPCR. (B) *Var* gene chromosomal arrangements, group A and B *var* genes are present in subtelomeric clusters, group C *var* genes are present in chromosome internal *var* gene clusters. The different resolutions of *var* sequences investigated in this manuscript are illustrated. *Var* gene transcripts are obtained by de novo assembly of transcriptome data. Domain regions are then identified within these transcripts along with smaller subdomain segments and homology blocks. The number and order of both domains and segments varies between *var* genes. ATS, acidic terminal sequence; BLAST, basic local alignment search tool; CIDR, cysteine-rich interdomain region; DBL, Duffy binding-like; NTS, N-terminal sequence; qPCR, quantitative PCR; RPKM, Reads Per Kilobase of transcript per Million mapped reads; TM, transmembrane.

A pipeline for the de novo assembly of *var* genes from RNAseq data was developed and verified using a *P*. *falciparum* ItG clone (ItG is the parent line of the It4 sequenced clone) for which the *var* repertoire is known. Expression profiles of the assembled transcripts were compared with those obtained by quantitative PCR (qPCR) and were found to correlate significantly (Pearson correlation coefficient R = 0.88) (Fig 1A). The pipeline used the SoapDeNovo-Trans/Cap3 method of [53], which is robust to chimeric assemblies and minimises redundant transcripts. Non-*var P*. *falciparum*, *P*. *vivax*, and *H*. *sapiens* reads were filtered out prior to assembly.

As proof of concept, the pipeline correctly assembled an ItG subclone E8B that expressed predominantly the IT4var04 *var* gene. Additionally, the ItG subclone CS2—with a recombination event between IT4var04 and IT4var08 *var* genes [54]—was correctly assembled (Figs A and B, respectively, in S2 Fig). Alternative approaches were investigated (S2 Table), with the SoapDeNovo-Trans/Cap3 pipeline chosen because it assembled the known samples correctly, was sensitive to low-expressed transcripts, and produced minimal redundancy. The pipeline is available at https://github.com/PapenfussLab/assemble_var.

The assembly pipeline was run separately for each of the 44 patient samples in addition to a pooled sample assembly where all the reads from each patient sample were combined (European Nucleotide Archive [ENA] accession: PRJEB20632). S3 Table indicates the number of assembled transcripts constructed for each sample along with the major N50 and maximum-length values after discarding transcripts shorter than 500 nt in length. For the remainder of this paper, we refer to these 2 assemblies as the separate and combined assemblies, respectively. The assembled *var* genes were analysed at the transcript, domain, and segment or homology block level (Fig 1B). Three of the severe malaria samples had a low percentage of reads mapping to *P*. *falciparum*: SFC025, SFD001 (both cerebral malaria), and SFM009 (hyperparasitemia) (Fig B in S1 Fig, S1 Table). These samples were used for *var* gene assemblies and sequence clustering but were omitted from the differential gene expression analysis, both for *var* and non-*var* genes.

### All gene expression analysis

Two patients (SFU2 and SFU3) were drug treated at admission prior to blood collection, and 4 patients (SFC023, SFM007, IFM012, IFM021) were treated with antimalarials for previous *Plasmodium* infections more than 2 weeks but less than 4 weeks prior to admission. These patients were omitted from the differential expression analyses of the total transcriptomes. Significant differences were identified in the expression of genes between severe and uncomplicated cases of malaria. After accounting for library size, parasite life cycle, and other unwanted sources of variation, 358 genes were found to be differentially expressed after multiple testing correction (*p* = 0.1, limma/Voom pipeline [55,56]). A full list of genes with relevant log fold changes and *p*-values can be found in S1 Data.

**Fig 2:**
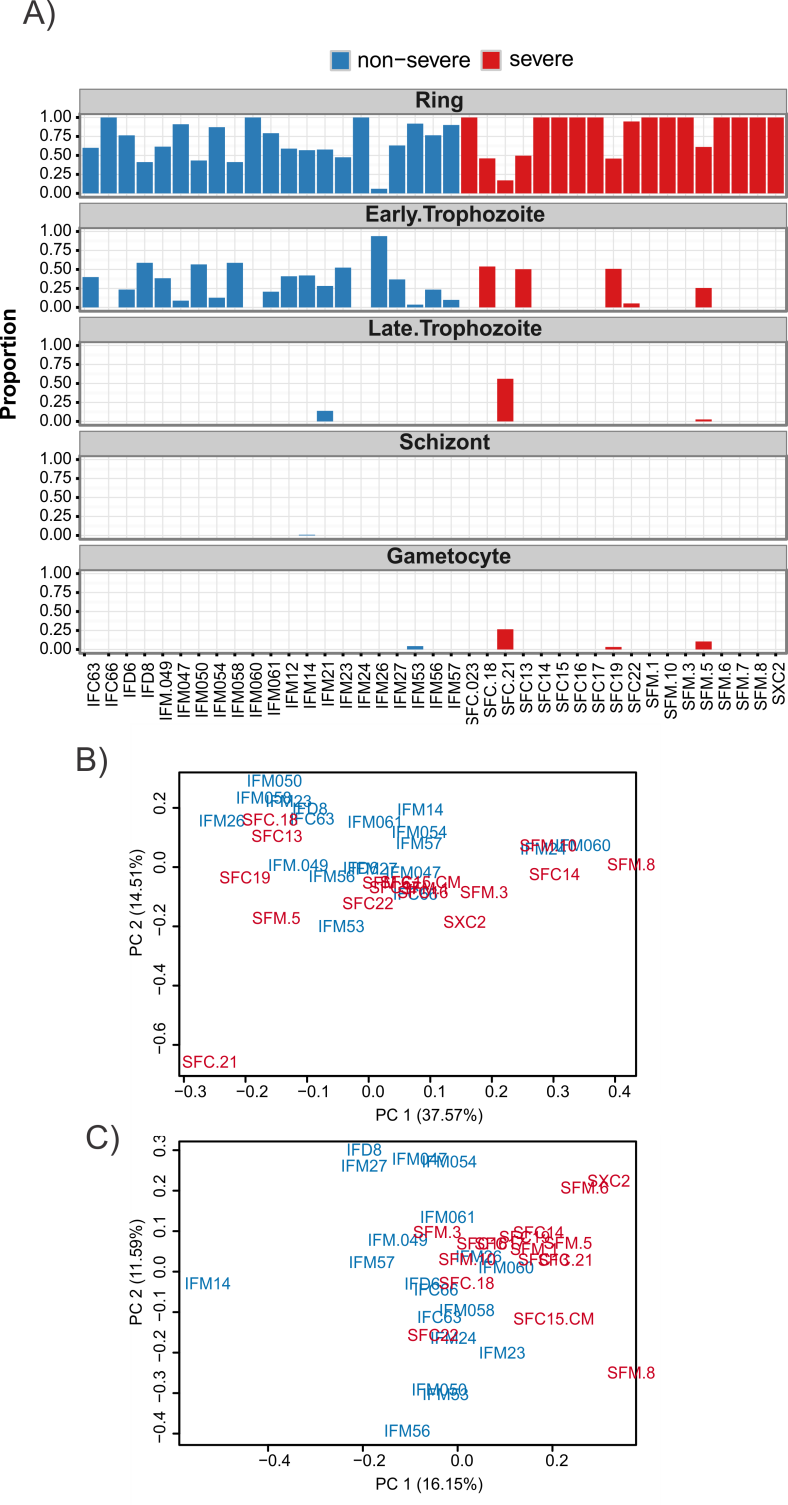
Genome-wide analysis of RNAseq data using 3D7 annotation. (A) Estimated stage proportions for each sample. The mixture model was constrained to require that each sample be made up of a combination of ring, early trophozoite, late trophozoite, schizont, and gametocyte stages. Consequently, the columns in this barplot must add to 1 for each sample. A small bias towards the early trophozoite appears in the nonsevere malaria samples. Sample SFC21 also appears to be an outlier due to its higher proportion of late-stage and gametocyte parasites, a finding which was confirmed by microscopy. Plotted proportions are available in S2 Data. (B) A PCA plot of read counts normalised for library size (read counts are available in S2 Data). Samples are coloured by phenotype, red for severe and blue for nonsevere. Some separation by disease severity phenotype is evident; however, staging effects are apparent as is seen in the outlying position of sample SFC21, which has been identified as having more late-stage and gametocyte parasites. (C) A PCA plot of read counts normalised for library size, staging effects, and other unwanted batch effects using the novel mixture model along with 3 unwanted factors of variation estimated by RUV4 (normalised read counts are available in S2 Data). Sample SFC21 has been appropriately dealt with and a better separation of the samples by disease phenotype can be observed. PC, principal component; PCA, principal component analysis; RUV, Remove Unwanted Variation.

A mixture model was used to account for parasite life cycle. A constrained linear model was fit using published data [57] to estimate the proportion of ring, early trophozoite, late trophozoite, schizont, and gametocyte stages present in each sample (Fig 2A, S2 Data). This approach returns similar results to the maximum likelihood approach of [5] and is comparable to the approach of [58], which focused on microarray data. The mixture model correctly identified sample SFC21 as having a higher proportion of gametocytes, a finding that was confirmed by microscopy. Trimmed mean of M values (TMM) normalisation [59] was used to account for library size, with samples SFC025, SFD001, and SFM009 excluded due to insufficient coverage.

The proportion of parasites present at the ring stage—as well as 3 factors of unwanted variation estimated using the R package ruv [60]—were used to account for life cycle and other unwanted batch effects. Differential expression testing was conducted using the limma/Voom pipeline [55,56]. The impact of including these covariates in the model is evident in the Principal Component Analysis (PCA) plots (Fig 2B and 2C, S2 Data). The choice of covariates strikes a balance between testing power and accounting for unwanted variation. The PCA plots indicate that the outlying SFC21 sample has been accounted for. Furthermore, the separation between the severe and uncomplicated cases shows that, after accounting for variations due to parasite life cycle, significant differences exist between the phenotypes.

### Differences between severe malaria transcriptomes

The severe malaria transcriptomes could be separated by profile of differentially expressed genes into 2 principal clusters—S1and S2 (S3 Fig), which was consistent with previous reports of clinical isolates and severe malaria [6,7]. This suggests that severe malaria can be caused by parasites in different physiological states. A previous report also found that median parasitemias differed between severe malaria clusters [7]; the median parasitemias in the clusters in this study were also suggestive of a difference (*p* = 0.0755 Mann Whitney test; parasites/μl median, IQR, S1: 43,040; 5,880; 259,378; S2: 786,316; 212,708; 1,095,789). However, the severe malaria transcriptomes did not cluster by clinical syndrome (Fisher’s exact test, all *p* > 0.12).

### Analysis of differential gene expression

**Fig 3:**
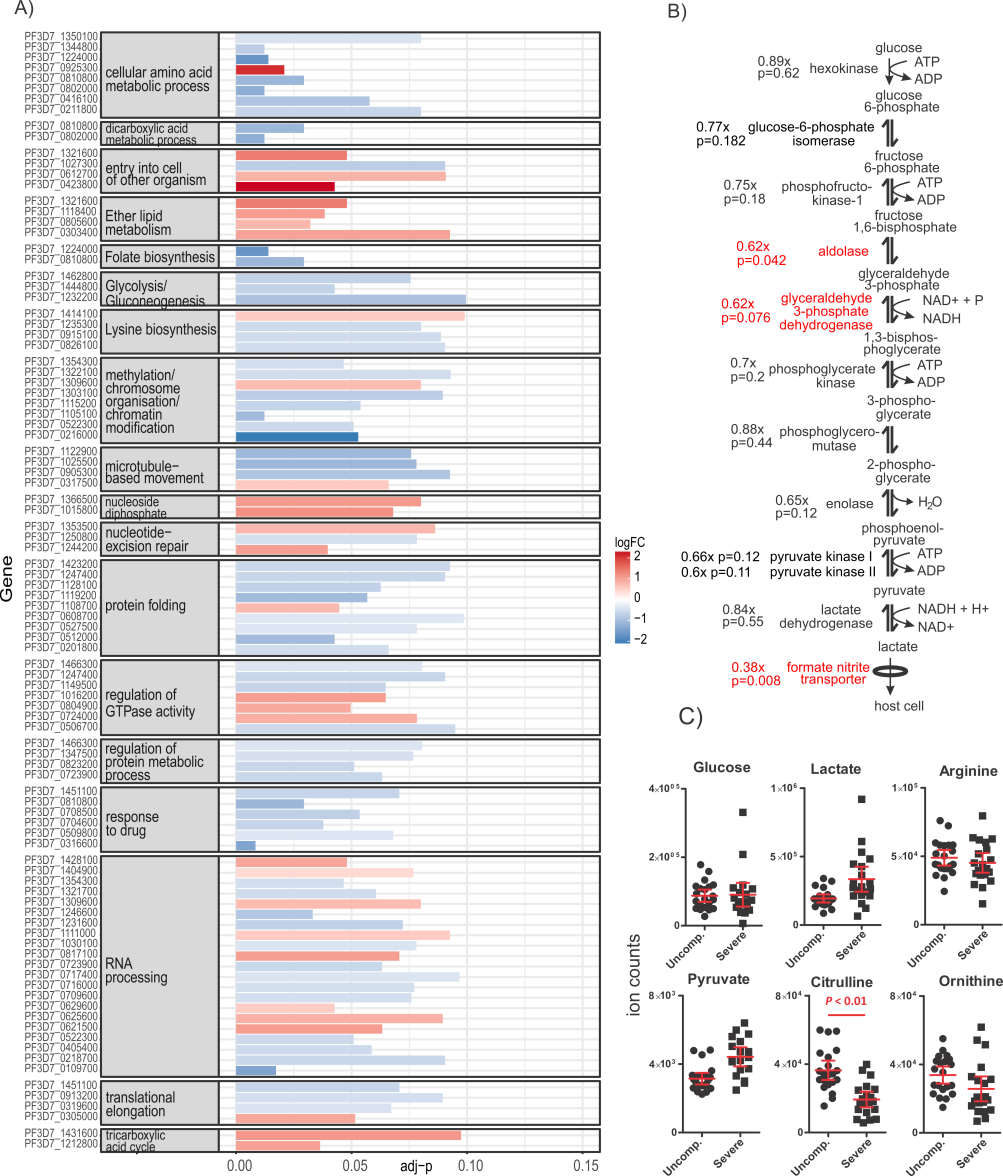
Gene sets enriched in deregulated genes in severe malaria. (A) Summary of highly ranked GO and KEGG gene annotation pathways that included significantly deregulated genes in severe malaria. Only gene sets that contained more than 1 deregulated gene are shown; deregulated gene set data available in S3 Data, deregulated genes available in S1 Data. (B) The glycolysis pathway in *P*. *falciparum* in severe malaria. Fold-change in gene expression in severe malaria relative to uncomplicated malaria (x) and *p*-value for the fold-change are indicated beside genes. Genes that were significantly (adjusted *p <* 0.1) down-regulated in severe malaria are indicated in red. (C) LC-MS metabolomic analysis of plasma samples from patients with severe and uncomplicated malaria. Ion counts for metabolites commonly affected by malaria are presented; data available in S4 Data. adj-p, adjusted *p*; GO, Gene Ontology; KEGG, Kyoto Encyclopedia of Genes and Genomes; LC-MS, liquid chromatography–mass spectrometry; logFC, log fold-change; uncompl, uncomplicated.

The 358 genes differentially expressed in severe malaria were from diverse functional pathways and revealed a distinct severe malaria parasite transcriptome (S3 Fig, S1 Data). Biological pathways annotated as Gene Ontology (GO) biological process terms or Kyoto Encyclopedia of Genes and Genomes (KEGG) were ranked using a hypergeometric test, and those with *p <* 0.1 are considered. Terms relating to glycolysis, histone methylation, folate metabolism, and protein folding ranked highly and included genes down-regulated in severe malaria, whilst pathways relating to the tricarboxylic acid (TCA) cycle, nucleoside diphosphate (pyrimidine) metabolism, and regulation of guanosine triphosphatase (GTPase) activity included genes up-regulated in severe malaria (Fig 3A, S3 Data). In addition, genes involved in PfEMP1 transport and a gene involved in regulation of *var* genes were down-regulated in severe malaria. This suggested that *var* gene expression was modulated but PfEMP1 surface presentation was reduced. Several GO and KEGG pathways that ranked highly included deregulated genes that were not functionally related in a coherent manner and will not be discussed further.

### Parasite carbon metabolism

Parasites isolated from patients with severe malaria had significantly down-regulated genes included in the KEGG pathway ‘Glycolysis/gluconeogenesis’. Significant decreases were observed in transcript levels of 3 glycolytic enzymes (0.52- to 0.62-fold the levels in parasites that caused uncomplicated malaria) (all adjusted *p <* 0.1) (Fig 3B, Fig 3A, S1 Data, S3 Data). These were aldolase, glyceraldehyde 3-phosphate dehyrogenase, and mitochondrial dihydrolipoyl dehydrogenase (LPD1) that converts glycolytic pyruvate to acetyl coenzyme A (acetyl-CoA). Expression of most other enzymes in this pathway trended down (with 3 adjusted *p* ≤ 0.12) (Fig 3B). The lactate transporter (also known as the formate nitrite transporter [61]) was also down-regulated in parasites causing severe malaria (0.33-fold *p =* 0.009). Together, these data suggest that parasites associated with severe malaria have decreased transcription of genes involved in aerobic glycolysis.

Our results confirm and extend previous analyses on the transcriptional regulation of enzymes involved in central carbon metabolism in clinical isolates [8]. In particular, Daily et al. [6] described a cluster of *P*. *falciparum* clinical isolates that exhibited a distinct, starvation-like response, characterised by decreased transcription of genes involved in glycolysis and increased transcription of genes encoding enzymes involved in the TCA cycle, which is similar to the transcriptional signature we observed from parasites linked to severe malaria samples (S4 Table). In contrast, parasites isolated from patients with severe malaria in a subsequent study had a transcriptional profile that was more consistent with a glycolytic phenotype [7]. However, neither study directly compared transcriptional profiles from parasites causing severe and uncomplicated malaria [6,7].

### Severe malaria patients’ metabolic profile

To determine whether nutrient availability was contributing to the reduced expression of genes encoding glycolysis enzymes by the parasites causing severe malaria, metabolite levels in plasma samples from the severe and uncomplicated malaria patients were analysed by untargeted liquid chromatography–mass spectrometry (LC-MS) analysis. Thirty-five metabolite peaks differed significantly between the plasma of patients with severe and uncomplicated malaria (*p <* 0.01, Benjamini-corrected; S4 Data). These included 7 metabolites—provisionally identified as lipids—and citrulline (confirmed with an authentic standard), which was depleted in the patients with severe malaria. Citrulline recycling to arginine contributes significantly to nitric oxide (NO) synthase substrate availability and thereby NO bioavailability in malaria. Low citrulline is therefore likely to contribute to the hypoargininenia, impaired NO bioavailability and endothelial dysfunction found in both adults and children with severe malaria, in both Melanesian [62] and African [63–65] populations. The plasma levels of glucose and lactate were similar in patients with uncomplicated and severe malaria (Fig 3C), suggesting that the down-regulation of parasite glycolysis in the patients with severe malaria is not a direct response to reduced availability of blood glucose. Blood glucose concentrations were also similar in individuals harboring parasites with or without the proposed starvation transcriptional pattern described by Daily et al. [6].

### Alternative pathways of carbon metabolism

Glucose-starved yeast [66] and clinical *P*. *falciparum* isolates with the proposed starvation response-like transcriptome both increased transcription of TCA cycle enzymes [6]. Similarly, in severe malaria, the GO category ‘tricarboxylic acid cycle’ included 2 genes up-regulated more than 2-fold in severe malaria (both adjusted *p <* 0.097): the Fe_2_S subunit of the mitochondrial TCA cycle enzyme succinate dehydrogenase and the putative succinyl CoA synthetase β subunit. Aconitase was also up-regulated more than 1.6-fold (adjusted *p =* 0.156); however, no significant differences in expression of the other TCA cycle enzymes were observed (*p*-values > 0.2). We have previously shown that *P*. *falciparum* asexual blood stages primarily sustain TCA cycle fluxes and low-level oxidative phosphorylation by catabolizing glutamine [67] up-regulating the TCA cycle. However, key enzymes in glutamine utilisation were either down-regulated (glutamate dehydrogenase down 0.4-fold, *p =* 0.012) or unchanged (NADP-specific glutamate dehydrogenase, aspartate transaminase, glutamate synthase, malate dehydrogenase, phosphoenolpyruvate carboxylase, and branched chain ketoacid dehydrogenase complex [BCKDH] subunits E1β and E2) in isolates from patients with severe malaria, indicating that these parasites are unlikely to exhibit a significant switch to mitochondrial respiration. Overall, these data suggest that parasites associated with severe malaria were not compensating for decreased glycolysis by increasing oxidation of pyruvate in the TCA cycle and may be metabolically less active than parasite isolates associated with uncomplicated malaria.

### Methylation and lysine degradation

The GO term ‘methylation’ and a number of subsidiary GO terms relating to histone methylation included genes down-regulated in parasites causing severe malaria. The down-regulated genes included a putative histone S-adenosyl methyltransferase and 2 of the 10 SET-domain lysine methyl transferases found in *P*. *falciparum* (SET2 and PfSET7). Levels of SET3 and a putative protein arginine N-methyltransferase 1 (PfPRMT1) were also suggestive of down-regulation (both adjusted *p <* 0.11, <0.73-fold). PfSETvs or SET2 plays an important role in regulating expression of *var* genes (see below). PfSET3 and PfSET7 appear essential for blood-stage growth [68], and PfSET7 can methylate H3 but is localised to the cytoplasm in asexual blood stages [69]. PfPRMT1 probably methylates cytoplasmic and nuclear proteins including methylations of histone 4 that are involved in gene activation [70]. These data suggest that histone methylation pathways involved in gene regulation were down-regulated in severe malaria. Genes involved in chromatin modification were also deregulated by parasites previously reported to have caused high parasitaemia infections [8]. Severe malaria is known to elicit gametocytogenesis, and heterochromatin structure dependent on histone methylation is known to repress the gametocytogenesis transcription factor ApiAP2G [71], so down-regulation of histone methylation would be consistent with induction of gametocytogenesis in severe malaria.

### Folate and nucleoside metabolism

The KEGG term ‘folate biosynthesis’ and a number of related GO terms included 2 genes down-regulated in severe malaria: dihydropteroate synthetase (DHPS) and guanosine triphosphate (GTP) cyclohydrolase. GTP cyclohydrolase is the first and rate-limiting enzyme in the folate pathway and therefore is essential for DNA and protein synthesis. Aspartate carbamoyltransferase (ATCase) was also down-regulated; it is the second enzyme in the pyrimidine biosynthetic pathway; and whether it is rate limiting in *P*. *falciparum* is unknown, but it is so in bacteria [72]. These changes suggest that nucleoside biosynthesis may be decreased in severe malaria, consistent with lower growth rate and/or reduced metabolism. A number of GO pathways related to nucleoside diphosphate and pyrimidine metabolism included 2 genes up-regulated in severe malaria: nucleoside diphosphate kinase (NDK) and the putative small subunit of ribonucleotide reductase. These 2 genes are central to ribonucleoside triphosphate (NTP) and deoxyribonucleoside triphosphate (dNTP) synthesis. Although up-regulation of these enzymes suggests increased DNA synthesis, the down-regulation of key enzymes in the folate and pyrimidine pathways instead indicates that a diminished nucleoside pool is subject to increased flux through ribonucleoside diphosphate (NDP) to dNTP metabolism.

### Translation and protein folding

The GO term ‘translational elongation’ included 3 down-regulated elongation factor genes suggesting decreased protein production. The GO term ‘protein folding’ included 9 down-regulated genes, including the HSP70 interacting protein (HIP), the peptidyl-prolyl *cis-trans* isomerase cytochrome P450 52 (CYP52)—which has in vitro chaperone activity (Marin-Menendez, 2012)—an FK506 binding protein (FKBP)-type peptidyl-prolyl isomerase, and the PfEMP1 transport–associated KAHsp40. Functionally related proteins outside this pathway were also down-regulated, including the hsp70/hsp90 organising protein (HOP), HSP70-x (see below), and the essential PfHsp110c, which is important for preventing heat-induced aggregation of the many *P*. *falciparum* Asn repeat rich proteins during fever [73]. Overall, down-regulation of these genes indicated a decreased stress response or generalised, decreased protein processing.

### Regulation of GTPase activity

The GO category ‘regulation of GTPase activity’ and related GO categories included 3 GTPase-activating protein genes that were up-regulated in severe malaria, 2 of which were specific for Rab GTPases. This would be consistent with decreased Rab GTPase trafficking regulatory activity and therefore decreased vesicular transport. Two genes involved in vesicle transport were down-regulated; these were SNAP proteins, which is involved in dissociation of the Soluble NSF (N-ethylmaleimide-sensitive factor) Attachment Protein Receptor (SNARE) complex, and choline-phosphate cytidylyltransferase (CCT), which is rate limiting for synthesis of the major *P*. *falciparum* membrane phospholipid, phosphatidylcholine [74]. Four genes involved in vesicle transport were up-regulated. These included 2 proteins involved in endoplasmic reticulum (ER) to Golgi transport, the trafficking protein particle complex subunit 5 (TRAPPC5), and the SNARE protein PfGS27; the retrieval receptor for ER membrane proteins, which is required for anterograde vesicular transport; and the vacuolar protein sorting–associated protein 45 that is implicated in vesicle transport from the Golgi to endosomes or the food vacuole. Overall, the probable decreased trafficking activity of several Rabs and deregulated vesicle transport processes suggest deregulated protein trafficking in severe malaria.

### PfEMP1 and *var* regulation

Multiple genes involved in PfEMP1 biology were down-regulated in severe malaria. These included PfSETvs—which methylates lysine 36 on histone 3, is required for *var* gene silencing [68], and is involved in normal *var* switching [75]. The knob-localised KAHRP—which binds PfEMP1 and the cytoskeleton—and the lysine-rich, membrane-associated PHISTb protein (LyMP) (PF3D7_0532400) are both required for optimal binding of PfEMP1 to (some) receptors [76] and were amongst the most down-regulated genes in severe malaria. Also down-regulated were the following: the Maurers cleft proteins SBP1 and REX1, which are required for proper Maurer’s cleft organization and PfEMP1 transport to the erythrocyte surface [77–79]; Heat shock protein 70-x, which forms a complex with Hsp40 in the red blood cell cytosol and is possibly involved in PfEMP1 transport [80]; and KAHsp40, which binds PfEMP3 and KAHRP and colocalises with knob-associated proteins [81]. Therefore, we observed probable mechanistic drivers of modulated *var* regulation and decreased transport of PfEMP1 to the parasite surface. GO categories that were highly ranked due primarily to inclusion of deregulated 3D7 *var* genes were not reported because 3D7 *var* genes were not present in the clinical isolates.

### Surface proteins

Several parasite surface proteins with established functions unrelated to ring-stage parasites were highly up-regulated in severe malaria. The second most up-regulated gene encoded the glycosylphosphatidylinositol-anchored cysteine-rich protective antigen (CyRPA) that anchors the critical invasion protein *Plasmodium falciparum* reticulocyte-binding protein homolog 5 (PfRh5) to the surface of the merozoite [82]; the seventh most up-regulated gene was the merozoite surface-located 6-cysteine protein P41 [83], and the 14th most up-regulated gene was sporozoite invasion-associated protein-2 (SIAP-2). This gene is expressed at low levels in blood-stage cultures but at high levels on the surface of sporozoites, and it appears to be important for hepatocyte traversal [84]. The serpentine receptor 10 was also up-regulated. It is most closely related to receptors that transduce external stimuli in other organisms [85].

### *var* gene expression analysis

There was no difference between severe malaria and uncomplicated malaria in total *var* gene expression, i.e., the number of reads that mapped to de novo–assembled *var* genes (normalised for number of total reads that mapped to all genes; Welch 2-sample *t* test, *p =* 0.28). Differential expression analysis was conducted at the *var* multidomain transcript, individual domain, and segment levels because associations between *var* expression and severe disease have been previously detected separately at each of these resolutions. At each level, significant, differentially expressed sequences were identified. Additionally, the resulting sequence transcripts, domain classification, and segments were found to better distinguish severe and nonsevere cases of malaria than previous *var* gene classifications [14,39,46,48].

Fig B in S4 Fig illustrates a PCA plot of normalised read counts annotated to the transcripts from the combined sample *var* gene assembly. By comparing it to the all-gene PCA plot (Fig 2C), it is evident that *var* gene expression differentiates severe cases of malaria. The severe cases are more tightly clustered together than the nonsevere.

S5 Data lists all the separate sample assembly transcripts along with whether they were significant at the transcript, domain, or segment level. A number of transcripts had domains and segments that were significantly associated with disease severity when the transcript itself was found not to be significantly associated with severe disease. This highlights the importance of investigating the *var* gene sequences at multiple resolutions.

### Transcript level

**Fig 4:**
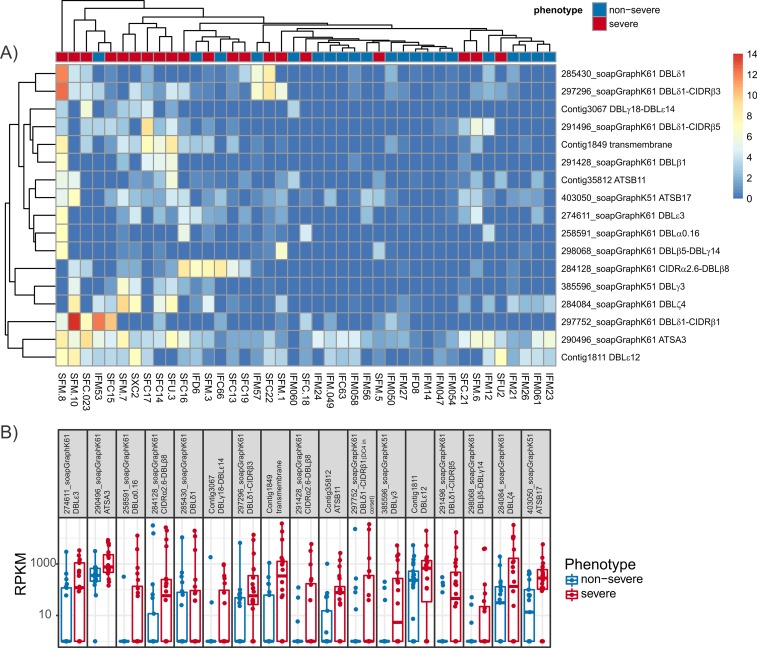
Analysis of RNAseq data at the level of *var* gene transcripts: Combined assembly. (A) Expression levels of transcripts from the combined sample assembly found to be up-regulated in severe disease. Samples and clusters have been grouped using complete linkage hierarchical clustering (raw read counts available in S6 Data). (B) Expression levels of transcripts from the combined sample assembly found to be up-regulated in severe disease; values for all samples and the IQR and median are indicated. RPKM is reads per kb of transcript per million reads mapped to total *var* transcripts (RPKM available in S6 Data). IQR, interquartile range; RPKM, Reads Per Kilobase of transcript per Million mapped reads.

In the combined sample assembly (S6 Data), 53 transcripts were found to be differentially expressed using the default DESeq2 pipeline [86]. Of these, 17 are up-regulated in severe malaria (*p <* 0.05) (Fig 4A). The expression profiles of the up-regulated transcripts from the combined assembly differentiated the samples based on severity (Fig 4A, S4 Fig, panel B). Amongst the transcripts up-regulated in severe malaria, the extracellular domains most highly expressed in severe malaria were a DBLζ4 (284084_soapGraphK61), a DBLε3 (274611_soapGraphK61), and a DBLε12 (Contig1811) (Fig 4B). The up-regulated transcripts included a transcript that contained DBLβ5-DBLγ14 (298068_soapGraphK61); DBLγ14 has only been found in DC6 [14], and its expression was recently associated with severe disease [34]. In 7 *P*. *falciparum* genomes [14], the tandem combination DBLβ5-DBLγ14 was detected only in the 3D7 gene PFL0020w, which is expressed by 3D7 parasites selected for adhesion to ICAM1 [87]. Another of the up-regulated transcripts contained DBLγ18-DBLε14 (Contig3067); this tandem domain arrangement was only detected twice in the 7 sequenced genomes but was not part of any DCs. The remaining transcripts were either single domains or common tandem domain arrangements. A transcript incorporating DC5 (DBLδ5-CIDRβ3-DBLβ7-[DBLγ4]) (contig12688) was also up-regulated in severe malaria (*p =* 0.0537). DC5 was up-regulated in severe malaria in Africa [48] and expressed in a cerebral malaria case in Papua New Guinea [88].

**Fig 5:**
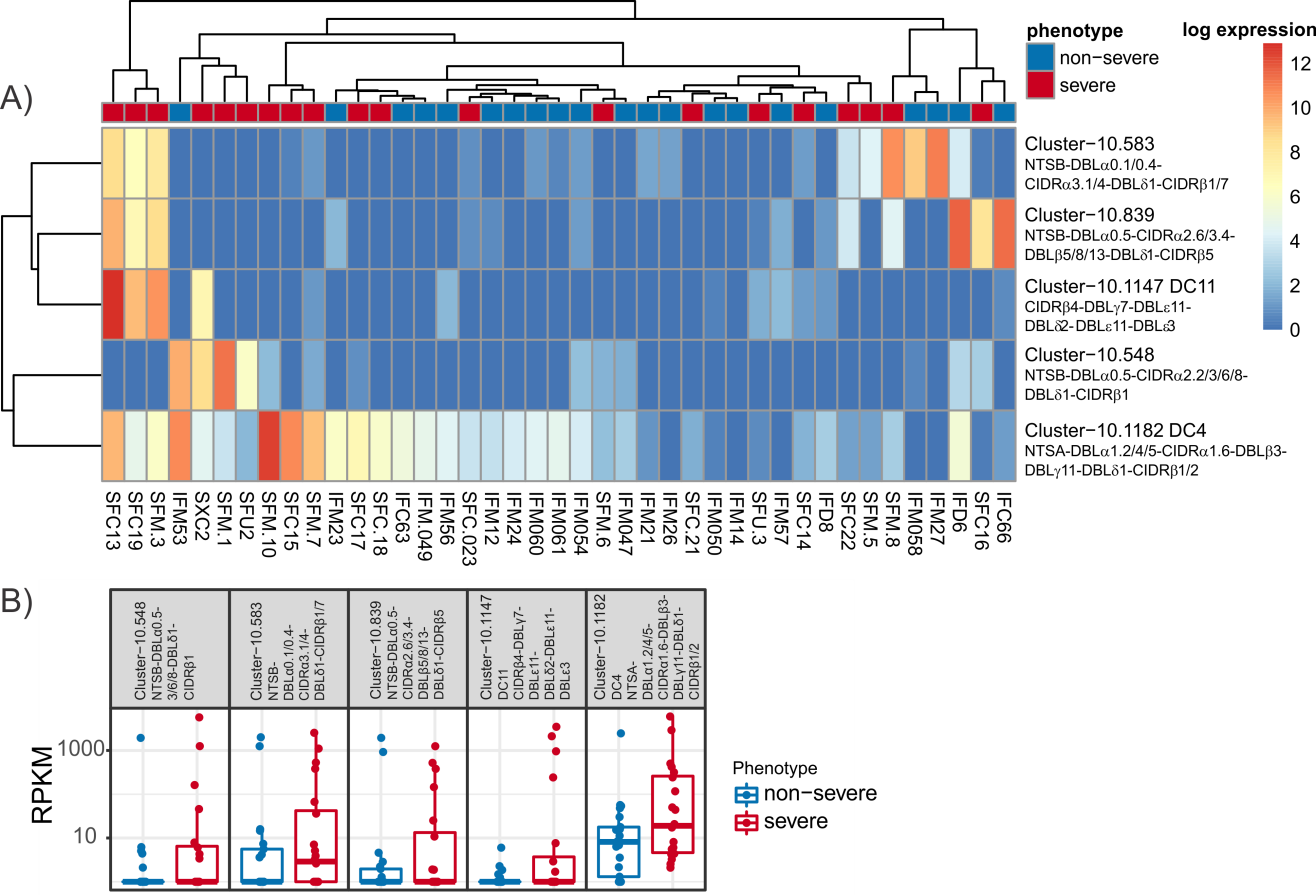
Analysis of RNAseq data at the level of *var* gene transcripts: Separate assembly. (A) Expression levels of clusters identified by Corset found to be up-regulated in severe disease. Samples and clusters have been grouped using complete linkage hierarchical clustering. Raw read counts are available in S7 data. (B) Expression levels of clusters identified by Corset found to be up-regulated in severe disease. Values for all samples and the IQR and median are indicated and are available in S7 Data. RPKM is reads per kb of transcript per million reads mapped to total *var* transcripts. IQR, interquartile range; RPKM, Reads Per Kilobase of transcript per Million mapped reads.

**Fig 6:**
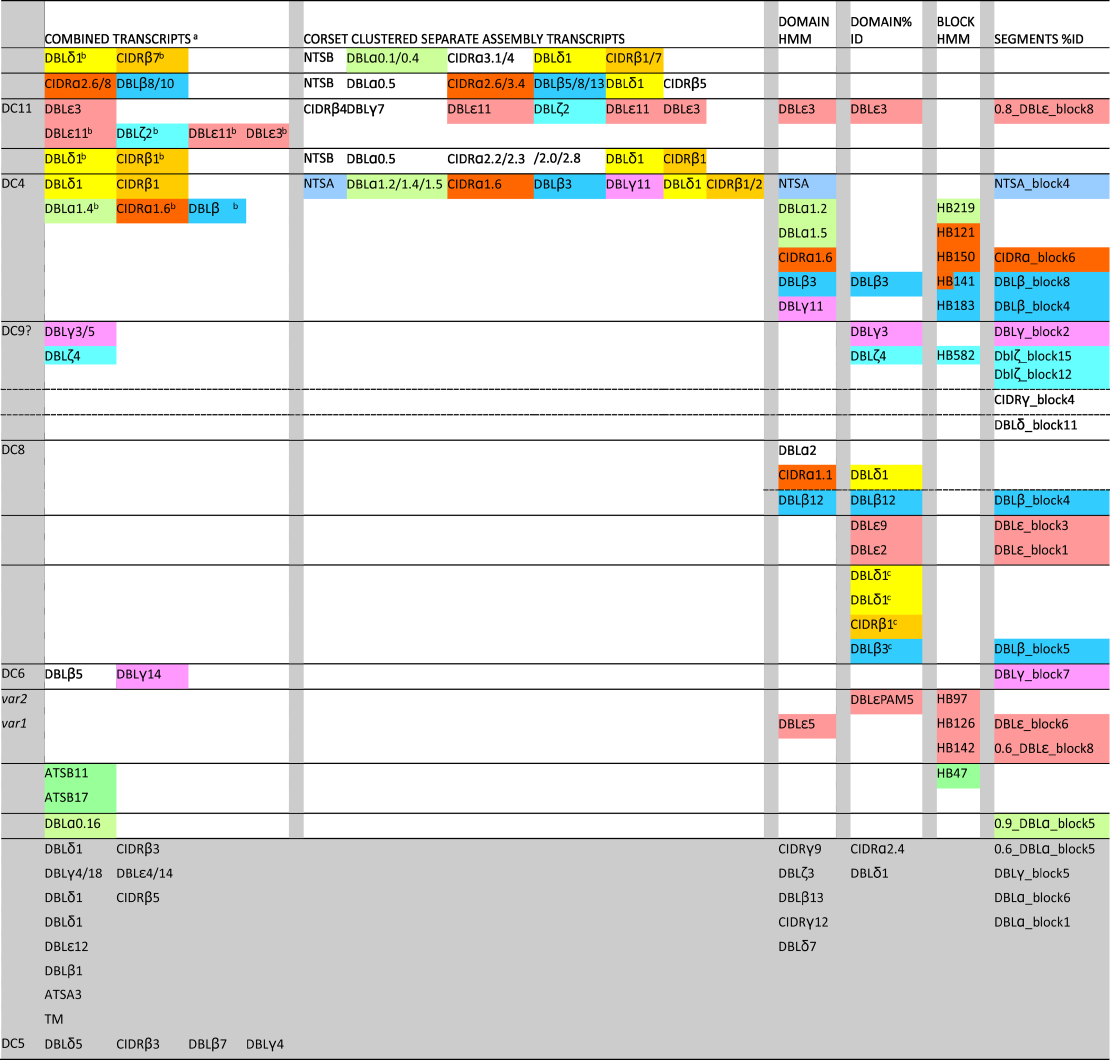
Summary of PfEMP1 transcripts, domains, and segments that were up-regulated in severe malaria. Sequences up-regulated in severe malaria are organised in columns for each analysis method separated by grey bars. Multiple domains found in the same single transcripts from the combined or separate assemblies are on a single row. Closely related sequences found in multiple analyses are colour coded for each of the major domain types and are grouped together across analyses by unbroken horizontal lines. Domains and/or segments that clustered together by expression profile in multiple individuals within a single analysis are also grouped by unbroken horizontal lines. Grey shaded sequences at the bottom of the diagram are unrelated to each other. For example, in the case of DC4, 2 transcripts from the combined assembly were amongst the closest BLAST hits to the DC4-like transcripts from the CORSET cluster of the separate assembly; 6 domains and 5 blocks identified by HMM in the separate assembly are found in DC4 domains; and clusters for 1 domain and 4 segments identified by hierarchical analysis contained DC4 domain sequences, including those from the DC4-like transcripts from the CORSET cluster of the separate assembly. ^a^Combined assembly transcripts up-regulated in severe malaria were all adjusted *p <* 0.05 except for domains marked ^b^ (adjusted *p <* 0.153). Domains HMM and blocks HMM were identified using the HMM of [14]. Domains and segments %ID were identified using the novel hierarchical approach developed for this study. ^c^Non–DC8-like DBLδ1 and non–DC4-like DBLβ3 that clustered by expression profile in the same patients with a highly conserved CIDRβ1. A dashed line separates DBLβ12 from DC8 because DC8 typically contain DBLβ12, but these DBLβ12 formed a phylogenetic cluster with non-DC8 DBLβ12. Dashed lines separate putative DC9 components because transcripts containing all components were not up-regulated in the combined assembly or the Corset analysis, but the clusters from which the up-regulated segments were identified contained multiple transcripts carrying the DC9 domains. ATS, acidic terminal sequence; CIDR, cysteine-rich interdomain region; DBL, Duffy binding-like; DC, domain cassette; HMM, Hidden Markov Model; PfEMP1, *Plasmodium falciparum* Erythrocyte Membrane Protein 1; TM, transmembrane.

Corset [89] groups transcripts together based on the number of reads that multi-map between them whilst ensuring transcripts are not combined if they have significantly different expression profiles. We used Corset to detect transcripts associated with severe disease in the separate sample assemblies. Associations between severe disease and *var* transcripts can be inferred with greater confidence if identified using multiple approaches. Corset identified 82 differentially expressed clusters in total, of which 5 were clearly up-regulated in severe disease (Fig 5A, S7 Data). These clusters included overlapping, multidomain contigs that spanned DC4 (N-terminal sequence A [NTSA]-DBLα1.2/1.5/1.4-CIDRα1.6-DBLβ3-[DBLγ11-DBLδ1-CIDRβ1/2]) (cluster-10.1182) and DC11 (CIDRβ4-DBLγ7-DBLε11-DBLζ2-DBLε11-DBLε3) (cluster-10.1147). The contigs spanning DC4 were the most abundantly expressed of the clustered contigs up-regulated in severe malaria (Fig 5B). DC4 expression has previously been associated with severe malaria [39], and the DC4 cluster included 2 DC4 transcripts from the cerebral malaria sample SFC15. For each transcript in the separate assembly, its closest basic local alignment search tool (BLAST) [90] hit in the combined assembly was identified. Of the 5 Corset clusters up-regulated in severe malaria, 2 included transcripts with their closest BLAST hit in the 17 up-regulated transcripts from the combined assembly. These were the DC4 cluster—which was homologous to the DBLδ1-CIDRβ1 combined assembly transcript 297752_soapGraphK61—and cluster-10.839 (N-terminal sequence B [NTSB]-DBLα0.5-CIDRα2.6/3.4-DBLβ5/8/13-DBLδ1-CIDRβ5)—which was homologous to the combined assembly transcript 284128_soapGraphK61 (CIDRα2.6-DBLβ8) (Fig 6). The 2 remaining clusters contained transcripts spanning NTSB-DBLα0.1/0.4-CIDRα3.1/4-DBLδ1-CIDRβ1/7 (cluster-10.583) and NTSB-DBLα0.5-CIDRα2.2/2.3/2.6/2.8-DBLδ1-CIDRβ1 (cluster-10.548). These 2 clusters and the DC4 and DC11 clusters were all homologous to additional transcripts that were up-regulated in the combined assembly at an adjusted *p*-value of no more than 0.153 (Fig 6). The elevated *p*-values in the combined assembly analysis can be explained by the heavier multiple testing penalty due to the larger number of transcripts.

### *var* UPS type level

For simplicity, we restricted our analysis at the type level to distinguishing between UPS types A and B/C combined. Expression of the conserved NTS segments allows for these 2 groups to be identified. HMMER3 [91] was used to align the profile hidden Markov models of the domains defined in [14] to the transcripts built from the separate assemblies. Reads that aligned to the regions annotated as either NTSA or NTSB were then used as counts for the respective *var* types. NTSA was more highly expressed in the severe malaria samples than in the uncomplicated malaria samples (Fig A in S4 Fig). This is consistent with previous studies [19–23,34,92].

### Domain level

**Fig 7:**
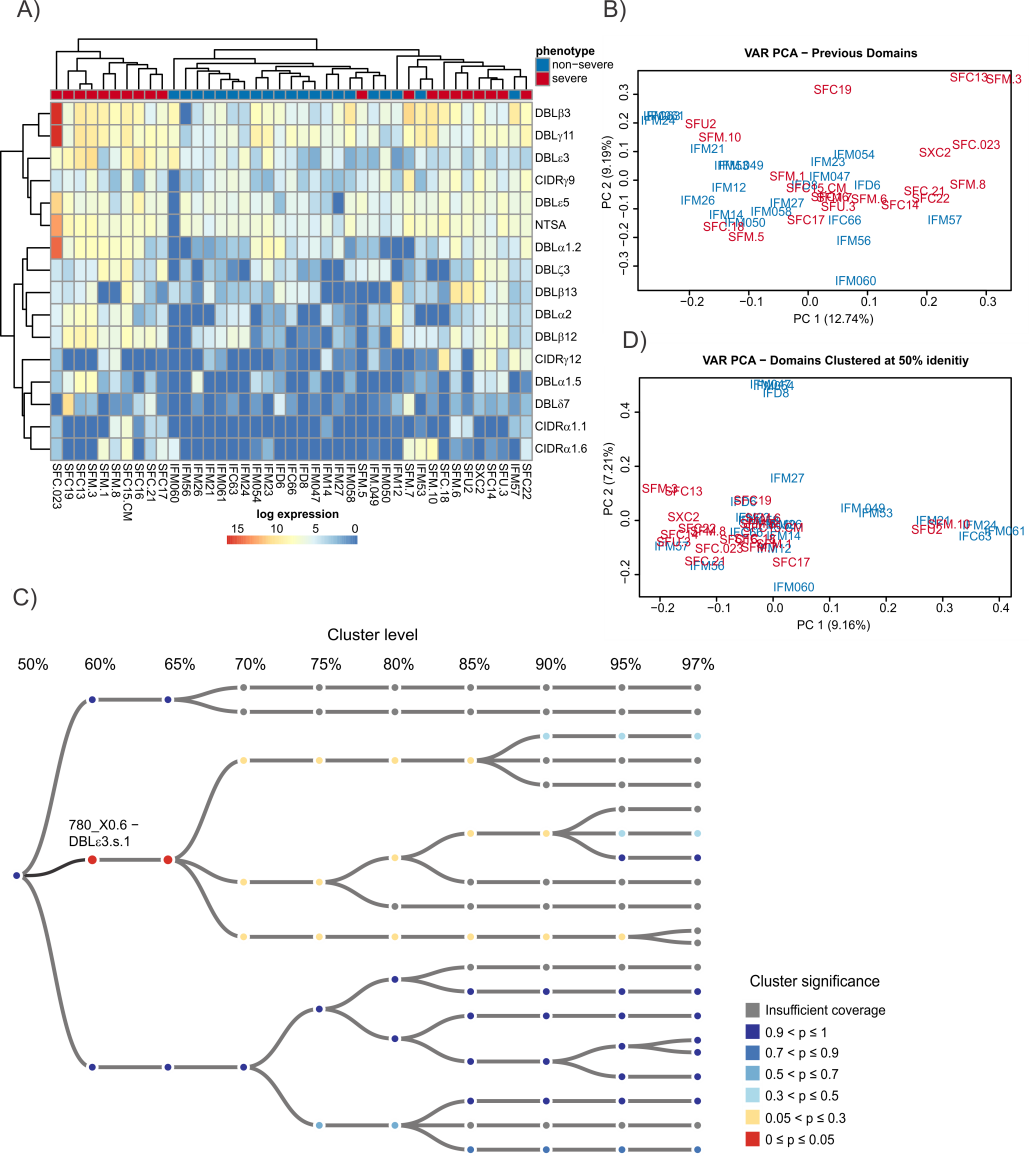
Analysis of RNAseq data via de novo assembly at the level of *var* gene domains. (A) Expression levels of domain subfamilies from [14] found to be up-regulated in severe disease as identified using HMMER3 models. These models were built from the domain sequences of [14]. Samples and clusters have been grouped using complete linkage hierarchical clustering. (B) PCA plot of read counts that align to domain regions of the de novo–assembled transcripts identified using HMMER3 models. There is less separation by phenotypes in this plot than was observed at the whole-transcript–and all-gene–analysis levels. Read count data for Fig 7 is available in S8 Data. (C) An example of the hierarchical clustering tree. Colours represent significance, with red indicating a significant difference in expression after multiple testing correction and blue indicating not significant. Nodes are coloured grey if there is insufficient evidence for them to be considered in the testing either because they have less than 5 samples present or they are marked by DESeq2’s prefilter step. At the 60% identity level, cluster 670_X0.6 becomes significant. This significance is then obscured at the 50% identity level, demonstrating the importance of considering different levels of the hierarchy. (D) Clustering the domain level counts at 50% sequence identity rather than using the previous classifications of [14] improves the grouping of severe samples. At 50% identity, the severe samples are grouped more closely together, suggesting that they have more in common than the nonsevere samples; transformed read count data available in S9 Data. PCA, principal component analysis; RNAseq, RNA sequencing.

**Fig 8:**
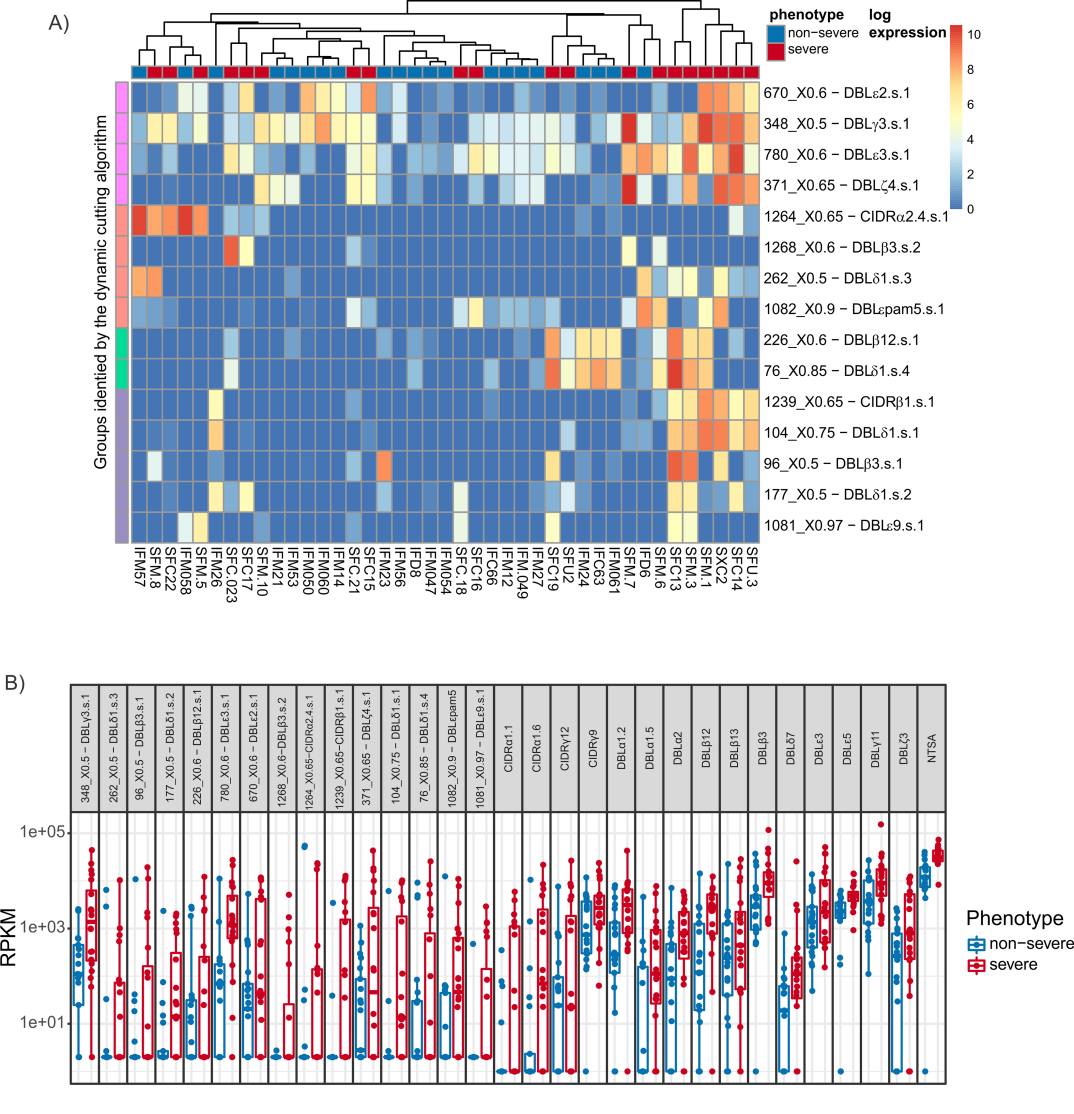
Analysis of RNAseq data via de novo assembly at the level of *var* gene domains: Hierarchical analysis. (A) Expression levels of the domain clusters identified using the hierarchical approach. Samples and domains are grouped using complete linkage hierarchical clustering. The colourings on the left indicate notable groups identified using the hierarchical cutting algorithm of [95]. The clusters are also annotated with the domain model of [14] that they most closely resemble. Raw read counts are available in S9 Data. (B) Expression levels of the domain clusters identified using the hierarchical approach, values for all samples, and the IQR and median are indicated. RPKM is reads per kb of domain per million reads mapped to total *var* domains (RPKM data available in S9 Data). IQR, interquartile range; RPKM, Reads Per Kilobase of transcript per Million mapped reads.

The domain models of [14] were first investigated using the same approach as the type-level analysis. Of the 149 domain classifications identified in the transcripts, 16 were found to be significantly up-regulated in severe malaria using the default pipeline of DESeq2 [86] (S8 Data, Fig 7A). Some previously described associations between expressed *var* sequences and severe malaria were confirmed, adding confidence to our analysis. These included up-regulation in severe malaria of CIDRα1.1 and CIDRα1.6, which bind EPCR [35] and are often found in DC8 and DC4, respectively [32,34,45,46,48,49]; DBLα2, which is restricted to DC8; and DBLβ12, which binds gC1qR [45] and is invariably found in DC8. DBLβ3 was also up-regulated; it can bind ICAM1 and is found in—but is not restricted to—severe malaria–associated DC4 [39] and DC8. The domain subtypes DBLβ3, CIDRα1.6, DBLα1.2, DBLα1.5, and DBLγ11 that were all up-regulated in this analysis were also part of the up-regulated DC4 transcript in the Corset analysis (Fig 6, Fig 5A, S5 Data, S7 Data). NTSA that is restricted to group A *var* genes was also up-regulated. Despite these clear differences, many of the domains were still expressed in a large number of the uncomplicated samples, e.g., DBLβ3 was far more abundantly expressed than CIDRα1.6 and DBLα1.5, but the latter 2 domains were more clearly differentially expressed in severe malaria (Fig 7A, Fig 8B). Comparing the domain-level PCA plot in Fig 7B with the transcript-level PCA plot in Fig B in S4 Fig showed that the differentiation between severe and uncomplicated malaria samples was less evident at the domain level and suggested that a more accurate classification could be made.

A novel hierarchical approach was developed to identify domains that are associated with severe malaria. Domain regions were first defined using HMMER3 domain models based on the domain sequences identified in [14]. The identified domain regions were then hierarchically clustered using USEARCH [93] as described in the Materials and methods section at sequence identity levels 50, 55, 60, 65, 70, 75, 80, 85, 90, 95, and 97. The counts for each cluster were aggregated up the hierarchical tree, and differential expression was tested at each node. Benjamini-Yekutieli [94] multiple testing correction was performed before the most significant node was successively chosen and added to the list of significant clusters. The children and parents of each node were removed from the list of potential clusters before the next node was chosen in the tree. A more detailed description is given in the Materials and methods section.

This approach attempts to identify the point, or node, in the hierarchical tree that best distinguishes domains associated with severe disease from those that are not. By looking at different levels of the tree, we are able to identify potentially important domain groups that would otherwise remain elusive. Additionally, by grouping domains at various identity levels, we are increasing the sensitivity to domain groups with higher sequence variation such as the DBLδ domain class.

Fig 7C illustrates the advantage of this approach by focusing on a tree related to the DBLε3 domain of [14]. At 70% identity, no clusters are significantly associated with severe disease. However, at 65%, 1 cluster (DBLε3.s.1) becomes significant. This difference is then lost at 50% identity. A PCA plot of the clusters at 50% identity is shown in Fig 7D. It provides a much clearer grouping of the severe samples than the previous domain definitions. Similar groupings are seen at all levels of the tree (S5 Fig).

The tree-building approach identified 70 differentially expressed domain clusters, of which 15 are up-regulated in severe malaria (S9 Data). To investigate possible associations between these 15 clusters, they were grouped based on their expression across the samples using average linkage hierarchical clustering. The dendrogram was then cut using the dynamic height-cutting algorithm of [95]. This identified 4 groups, labelled in the heatmap diagram of Fig 8A. Compared to Fig 7A, these domains provide a clearer distinction between parasites causing severe and uncomplicated malaria. However, the expression levels of these domain clusters were, in general, lower than the levels of the domains identified using the HMMER3 approach of Rask et al. (Fig 8B). This is consistent with the higher level of sequence identity within a domain cluster identified by the hierarchical tree approach. Therefore, fewer sequences per isolate were captured by each domain cluster than were assigned to each domain by the HMMER3 domain models, and the more diverse sequences captured by a HMMER3 model included many that were highly expressed in uncomplicated malaria (Fig 8B).

Examining Fig 8A in more detail reiterated elements of the original domain analysis (Fig 7A and Fig 6) and confirmed previous associations between expressed PfEMP1 domains and severe malaria. Two domain clusters similar to DBLβ3 were up-regulated in severe disease. Domain transcripts from the DBLβ3 clusters were aligned with published DBLβ3 sequences [39] using MUSCLE [96] and clustered using FastTree [97]. Notably, the DBLβ3 domain 4 from DC4 in the *var* gene PFD1235w [39] clustered tightly with 2 of the 4 transcripts from the DBLβ3s.2 cluster we identified but none of the 4 transcripts from the DBLβ3s.1 cluster. The turquoise group of domain clusters includes a cluster with sequences similar to DBLβ12 domains. These DBLβ12 sequences were compared with domains from DC8 but formed a separate cluster to the 2 clusters formed by the DBLβ12 domains found in DC8 [14] (S2 Text). The turquoise group of domain clusters in Fig 8A also contained the domain cluster DBLδ1.s.4, which did cluster closely with 2 DC8 DBLδ1 domains, although DC8 DBLδ1 domains cannot be differentiated from other DBLδ1 domains. Nonetheless, the clustering by expression profile of DBLδ1.s.4 and DBLβ12.s.1 (turquoise group in Fig 8A) suggests that these domains could be part of a single DC8-like *var* in these patients. Unlike all the other analyses employed in this study, the hierarchical approach did not associate up-regulated CIDRα1 sequences with severe malaria. This is presumably a consequence of the low conservation of the CIDRα1 sequences [35] that would not be grouped at the minimum 50% identity threshold employed in the hierarchical analysis. All alignments and trees made with published sequences described above are further described in S2 Text and are available in the Github repository https://github.com/gtonkinhill/falciparum_transcriptome_manuscript.

Fig 8A also identifies domain clusters that have not been previously associated with severe disease. These included DBLγ3.s.1 and DBLζ4.s.1 from the pink group of domain clusters in Fig 8A. Four of the 5 DBLγ3 and 11 of the 12 DBLζ4 sequences previously described were found in tandem in DC9 [14]. These domain clusters were among the most abundant up-regulated in severe malaria (Fig 8B) and were also up-regulated in the combined transcript assembly (Fig 6), although not as a single transcript, so there is no direct evidence that DC9 itself is associated with severe malaria. DBLε3 was also amongst the most abundant domain clusters up-regulated in severe malaria in this analysis (Fig 8B). DBLε3 was also up-regulated in every analysis we performed (Fig 6) and was part of DC11 in the separate assembly transcript Corset analysis, although the DC11 transcripts were all different from the up-regulated DBLε3 transcripts from the individual domain analysis. A cluster similar to DBLεpam5 from the pregnancy malaria-associated gene *var2csa* was also up-regulated.

DBLδ1-CIDRβ1/2/3/5/7 are common arrangements and were up-regulated in severe malaria in both the combined assembly and the Corset analysis of separately assembled transcripts. These domain subclasses are highly variable and thus difficult to distinguish using the previous classifications of [14]. However, the hierarchical approach identified the CIDRβ1.s.1 domain cluster that includes a number of identical domain sequences from different isolates. This finding differs from the high variability noted in previous studies [14]. A single CIDRβ1 from a gene containing DC8 formed a phylogenetic cluster with the conserved severe malaria–associated CIDRβ1 sequence, but CIDRβ1 from other DC8 genes did not (S2 Text). The DBLδ1.s.1 and DBLδ1.s.2 domain clusters did not form phylogenetic clusters with DBLδ1 sequences from DC8 genes (S2 Text) but did cluster by expression profile with the uniquely conserved CIDRβ1.s.1 (purple group Fig 8A) and with the non–DC4-like DBLβ3.s.1, suggesting the existence of *var* genes that carry a unique pathogenic arrangement of domains, including a highly conserved CIDRβ1 sequence. These 4 domains were not the most abundantly expressed in severe malaria but did discriminate strongly between severe and uncomplicated malaria, suggesting that they were strongly associated with severe malaria in a subset of cases (Fig 8A, Fig 8B). Images of the hierarchical trees that make up each of these newly identified domain clusters are provided in the Github repository along with their respective multiple sequence alignments.

The sequence of domains associated with severe malaria by RNAseq was confirmed by Sanger sequencing 34 sequences that were cloned from genomic DNA (gDNA) of patient samples. These included domains identified by hierarchical analysis (13 domains up-regulated and 11 down-regulated in severe malaria), HMMER3 analysis (9 domains up-regulated in severe malaria), or corset analysis (the CIDRα2.6-DBLβ8 tandem arrangement up-regulated in severe malaria) (S10 Data). Every one of these cloned sequences was 100% identical to the cognate sequence assembled from RNAseq. RNAseq quantitation of domains in severe malaria was corroborated by quantitative reverse transcription PCR (Q-RT-PCR) of 10 up-regulated and 3 down-regulated domains. Insufficient RNA was available to test all patient samples, so a subset of patients was tested that included several patients for each domain that had high levels of RNAseq expression of that domain. Q-RT-PCR data correlated with RNAseq Reads Per Kilobase of transcript per Million mapped reads (RPKM) for 12 of the 13 domains (Spearman r all greater than 0.53, all *p* < 0.03) (S6 Fig). Q-RT-PCR and RNAseq of DBLε3.s.1 did not correlate because a number of uncomplicated malaria samples contained high levels of expression by Q-RT-PCR but not by RNAseq. Because Q-RT-PCR works best on small sequences (in this case a 64 bp product) and detects hybridisation rather than actual sequence, the most probable explanation for this discordance is cross-reactive amplification of non-DBLε3.s.1 by the Q-RT-PCR.

### Segment level

Due to the highly variable nature of *var* genes, it is common to focus on the most conserved segments—or blocks—of *var* gene sequence [14,50]. To investigate these conserved regions, we examined 628 homology blocks that were previously defined [14]. Of these, 613 were available for download from the VARDOM server. An approach was also developed comparable to that of [98] to divide the *var* sequences into conserved and variable regions.

The previously defined Homology blocks [14] were clustered and examined using the same approach as for the previously defined domains [14]. HMMER3 [91] profile hidden Markov models were used to annotate the separate assembly transcripts, and read counts were obtained from the aggregate of these annotations for each block. Overall, 16 homology blocks were identified as being differentially expressed (S11 Data). Ten of these (homology blocks 47, 97, 121, 126, 141, 142, 150, 183, 219, and 582) were up-regulated in severe disease. The heatmap in Fig A in S7 Fig indicates that homology blocks 219 and 582 are the most distinct in their expression profiles. Homology block 219 is located in the DBLα1 domain class found in group A *var* genes and has previously been associated with severe malaria and rosetting [51]. Block 582 is usually found after a DBLζ4 domain in DC9. DBLζ4 domains were found to be up-regulated in severe disease in the domain-level analysis. Homology blocks 126 and 142 are found mainly within DBLε5 but also other DBLε subtypes, whilst block 97 is found in DBLε4–8,12,14,PAM5 and DBLγ6,12,16,17 domains. Some of these DBLε subtypes were also identified in the domain-level analysis. Blocks 121 and 150 are found in CIDRα1 domains, and homology block 141 is found at the junction between CIDRα1 and DBLβ1,3,7,12 domains, whilst block 183 is found in DBLβ1,3–5,10–12, domains. As mentioned previously, DBLβ3, DBLβ12, and CIDRα1 domains are associated with DC4 and DC8, which have been associated with severe disease. Finally, homology block 47 is found within the acidic terminal sequence (ATS) of *var* genes. This region does not code for the extracellular part of the protein.

Although differentially expressed blocks are identified, it likely that, as in the domain analysis, a better classification can be made by making use of the novel transcripts. The homology blocks used for this analysis were defined based on conserved recombining regions in the *var* gene genome [14] and not on their relationship to disease severity. This may have obscured conserved regions that are related to severe disease. Furthermore, by focusing on only the most conserved regions, we are potentially ignoring informative—but more variable—regions. Finally, the homology blocks of [14] were inferred from laboratory strains and may not include conserved segments that are unique to severe disease types.

As an alternative, an approach similar to [98] was used to divide up multiple sequence alignments of the major domain classes. Domains identified using HMMER3 [99] were grouped into their major domain classes and aligned using Gismo [100]. Sequence logos of the resulting alignments were generated using skylign [101] (see S8 Fig). Gismo [100] was found to handle the large diversity in the *var* domains better than other aligners. The resulting alignments were then segmented into regions of high and low occupancy. If 7 or more consecutive columns within an alignment had an occupancy greater than 95%, these columns were considered a conserved region. The columns in between these conserved regions were considered variable regions. Regions of high variability are harder to align and consequently result in more gapped alignments. The results were found to be robust to the choice of the occupancy threshold as well as the choice for the number of consecutive conserved columns. This approach produces interleaved regions of higher conservation and diversity. The approach is similar to that proposed by [98]; however, we focus on both the conserved and variable regions. Each domain sequence was then split into segments based on the regions identified. We refer to these segments by their location within the domain from which they originate. For example, DBLα_block2 is the second interleaved region of the DBLα domain class. The segments were then hierarchically clustered within their respective regions and analysed for differential expression in a similar manner to that used for the domains. Due to the short nature of these segments, CD-HIT [102] was used in place of USEARCH [93] because it accounts for the terminal gaps in its definition of pairwise sequence identity. Aside from identifying segments associated with severe disease, an advantage of this approach is that the resulting segments can easily be understood in terms of their relationship to the *var* domains and gene sequence.

**Fig 9:**
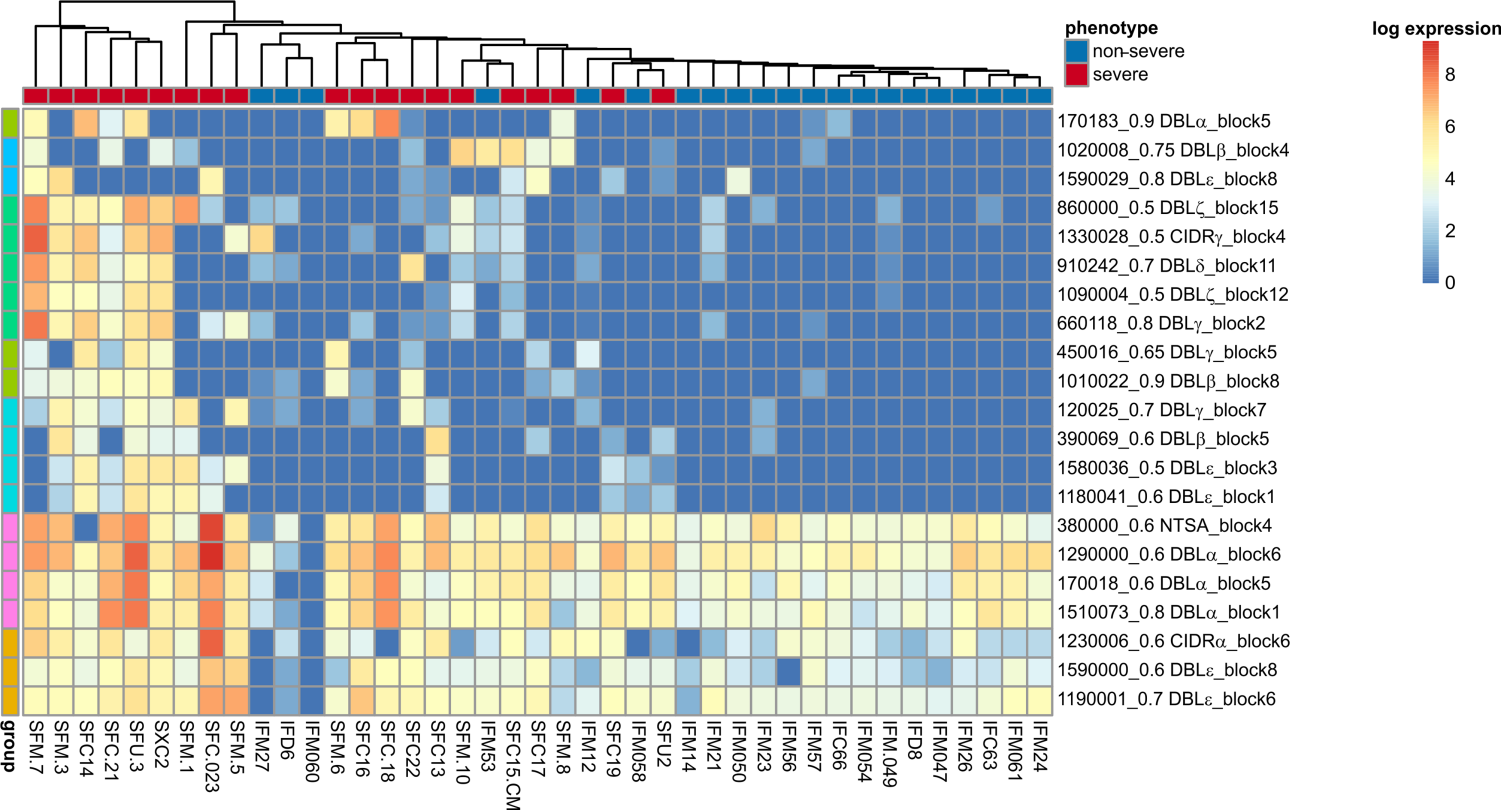
Analysis of RNAseq data via de novo assembly at the level of *var* gene segments. Expression levels of novel conserved segment clusters found to be up-regulated in severe disease. Samples and segment clusters have been grouped using complete linkage hierarchical clustering. The raw read counts that were transformed for this figure are available in S12 Data.

DESeq2 [86] was used to investigate the differential expression of the segments, and Benjamini-Yekutieli [94] correction was used to correct for the multiple dependent tests. Overall, 26 clusters of segments were identified as being differentially expressed, of which 21 were up-regulated in severe disease (S12 Data). Fig 9 indicates the expression levels for each segment cluster across the samples. One lies in segment 4 of the NTSA region and 3 in regions 1, 5, and 6 of the DBLα1 domain class of group A *var* genes. A single up-regulated cluster (170183_0.9 DBLα_block5) lies in segment 5 of DBLα0.1 from non–group A *var* genes.

A cluster from region 6 of the CIDRα domain class had a similar expression profile to those segment clusters from the DBLα domain class. The cluster was also mostly made up of segments from CIDRα1.4 and 1.8 domains that have been associated with severe disease [32–34,46,49]. Two DBLε segment clusters from regions 6 and 8 derived primarily from DBLε5 subtypes also had similar expression profiles to the DBLα segment clusters. Furthermore, the clusters from region 6 of the DBLε domain often appear in conjunction with homology blocks 126 and 142, suggesting they are identifying similar domains. The DBLε5 subtype has only been described in *var1*.

A striking difference in expression profile was observed between the moderately high levels of expression in both uncomplicated and severe malaria samples of the grouping of clusters from NTSA, DBLα1, CIDRα1, and the DBLε region 6 and 8 compared to the markedly lower levels of expression in the uncomplicated samples of all other segment clusters that were up-regulated in the severe samples (Fig 9). This is consistent with the presence of NTSA and DBLα1 on all group A *var* genes and therefore their expression in both uncomplicated and severe malaria. Similarly, *var1* is ubiquitously expressed by laboratory isolates and is not subject to the same program of gene regulation as other *var* genes, and so might be expected to be expressed in both uncomplicated and severe malaria. However, these data also suggest that expression of CIDRα1 that can bind EPCR does not distinguish between severe and uncomplicated malaria as well as other *var* regions, which presumably mediate adhesion to other receptors.

The relationships between the segments, homology blocks, domains, and transcripts are illustrated in Fig 6. The Clusters from region 2 of DBLγ and regions 12 and 15 of DBLζ contained 15 transcripts, including 13 from the domain clusters DBLγ3.s.1 and DBLζ4.s.1, suggesting that we have identified similar disease-associated sequences at both the domain and segment level. Five of the transcripts included region 2 of DBLγ and both or either of regions 12 and 15 of DBLζ and 5 of the transcripts included regions 4 of CIDRγ and/or region 11 of the DBLδ. DBLδ-CIDRγ tandem domains invariably precede the DBLγ-DBLζ tandem domains of DC9. Seven of the 11 transcripts containing regions 12 and 15 of the DBLζ domain class also contained published homology block 582 [14], indicating they may be detecting similar signals (S5 Data).

Significant clusters from regions 1 and 3 of the DBLε domain class often appear in both of the previously identified domain clusters DBLε2.s.1 and DBLε9.s.1 as well as a number of other DBLε9 domain sequences. As the segment clusters collapse, 2 previous domain clusters along with a number of other sequences; this indicates that these segments may have better captured the sequence elements associated with severe disease. Due to the high diversity of the DBLε domain class at the domain level, it is hard to accurately define which sequences are associated with severe disease, and consequently this highlights the virtue of investigating these sequences at multiple resolutions.

Eight out of 11 clustered DBLβ region 4 sequences are also part of the DBLβ12 domain class. In 4 occurrences, it appears in a transcript that includes DC8. Two of the 10 DC4 transcripts clustered by Corset also contained DBLβ region 4 sequences that were part of DBLβ3. Therefore, the up-regulated DBLβ region 4 sequence collapses 2 DBLβ subtypes that are independently associated with severe malaria and implicated in different adhesion phenotypes. Five of the 13 transcripts from the cluster containing DBLβ region 8 also contained the DC4-like homology block 141, whilst 2 of the 8 transcripts from the cluster containing region 5 of DBLβ also contain the non–DC4-like DBLβ3.s.1 domain.

Transcripts from clusters of regions 5 and 7 of the DBLγ domain class don’t appear with other segments significantly associated with severe malaria, with the exception of 2 region 7 segments that appear in transcripts that include DC6. These segments may represent signal lost in the analysis of larger sequence elements.

To investigate the utility of using the different feature levels to differentiate severe and nonsevere disease, we fit a logistic regression model with lasso regularisation. A model was generated for each level of the *var* gene analysis (transcript, domain, and segment) using the features found to be up-regulated in severe disease. We made use of crossvalidation to determine the optimal lambda value for the regularisation and to give an indication of how well the features distinguish severe and nonsevere disease. Overall, the segment level provided the best discrimination, with misclassification error of 9.76% and 12.20% for the homology block and segment clusters, respectively. The misclassification for the domain-level analysis was 21.95% when using either the Rask et al. domains or the hierarchically clustered domains as features. Notably, by making use of the domains defined using the novel hierarchical approach, fewer features were required to achieve a similar classification accuracy. Distinguishing between phenotypes using a smaller number of features is important when investigating possible targets for vaccines. The transcript-level features provided the least discrimination, giving misclassification errors of 31.71% and 43.90% for the combined assembly transcripts and transcript clusters, respectively. It should be noted that these classification rates cannot be generalised to new samples because the cross validation was used to determine the lambda value as well as the misclassification rates. The code for this regression analysis is available in the Github repository.

The relationship between the segments, domains, and transcripts discussed is available in S5 Data and Fig 6. Tree diagrams, like those produced for the domains, are available for each significant segment cluster in the Github repository.

## Discussion

Transcriptional profiling of parasites isolated from patients with severe malaria indicated a shift towards a less glycolytic phenotype. Previous studies have also reported decreases in glycolytic transcripts in some clinical isolates [6], including those from patients with higher temperatures [8] as well as in parasites cultivated in vitro at a high density that inhibits subsequent growth [103]. Down-regulation of genes encoding key enzymes in folate and pyrimidine biosynthesis is also consistent with decreased nucleotide production and reduced parasite growth. The down-regulation of genes involved in histone methylation was similar to deregulation of genes involved in chromatin and RNA biology that was observed in clinical isolates from patients with an elevated surrogate measure of parasitaemia [8].

Our data suggest that parasites causing severe malaria have a more metabolically quiescent phenotype than parasites causing uncomplicated malaria. It remains to be determined whether parasites with the severe malaria transcriptional profile are more resilient and therefore able to cause severe malaria, or whether the host environment in either severe malaria or uncomplicated malaria could have selected or elicited the differing transcriptional profiles. Modulation of parasite growth in response to host environment might be consistent with previous reports of *P*. *falciparum* density sensing in malaria [103–105] and protracted maturation of *P*. *berghei* and *P*. *yoelli* in response to an acute host immune response [106]. In the latter study, more mature, circulating *P*. *berghei* and *P*. *yoelli* were detected in semi-immune than naive mice. This was consistent with our observation that the circulating parasites were older in the uncomplicated than the severe malaria patients because we previously showed that the uncomplicated malaria patients had more immunity to PfEMP1 than the severe malaria patients [88].

The parasites causing severe malaria had also down-regulated genes involved in PfEMP1 surface expression. This differed from the reported increased expression of genes encoding exported proteins involved in PfEMP1 surface expression in severe malaria from a posthoc comparison [107] of separately published transcriptomes of parasites causing severe [7] and uncomplicated [58] malaria. This difference probably relates to the difficulty of posthoc inference of differential gene expression when the compared samples are from different populations and different studies and were analysed using different microarrays. None of the 87 genes identified in up-regulated gene sets by Pelle et al. were up-regulated in severe malaria in the current study; however, 17 of these genes were down-regulated, including skeleton-binding protein 1, which was the only gene directly involved in PfEMP1 surface expression identified by Pelle et al. We previously showed that the severe malaria patients in the current study had antibodies to PfEMP1 that were generally present at lower levels and that recognised fewer PfEMP1s than the antibodies from the uncomplicated malaria patients [88]. This suggests that humoral immunity to PfEMP1 did not select for decreased PfEMP1 surface expression in the parasites causing severe malaria. However, loss or decrease of many of the proteins involved in PfEMP1 surface expression causes decreased cytoadherence [76,108,109], so the parasites infecting patients with severe malaria at the time of sampling might have had a decreased cytoadherent capacity.

The unique *var* transcriptional profile we describe in severe malaria recapitulates all of the previously described associations as well as uncovering multiple, novel sequence associations. These findings are remarkable considering that all of the associations that have been observed previously in children with severe malaria from multiple sites across Africa were found in 23 adults with severe malaria from Papua. This suggests that the same conserved *var* genes are associated with severe disease in nonimmune individuals regardless of geography or patients’ age. Furthermore, a consistent pattern of expression of restricted subsets of *var* genes, domains, and/or segments was observed despite heterogeneous presentations of severe disease. Similarly, the severe malaria non-*var* transcriptome clusters also did not segregate by specific severe malaria syndromes. These observations suggest that common mechanisms of disease may cause the varied syndromes of severe malaria. This could have therapeutic implications, although the analyses should be confirmed with larger sample sets.

These findings emphasise the strength of the association between severe malaria and DC8, DC4, DC6 CIDRα1, DBLβ3, and DBLβ12 sequences, which were each shown to be up-regulated in multiple analyses of the de novo *var* assemblies. However, they also uncover significant, novel associations with other *var* sequences at the transcript, domain, and segment level. Some of these were found at multiple levels of analysis, e.g., DC11, CIDRα2.6-DBLβ8, DBLε3, DBLγ3, DBLζ4, and DBLε2/9, and in the individual domain analysis, the latter 4 domains were expressed at least as highly in severe malaria as the EPCR-binding CIDRα1 sequences. We cannot exclude the possibility that some of these domains were present on the same PfEMP1 as a CIDRα1 sequence; however, CIDRα1 was not present on the significantly up-regulated transcripts that carried these other domains in either the combined assembly or the corset analysis of the individual isolate *var* assemblies.

We developed a novel analytical approach testing sequences for associations with disease at multiple levels of sequence homology. This revealed domain subtypes that were strongly associated with disease, including a highly conserved CIDRβ1 subtype and a DBLδ1 subtype that clustered in the same patients. The diversity of the parent CIDRβ1 and DBLδ1 subtypes prevented detection of an association using the established subtype classifications. Finally, we revealed striking associations between smaller *var* sequence segments and severe disease again by testing for associations at multiple levels of sequence identity. These small segments were limited in number, and many of the findings recapitulated our domain analysis. Some of these segments collapsed multiple domain subtypes, e.g., DBLβ_block4 collapsed DBLβ3 and DBLβ12, raising the possibility that a single segment may elicit cross-reactive immunity against different domain subtypes that are independently associated with severe disease. These segments may help identify critical, fine-scale details of the *var* sequences expressed by parasites that cause disease and may be of great utility in designing vaccines for severe malaria. The association of these sequences with severe malaria should be validated in other populations from across the world and the encoded proteins tested for adhesion phenotype and for seroreactivity consistent with protection from severe malaria.

## Materials and methods

### Ethics statement

Written, informed consent was provided by all participants. The study was approved in Indonesia by the Eijkman Institute Research Ethics Commission (project number 46), in Australia by the Melbourne Health Human Research Ethics Committee (project number 2010.284) and Human Research Ethics Committee of the NT Department of Health & Families and Menzies School of Health Research, Darwin, Australia (HREC 2010–1396).

### Data sets

The data sets generated and/or analysed during the current study are available in the Arrayexpress repository accession: E-MTAB-5860 (sequenced libraries for each sample) and the ENA repository accession: PRJEB20632 (de novo *var* gene assemblies for combined and individual samples).

### Sample collection

Venous samples were collected from patients with severe (*n* = 23) and uncomplicated (*n* = 21) malaria attending a healthcare facility in Timika, Papua Province, Indonesia. This area has unstable malaria transmission, with estimated annual parasite incidence of 450 per 1,000 population and symptomatic illness in all ages [110]. Severe malaria was defined as peripheral parasitaemia with at least one modified World Health Organization (WHO) criterion of severity [111]. All of the 23 patients with severe malaria had parasitemias greater than 1,000 per μL, which is a previously derived threshold that predicts clinical disease in northern Papua [49]. Therefore, incidental parasitaemia is unlikely in these severe malaria patients.

### RNA extraction and RNAseq

White blood cells were depleted from the blood by retention on CF11 cellulose (Whatman-no longer available) using a modification of a previously described protocol [112] (S1 Text Supplementary methods). RNA was extracted from erythrocytes in TRIzol using a modified RNeasy mini (QIAGEN, Hilden, Germany) protocol (S1 Text Supplementary methods). Purified RNA 1 to 3 μg was depleted of Hb mRNA using the Globinclear human Hb RNA depletion kit (Ambion, Thermo Fisher Scientific, Waltham, MA) and a modified protocol (S1 Text supplementary methods).

### Sequencing

mRNA was oligo dT purified from the total RNA using the NEBNext Poly(A) mRNA magnetic isolation module (New England Biolabs, Ipswich, MA) and mRNA fragmented, reverse transcribed, and used for library synthesis using the NEBnext ultra directional RNA library prep kit for Illumina (New England Biolabs) as per the manufacturer’s instructions but with modifications (S1 Text supplementary methods), including a high AT tolerant PCR amplification [113]. Libraries were 100 bp paired end sequenced on a 2500-HT Hiseq (Illumina, San Diego, CA) using RapidRun chemistry (Illumina).

### De novo assembly of *var* genes

Briefly, de novo assembly of *var* genes was performed by running the SoapDeNovo-Trans [114] and Cap3 [115] pipeline described in [53] (Fig C in S2 Fig). Non-*var* reads were first filtered out by removing reads that aligned to the *H*. *sapiens*, *P*. *vivax*, and non-*var P*. *falciparum* reference genomes. The resulting contigs were filtered for contaminants and translated into the correct reading frame. A more thorough description of the assembly methods is available in S1 Text. Additionally, the code used to run the pipeline is available on Github at https://github.com/PapenfussLab/assemble_var.

### All gene expression analysis

Reads were first aligned to the *H*. *sapiens* and *P*. *falciparum* reference genomes using Subread-align v1.4.6 [97] with parameters -u -H. FeatureCounts v1.20.2 [116] was used to obtain read counts for each gene. To account for parasite life cycle, each sample is estimated as a mixture of 6 parasite life cycle stages from [57], excluding the ookinete stage. We aimed to choose the proportions **π** for each sample to minimise

$$\sum_{i=1}^{N}(g_{i,sample}-\sum_{s\in S}{\pi_{s}g_{i,s})}^{2}$$

subject to the constraints

$$\sum_{s\in S}\pi_{s}=1$$

And

$$\pi_{s}\geq 0$$

such that g_*i,s*_ represents the expression of the ith gene in stage s of the [57] data.

Three factors of unwanted variation were estimated using the RUV4 function from the R package ruv v0.9.6 [117] using the 1,009 genes with the lowest *p*-values from [118] as controls. The choice of control genes was compared to using the least differentially expressed genes of [8], which was found to give similar results. Finally, the gene counts along with the estimated ring-stage factor, and 3 factors of unwanted variation estimated by RUV4 were fed into the Limma/Voom [55,56] differential analysis pipeline. For a detailed outline of the specific commands run in the all-gene analysis, see S1 Text and rmarkdown S1 Text available in the Github repository https://github.com/gtonkinhill/falciparum_transcriptome_manuscript.

### *var* gene expression analysis

For all levels of *var* expression analysis, library size was normalised using the median ratio method [119] in the default DESeq2 pipeline [86].

### Transcript level

Differential expression analysis at the *var* transcript level was performed using 2 distinct approaches. The first made use of the separately assembled transcripts by first aligning reads to the transcripts allowing for multiple mapping using Bowtie v0.12.9 [120]. The transcripts were then clustered using Corset v1.03 [89]. The resulting cluster read counts were analysed using the default DESeq2 pipeline [86]. An alternative strategy made use of the combined assembly transcripts. Reads were aligned and transcript level counts obtained using Subread and Featurecounts, respectively, before analysing differential expression using DESeq2. Rmarkdown texts S2 and S3 in the Github repository provide a more thorough description of this analysis along with the code https://github.com/gtonkinhill/falciparum_transcriptome_manuscript.

### Type/domain level

HMMER3’s hmmsearch v3.1b1 [91] was used to search the NTS, DBL, and CIDR domain models of [14] against the assembled transcripts from each sample. The most significant domain was annotated first and then successively less significant domains, with the requirement that 2 domains do not overlap. An E-value threshold of 1e-8 was chosen to minimise spurious annotations.

FeatureCounts was used to allocate reads to domains using a SAF file built from the HMMER3 annotations. The resulting counts were then aggregated using the previous domain classification of [14] as well as a novel hierarchical approach. The annotated domains were hierarchically clustered using USEARCH [93] by first clustering by length and then by successively lower identity thresholds. The read counts for each domain are then aggregated up this hierarchical tree, and the default DESeq2 pipeline was used to identify differentially expressed nodes. After multiple testing correction [94], we iteratively reject the null hypothesis (*p <* 0.05) of the most significant node in the hierarchy before removing its ancestor and children nodes. This ensures that we select the most significant grouping of domains from which to form clusters. DESeq2 was also run on the domains aggregated using the previous classification.

### Segment level

#### Homology block analysis

In a similar fashion to the domain-level analysis, 613 of the possible 628 homology blocks of [14] were aligned to the separate assembly transcripts using a bit score cutoff of 9.97 as is described in [14]. Read counts were again aggregated for each homology block before the default DESeq2 pipeline was used to analyse differential expression.

#### Identification of novel, differentially expressed segments

To identify novel segments, *var* domains were initially identified as in the domain section. Major domain classes were aligned using Gismo v2.0 [100]. The resulting alignments were then segmented into regions of high and low occupancy in an approach comparable to [98]. If 7 or more consecutive columns within an alignment had an occupancy greater than 95%, these columns were considered a conserved region. Terminal gaps were not counted in the occupancy calculations. The columns in between these conserved regions were considered variable regions. Each domain sequence was then split into segments based on the regions, and the segments were clustered hierarchically within each region using CD-HIT v4.6 [102]. CD-HIT was chosen because it takes the terminal ends into account when calculating pairwise identity, making it more appropriate for the short segments being considered here. Finally, as was done at the domain level, differential expression analysis was performed on each node of the hierarchy, and the most significant nodes were successively chosen.

A more thorough description of the methods for identifying the differentially expressed domain and homology block segments is given in S1 Text as well as in the rmarkdown texts S4–S6 available in the Github repository.

#### Untargeted LC-MS profiling

Plasma samples (5 μL) were extracted with 80% acetonitrile containing 1 μM of 13C15N aspartate (internal standard) and LC-MS analyses performed as described previously [121] (S1 Text supplementary methods). Data were converted to mzXML and analysed using the MAVEN software package [122]. Comparison between severe and uncomplicated samples was performed using a Benjamini Hochberg–corrected *t* test with significance set at *p* < 0.01, and significant features were searched in the METLIN database for putative identification.

## Supporting information

S1 Fig RNA quality and summary statistics on raw RNAseq data. (A) RQI values from BioRad Experion automated RNA electrophoresis system. RQI values can range from 1 (low) to 10 (high); 28S/18S rRNA ratios are also provided; N/A indicates samples for which values could not be interpolated because the molecular weight standard ladder failed, though RNA quality for these samples could still be assessed visually from the electophoretograms. The rRNA profile differs from the typical 2 peaks because it is a mixture of *H*. *sapiens* and *P*. *falciparum* 28S and 18S rRNAs; the *P*. *falciparum* rRNAs migrate as the 2 inner peaks. (B) Number of fragments (read pairs) assigned to genes of the *P*. *falciparum* reference genome. A sample was required to have at least 1 million fragments to be included in the rest of the analysis. (C) Summary diagram of the approaches taken to analyse the RNAseq data. RNAseq, RNA sequencing; RQI, RNA Quality Index.
(PDF)

S2 Fig De novo *var* gene assembly. (A) Assembled transcripts greater than 500 nt in length were aligned using BLAST to the sequence database from [14]. Multiple alignments were allowed. The resulting alignments are plotted by reference sequence, including the percentage identity of the alignment. The alignment is coloured in bold, whilst sequence not aligned to the reference is translucent. The vertical black line indicates the end of the reference sequences. Fig A in S2 Fig indicates the alignment of the simpler *P*. *falciparum* ItG subclone E8B. (B) Similar to panel A; however, the ItG subclone CS2—in which there has been a recombination between IT4var04 and IT4var08 var genes—is displayed. (C) A flow diagram of the final assembly pipeline used to generate var gene transcripts from RNAseq data. RNAseq, RNA sequencing.
(PDF)

S3 Fig Differential expression analysis of all genes. Average linkage heatmap of the expression levels of the 358 deregulated genes from the differential analysis results for all genes with sufficient coverage. Two clusters of severe malaria transcriptomes that differ by expression profile are indicated as ‘S1’ and ‘S2’. Normalised CPM data are available in S1 Data. CPM, counts per million mapped reads.
(PDF)

S4 Fig Differential expression analysis of *var* gene features. (A) Comparison of expression levels of NTSA and NTSB segments for severe and nonsevere phenotypes. NTSA was found to be significantly up-regulated in severe disease. (B) PCA plot of the read counts associated with transcripts from the combined sample assembly. The severe samples are clustered more tightly than the nonsevere. This is consistent with a more conserved set of features that describe severe disease. The less tightly clustered nonsevere samples are consistent with the difficulty in obtaining complete immunity to malaria. Raw read counts used for S4 Fig are available in S9 Data. NTSA, N-terminal sequence A; NTSB, N-terminal sequence B; PCA, Principal Component Analysis.
(PDF)

S5 Fig PCA plots of read counts for different identity levels of the *var* domain hierarchical clustering. These plots are similar to the PCA plot in Fig B in S4 Fig, indicating that we are still capturing similar differences between severe and nonsevere cases using these domain clusters. Raw read counts transformed for S5 Fig are available in S9 Data. PCA, Principal Component Analysis.
(PDF)

S6 Fig Transformed data, scatter plots, and Spearman correlations for *var* domain expression measured by Q-RT-PCR (2^-ΔCp^) and RNAseq (RPKM). Red dots are severe malaria samples SFC12, SFC14, SFC15, SFC17, SFC19, SFC22, SFM1, SFM3, SFU2, and SXC2; blue dots are uncomplicated malaria samples IFM049, IFM047, IFM050, IFM054, IFM12, IFM27, IFM53, and IFM56; black dots are samples that were excluded from the correlation analysis because they had high RPKM values, but the relevant transcripts lacked the Q-RT-PCR primer binding sites (670_X0.6—DBLε2, 348_X0.5—DBLγ3, 345_X0.5_DBLγ13.ns.1), or the PCR product had a different dissociation curve to all other products amplified with those primers (226_X0.6—DBLβ12). DBL, Duffy binding-like; Q-RT-PCR, quantitative reverse transcription PCR; RNAseq, RNA sequencing; RPKM, Reads Per Kilobase of transcript per Million mapped reads.
(PDF)

S7 Fig Expression levels of homology blocks as defined in [14] found to be up-regulated in severe disease. (A) Heatmap of expression level with patients’ samples and homology blocks grouped using complete linkage hierarchical clustering. (B) PCA plot of read counts annotated to homology blocks identified in transcripts from the separate sample assembly. This plot indicates that homology blocks do not separate severe disease as distinctly as the domain clusters. Raw read counts that were transformed for these figures are provided in S11 Data.
(PDF)

S8 Fig Multiple sequence alignments used to define *var* domain segments. Logos of major domain classes produced using Skylign [101] on the output of Gismo [100]. Hierarchical clustering of segments was used for differential expression analysis.
(PDF)

S1 Text Supplementary methods. (DOCX)

S2 Text Phylogenetic analysis of *var* domains with known DC4 and DC8 DBLβ3, DBLβ12, DBLδ1, and CIDRβ1 sequences. CIDR, cysteine-rich interdomain region; DBL, Duffy binding-like; DC, domain cassette.
(PDF)

S1 Table Raw read counts by sample for *H*. *sapiens* and *P*. *falciparum*. %Pf, percentage of reads that mapped to *P*. *falciparum*. (PDF)

S2 Table Comparison of approaches for *var* gene de novo assembly. EIC and ECS are separate subcultures of the E8B clone of the ItG isolate; CS2 is a subclone of E8B.
(PDF)

S3 Table De novo–assembled *var* transcript statistics by sample. (PDF)

S4 Table Deregulation in severe malaria of *P*. *falciparum* gene sets previously reported to be deregulated in vivo. FDR, false discovery rate; Prp, proportion.
(DOCX)

S1 Data All *P*. *falciparum* genes differentially expressed in severe malaria. Chr, chromosome; CI, confidence interval; logFC, log fold-change.
(XLSX)

S2 Data *P*. *falciparum* gene expression analysis, proportion by life cycle stage and read counts normalised for library size and/or for variations in life cycle staging and other batch effects. (XLSX)

S3 Data GO and KEGG classifications that were enriched in genes that were differentially expressed in severe malaria. GO, Gene Oncology; KEGG, Kyoto Encyclopedia of Genes and Genomes.
(XLSX)

S4 Data Mz and Rt of metabolites enriched or depleted from plasmas of severe malaria patients. Av, average; Mz, mass charge; Rt, retention time; SD, standard deviation.
(XLSX)

S5 Data Assembled *var* transcripts from the separate assemblies. Includes the translated protein sequences for all the transcripts as well as the annotated domain, domain clusters, homology blocks, and segment clusters. For each of the separate assembly transcripts, its closest BLAST hit in the combined assembly is included as well as the cluster to which it belongs from the Corset analysis. The results of the differential expression analyses are also summarised. HB, homology block; LFC, log fold-change; Orf, open reading frame.
(XLSX)

S6 Data Assembled *var* transcripts from the combined assembly. Includes the translated protein sequences for all the transcripts as well as the annotated domain and homology blocks. The results of the differential expression analyses are also summarised. Raw read counts used for Fig 4A heatmap and RPKM used for Fig 4B boxplot are also included. HB, homology block; LFC, log fold-change; Orf, open reading frame; RPKM, Reads Per Kilobase of transcript per Million mapped reads.
(XLSX)

S7 Data *Var* transcripts from the separate assemblies clustered by CORSET. The results of the differential expression analyses are also summarised.
(XLSX)

S8 Data *Var* domains classified by HMMER [14] that were significantly deregulated in severe malaria. The results of the differential expression analyses are also summarised.
(XLSX)

S9 Data *Var* domain clusters at different percent identities that were significantly deregulated in severe malaria. The domains from the separate assembly *var* transcripts that are present in each cluster are identified. The results of the differential expression analyses are also summarised. Transformed read counts at 50% identity, RPKM for all deregulated domains, and raw read counts for each domain from the separate de novo assemblies are also provided. RPKM, Reads Per Kilobase of transcript per Million mapped reads.
(XLSX)

S10 Data Thirty-four *var* domain sequences from the separate sample assemblies that were cloned and sanger sequenced from patient isolate gDNA. gDNA, genomic DNA.
(XLSX)

S11 Data *Var* sequence homology blocks [14] that were significantly deregulated in severe malaria. Differential expression analyses and raw read count data are provided.
(XLSX)

S12 Data *Var* sequence segment clusters that were significantly deregulated in severe malaria. The sequence blocks within domains from the separate assembly *var* transcripts that are present in each cluster are identified. The results of the differential expression analyses and the raw read counts that were transformed for Fig 9 are also provided.
(XLSX)

## Abbbreviations

acetyl-CoA: acetyl coenzyme A
adj-p: adjusted *p*
ATCase: aspartate carbamoyltransferase
ATS: acidic terminal sequence
BCKDH: branched chain ketoacid dehydrogenase
BLAST: basic local alignment search tool
CCT: choline-phosphate cytidylyltransferase
CIDR: cysteine-rich interdomain region
CYP52: cytochrome P450 52
CyRPA: cysteine-rich protective antigen
DBL: Duffy binding-like
DC: domain cassette
DHPS: dihydropteroate synthetase
dNTP: deoxyribonucleoside triphosphate
ENA: European Nucleotide Archive
EPCR: endothelial cell protein C receptor
ER: endoplasmic reticulum
FKBP: FK506 binding protein
gDNA: genomic DNA
GO: Gene Ontology
GTP: guanosine triphosphate
GTPase: guanosine triphosphatase
Hb: haemoglobin
HIP: HSP70 interacting protein
HMM: Hidden Markov Model
HOP: hsp70/hsp90 organising protein
HRP2: Histidine Rich Protein 2
ICAM1: intercellular adhesion molecule 1
IE: infected erythrocyte
IQR: interquartile range
KEGG: Kyoto Encyclopedia of Genes and Genomes
LC-MS: liquid chromatography–mass spectrometry
logFC: log fold-change
LPD1: dihydrolipoyl dehydrogenase
LyMP: lysine-rich, membrane-associated PHISTb protein
NDK: nucleoside diphosphate kinase
NDP: ribonucleoside diphosphate
NTP: ribonucleoside triphosphate
NO: nitric oxide
NTS: N-terminal sequence
PC: principal component
PCA: Principal Component Analysis
PfEMP1: *Plasmodium falciparum* Erythrocyte Membrane Protein 1
PfPRMT1: protein arginine N-methyltransferase 1
PfRH5: Plasmodium falciparum reticulocyte-binding protein homolog 5
qPCR: quantitative PCR
Q-RT-PCR: quantitative reverse transcription PCR
RNAseq: RNA sequencing
RPKM: Reads Per Kilobase of transcript per Million mapped reads
RQI: RNA Quality Index
RUV: Remove Unwanted Variation
SIAP-2: sporozoite invasion associated protein-2
SNARE: Soluble NSF (N-ethylmaleimide-sensitive factor) Attachment Protein Receptor
TCA: tricarboxylic acid
TM: transmembrane
TMM: trimmed mean of M values
TRAPPC5: trafficking protein particle complex subunit 5
uncompl: uncomplicated
UPS: upstream sequence
WHO: World Health Organization
