# Supplementary material for: The Plasmodium falciparum transcriptome in severe malaria reveals altered expression of genes involved in important processes including surface antigen–encoding var genes
Source: PLoS Biol. 2018 Mar 12;16(3):e2004328. doi: 10.1371/journal.pbio.2004328 (PMC5864071; doi:10.1371/journal.pbio.2004328)
Supplement: S1 Fig — (A) RQI values from BioRad Experion automated RNA electrophoresis system. RQI values can range from 1 (low) to 10 (high); 28S/18S rRNA ratios are also provided; N/A indicates samples for which values could not be interpolated because the molecular weight standard ladder failed, though RNA quality for these samples could still be assessed visually from the electophoretograms. The rRNA profile differs from the typical 2 peaks because it is a mixture of H. sapiens and P. falciparum 28S and 18S rRNAs; the P. falciparum rRNAs migrate as the 2 inner peaks. (B) Number of fragments (read pairs) assigned to genes of the P. falciparum reference genome. A sample was required to have at least 1 million fragments to be included in the rest of the analysis. (C) Summary diagram of the approaches taken to analyse the RNAseq data. RNAseq, RNA sequencing; RQI, RNA Quality Index. (PDF) [file pbio.2004328.s001.pdf]

S1 A)

Sample	Ratio 28S:18S rRNA	RNA Quality Index (RQI BioRad)
SFM 001	0.33	7.5
SFM 003	0.91	8.5
SFM 005	0.49	6
SFM 006	N/A	N/A
SFM 007	0.2	5.6
SFM 008	0.31	9.1
SFM 009	0.79	8.4
SFM 010	0.36	8.9
SFD 001	0.41	10
SXC 002	0.58	7.2
SFC 013	0.44	7.5
SFC 014	0.93	8.3
SFC 015	0.83	7.8
SFC 016	0.82	7.1
SFC 017	1.36	9.5
SFC 018	0.89	8.1
SFU 002	1.09	9.4
SFC 019	0.71	8
SFU 003	0.2	9
SFC 021	N/A	N/A
SFC 022	0.94	8.8
SFC 023	0.6	6.8
SFC 025	0.55	6.9
IFM 012	0.3	7.3
IFM 014	0.44	6.4
IFM 021	1.09	8.7
IFM 023	1.34	9.1
IFM 024	0.27	7.5
IFM 026	0.45	8.5
IFM 027	0.78	7.4
IFD 006	0.27	5.9
IFD 008	1.13	8.9
IFM 047	0.96	8.5
IFM 049	0.94	8.3
IFM 050	0.85	7.7
IFM 053	N/A	7.8
IFM 054	0.78	7.3
IFM 056	N/A	7.3
IFM 057	N/A	6.4
IFC 058	0.7	7.4
IFC 060	0.88	8
IFC 061	0.67	7.5
IFC 063	0.53	7.1
IFC 066	N/A	6.8

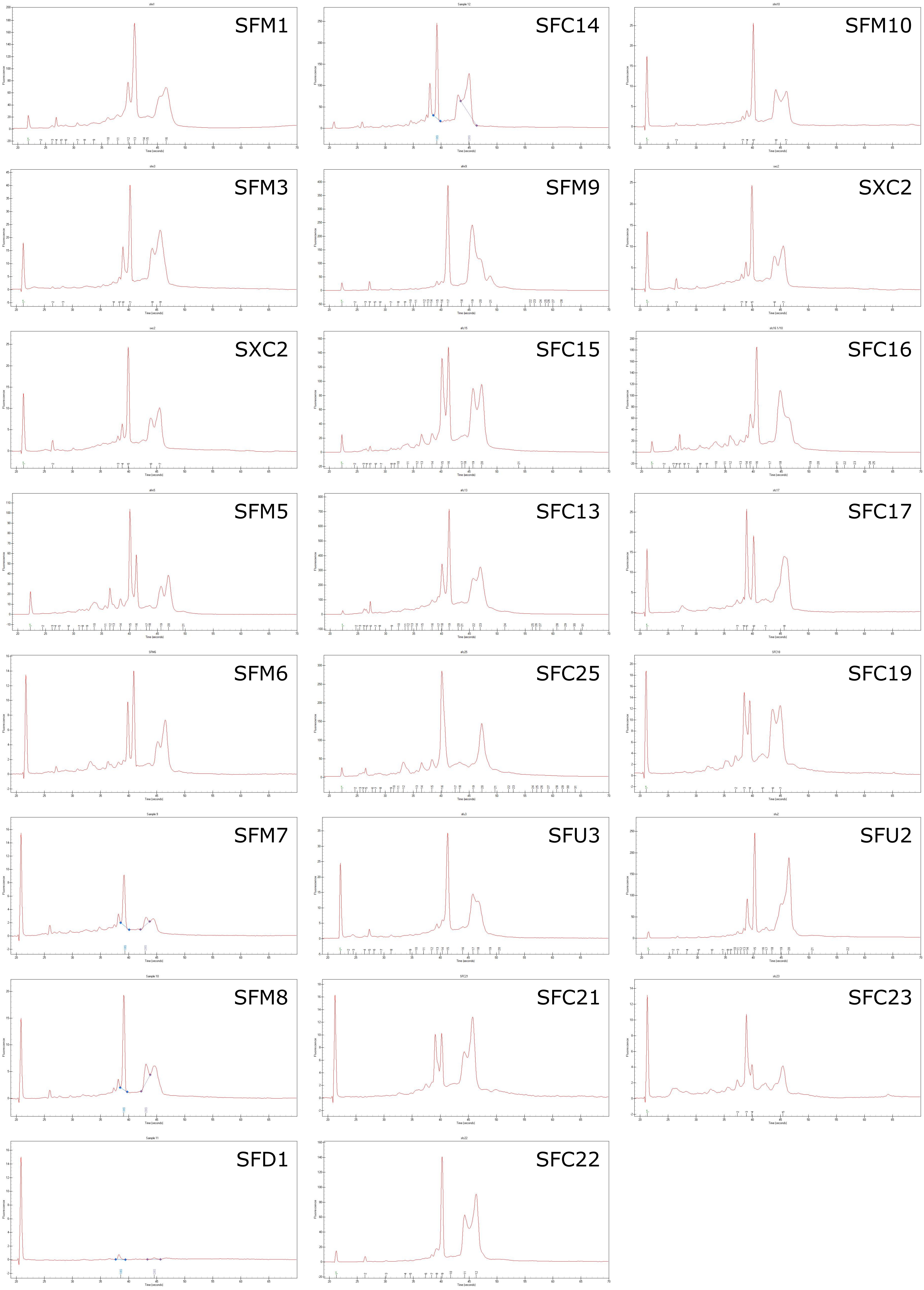
SFM1
SFC14
SFM10
SFM3
SFM9
SXC2
SXC2
SFC15
SFC16
SFM5
SFC13
SFC17
SFM6
SFC25
SFC19
SFM7
SFU3
SFU2
SFM8
SFC21
SFC23
SFD1
SFC22

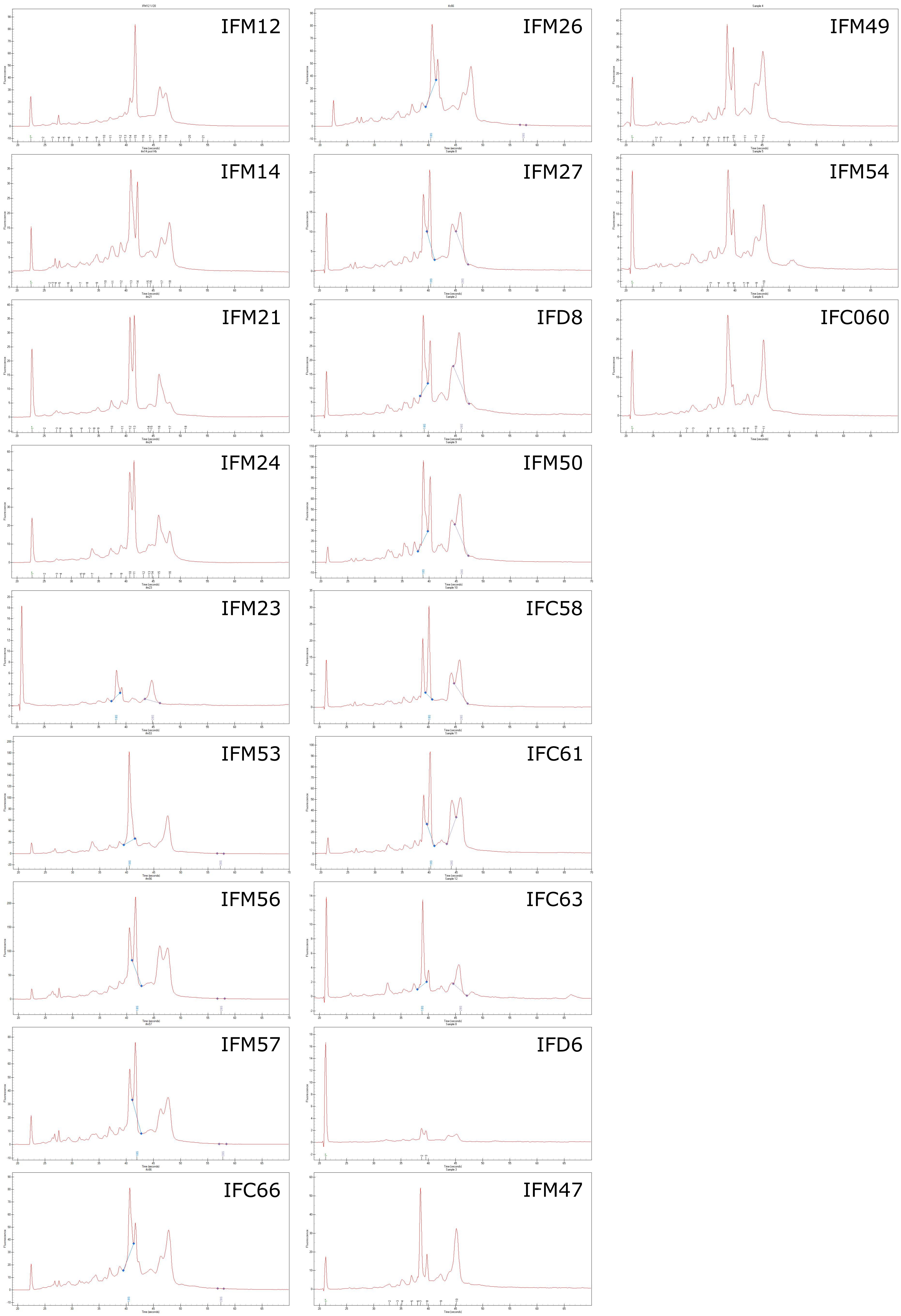
IFM12
IFM26
IFM49
IFM14
IFM27
IFM54
IFM21
IFD8
IFC060
IFM24
IFM50
IFM23
IFC58
IFM53
IFC61
IFM56
IFC63
IFM57
IFD6
IFC66
IFM47

S1 B)

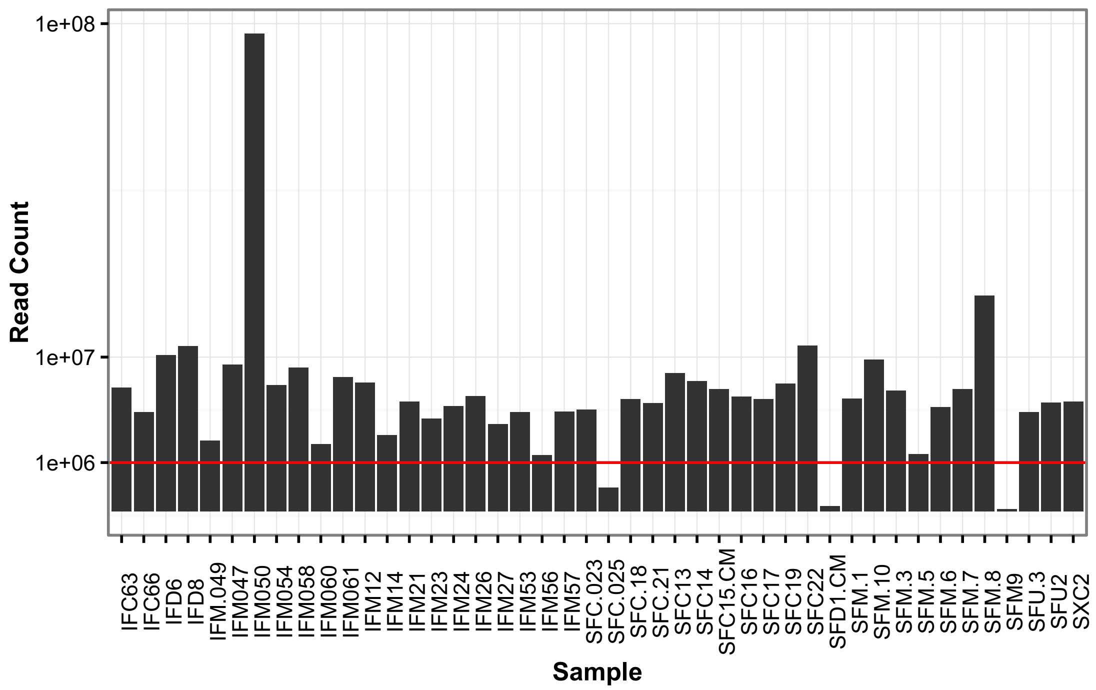
1e+08
1e+07
1e+06
Read Count
IFC63
IFC66
IFD6
IFD8
IFM.049
IFM047
IFM050
IFM054
IFM058
IFM060
IFM061
IFM12
IFM14
IFM21
IFM23
IFM24
IFM26
IFM27
IFM53
IFM56
IFM57
SFC.023
SFC.025
SFC.18
SFC.21
SFC13
SFC14
SFC15.CM
SFC16
SFC17
SFC19
SFC22
SFD1.CM
SFM.1
SFM.10
SFM.3
SFM.5
SFM.6
SFM.7
SFM.8
SFM9
SFU.3
SFU2
SXC2
Sample

S1 C)

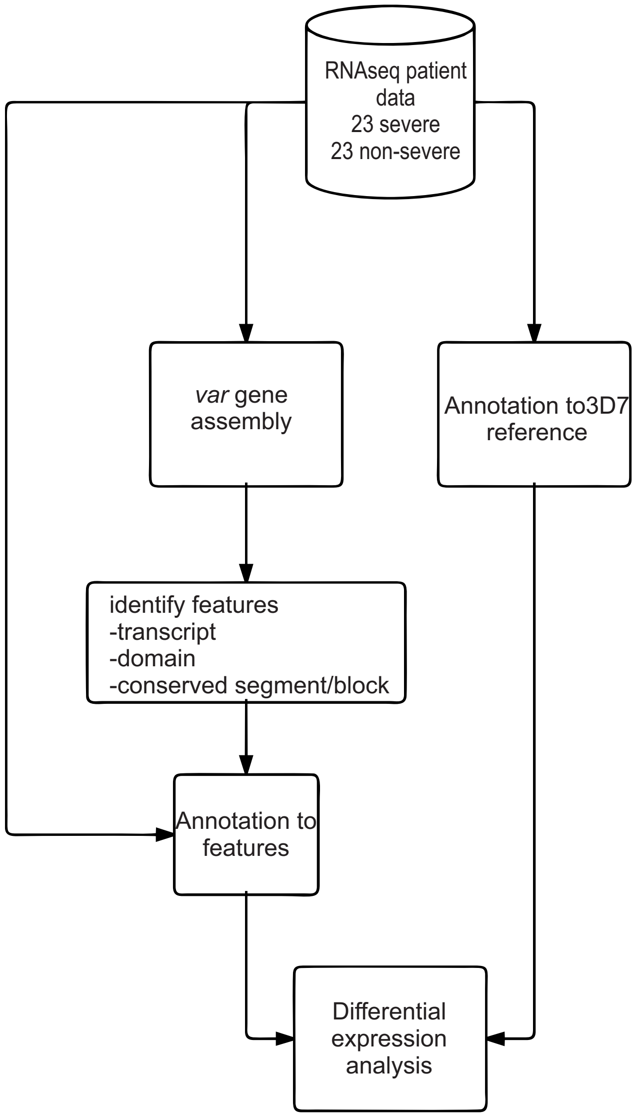
RNAseq patient data
23 severe
23 non-severe
var gene assembly
Annotation to3D7 reference
identify features
-transcript
-domain
-conserved segment/block
Annotation to features
Differential expression analysis
