# Supplementary figures and images for: The Plasmodium falciparum transcriptome in severe malaria reveals altered expression of genes involved in important processes including surface antigen–encoding var genes
Source: PLoS Biol. 2018 Mar 12;16(3):e2004328. doi: 10.1371/journal.pbio.2004328 (PMC5864071; doi:10.1371/journal.pbio.2004328)

s2a)

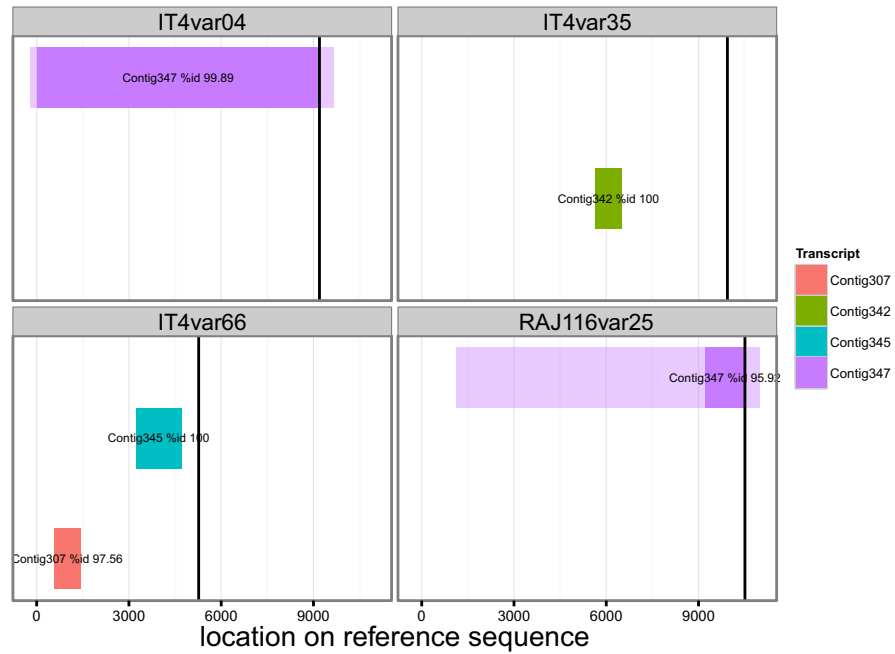

s2b)

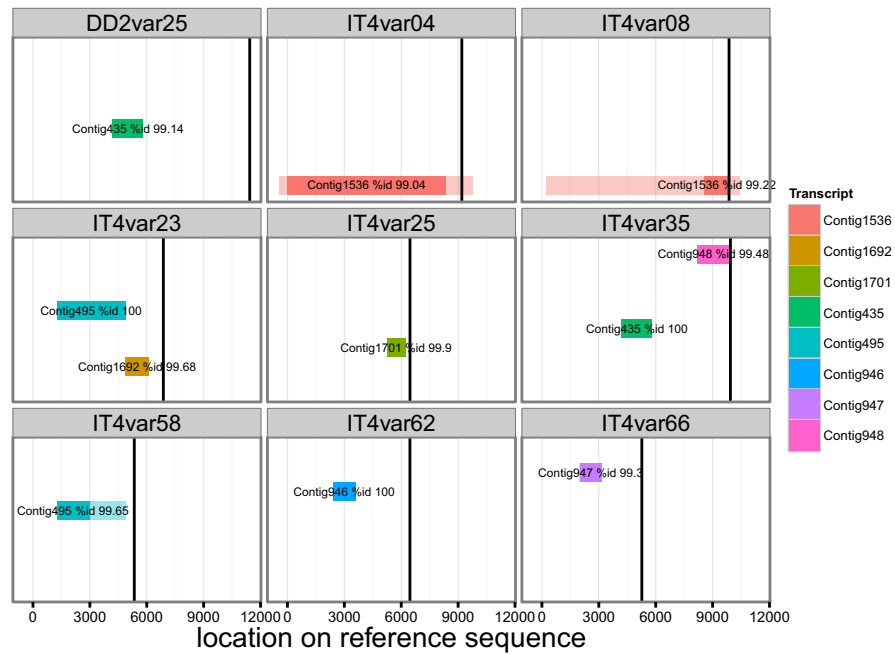

s2c)

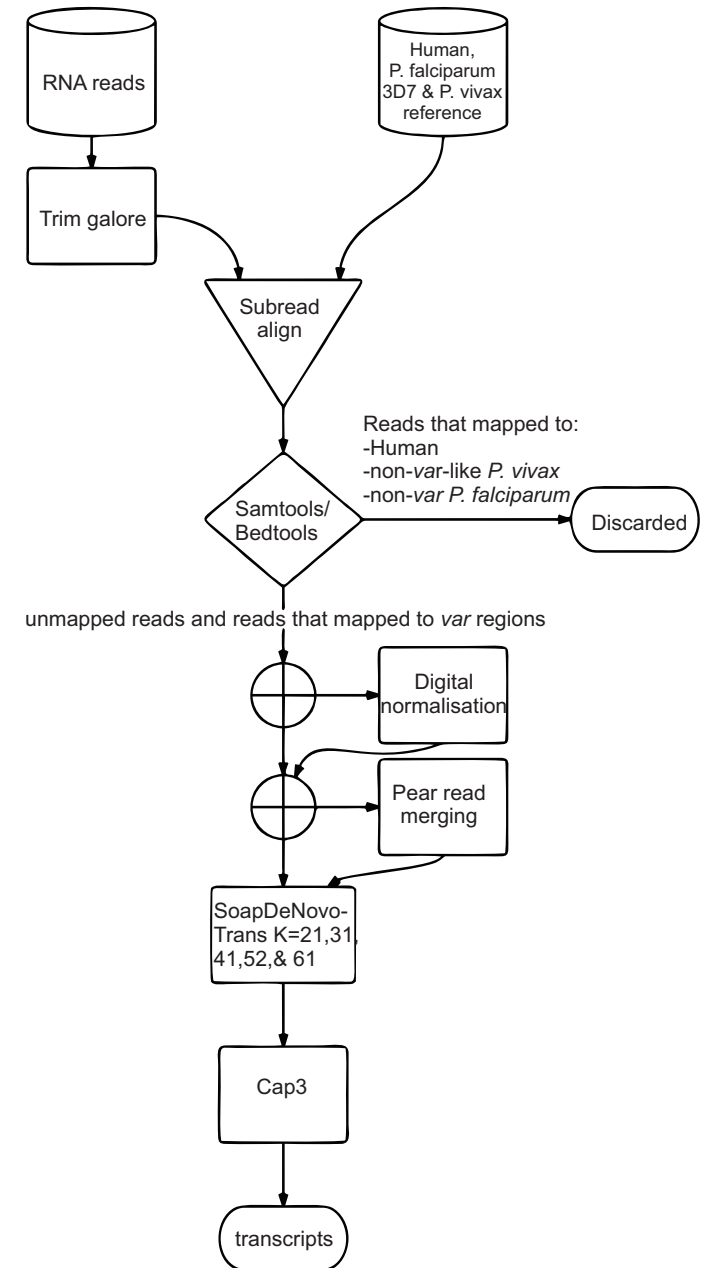

Supplement: S2 Fig — (A) Assembled transcripts greater than 500 nt in length were aligned using BLAST to the sequence database from [14]. Multiple alignments were allowed. The resulting alignments are plotted by reference sequence, including the percentage identity of the alignment. The alignment is coloured in bold, whilst sequence not aligned to the reference is translucent. The vertical black line indicates the end of the reference sequences. Fig A in S2 Fig indicates the alignment of the simpler P. falciparum ItG subclone E8B. (B) Similar to panel A; however, the ItG subclone CS2—in which there has been a recombination between IT4var04 and IT4var08 var genes—is displayed. (C) A flow diagram of the final assembly pipeline used to generate var gene transcripts from RNAseq data. RNAseq, RNA sequencing. (PDF) [file pbio.2004328.s002.pdf]

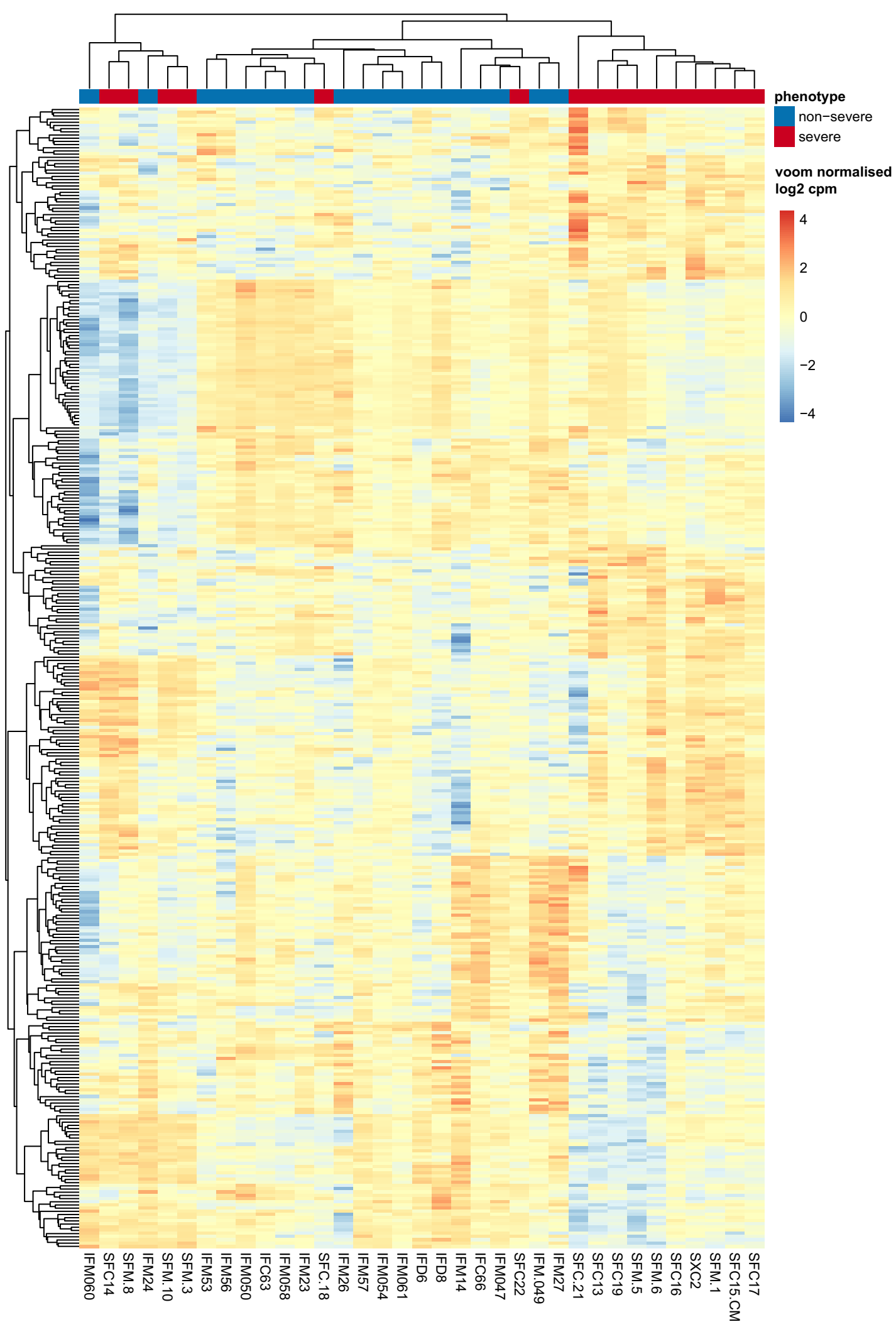

Supplement: S3 Fig — Average linkage heatmap of the expression levels of the 358 deregulated genes from the differential analysis results for all genes with sufficient coverage. Two clusters of severe malaria transcriptomes that differ by expression profile are indicated as ‘S1’ and ‘S2’. Normalised CPM data are available in S1 Data. CPM, counts per million mapped reads. (PDF) [file pbio.2004328.s003.pdf]

s4a)

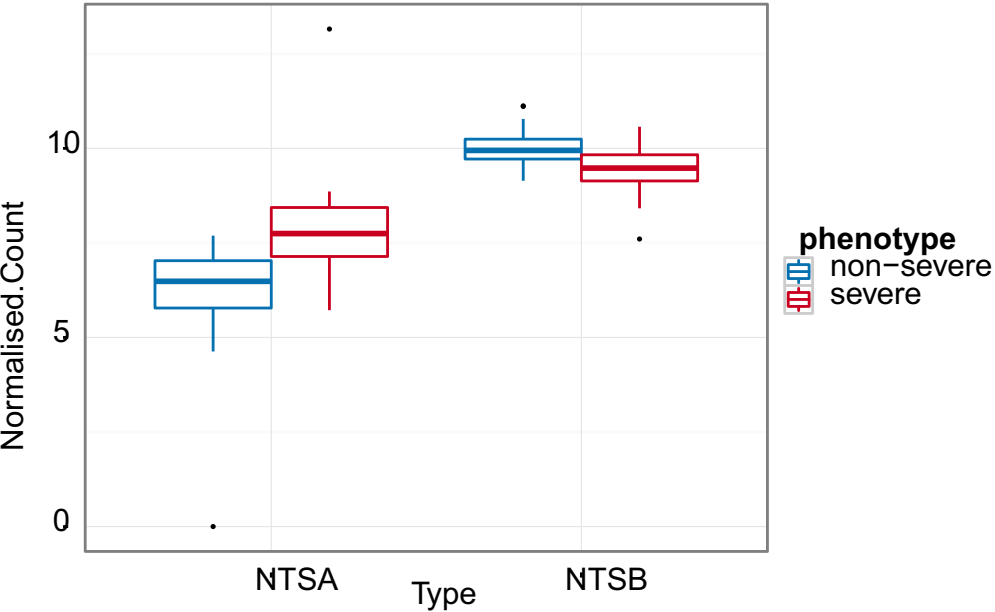

s4b)

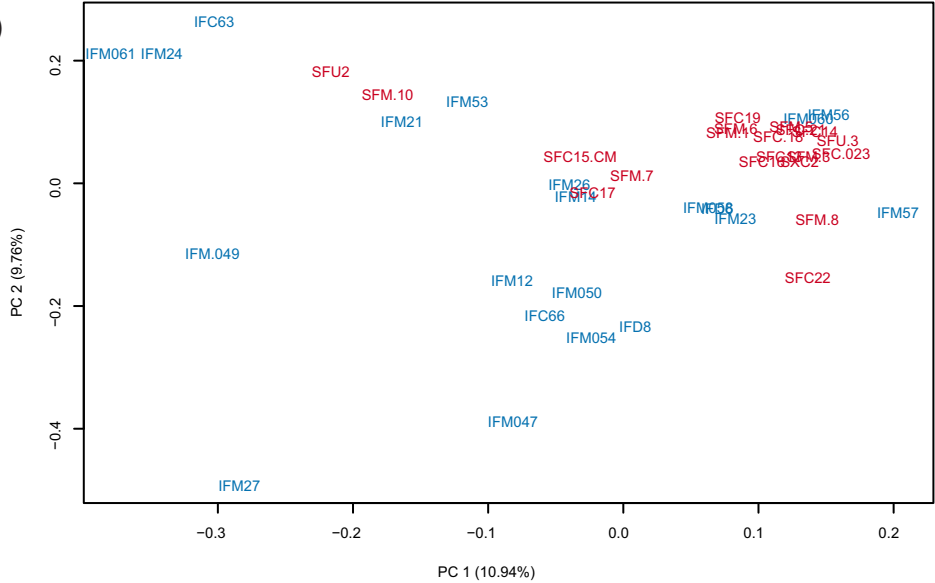

Supplement: S4 Fig — (A) Comparison of expression levels of NTSA and NTSB segments for severe and nonsevere phenotypes. NTSA was found to be significantly up-regulated in severe disease. (B) PCA plot of the read counts associated with transcripts from the combined sample assembly. The severe samples are clustered more tightly than the nonsevere. This is consistent with a more conserved set of features that describe severe disease. The less tightly clustered nonsevere samples are consistent with the difficulty in obtaining complete immunity to malaria. Raw read counts used for S4 Fig are available in S9 Data. NTSA, N-terminal sequence A; NTSB, N-terminal sequence B; PCA, Principal Component Analysis. (PDF) [file pbio.2004328.s004.pdf]

s5)

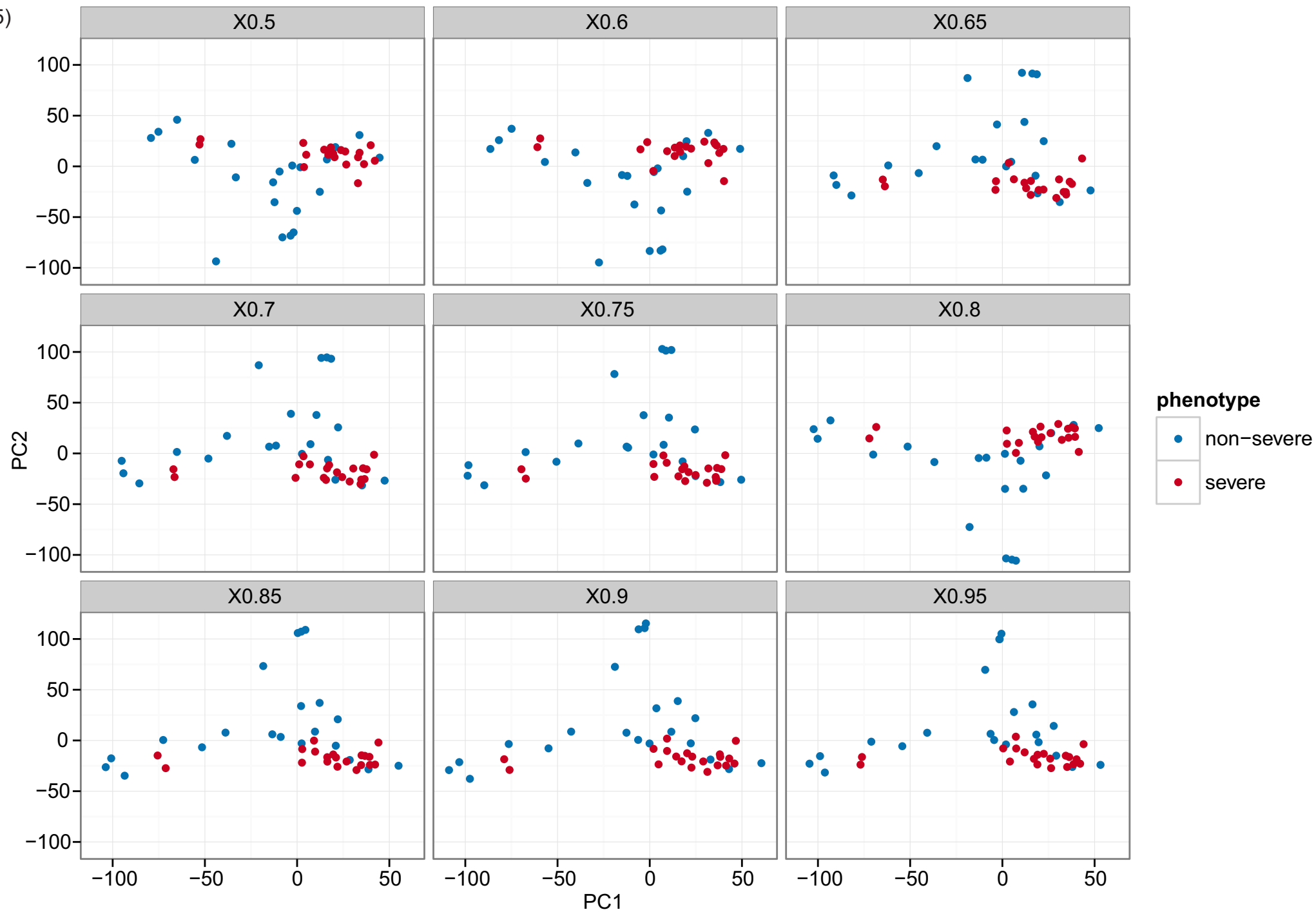

Supplement: S5 Fig — These plots are similar to the PCA plot in Fig B in S4 Fig, indicating that we are still capturing similar differences between severe and nonsevere cases using these domain clusters. Raw read counts transformed for S5 Fig are available in S9 Data. PCA, Principal Component Analysis. (PDF) [file pbio.2004328.s005.pdf]

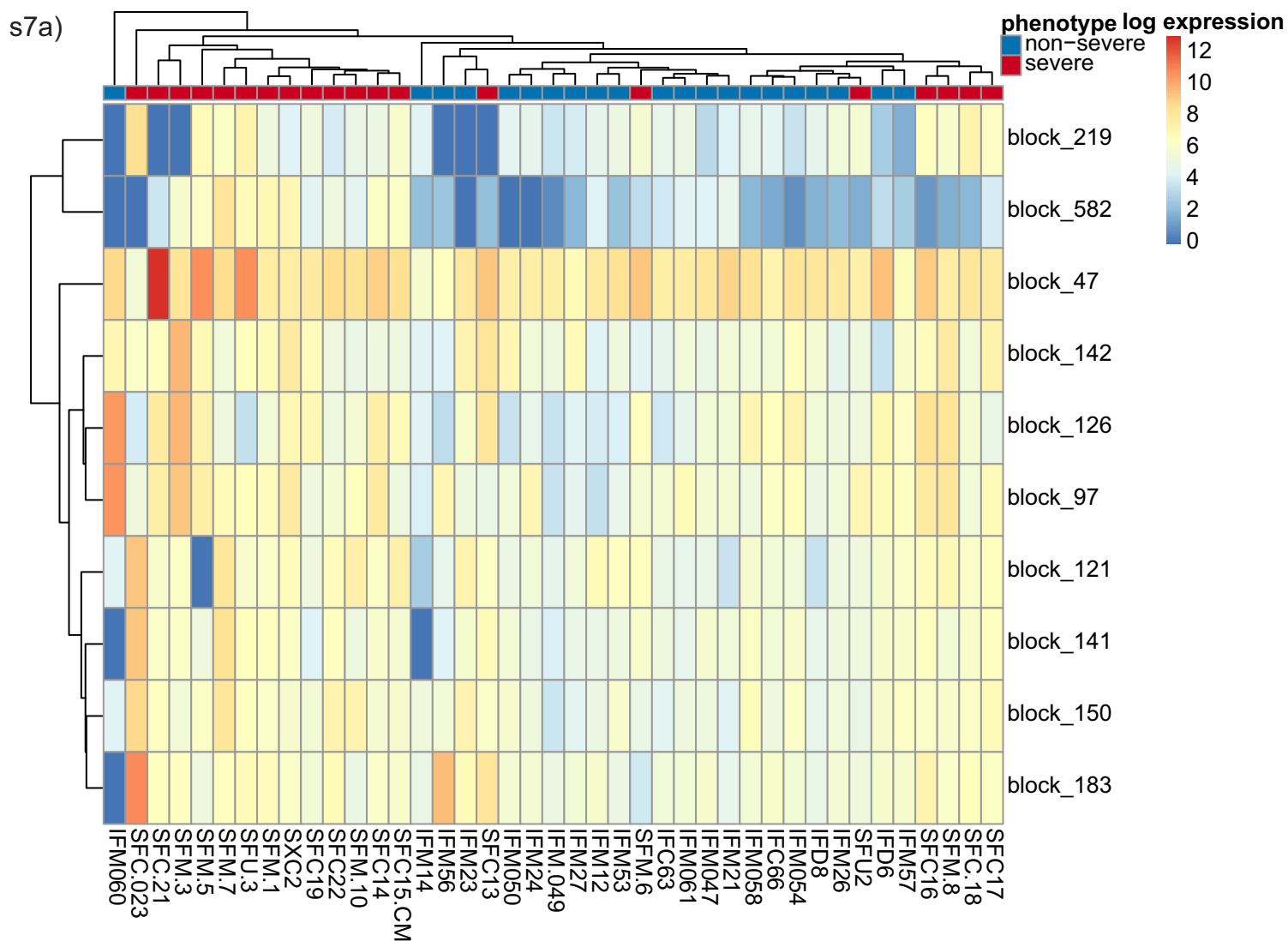

## VAR PCA – Rask Homology Blocks

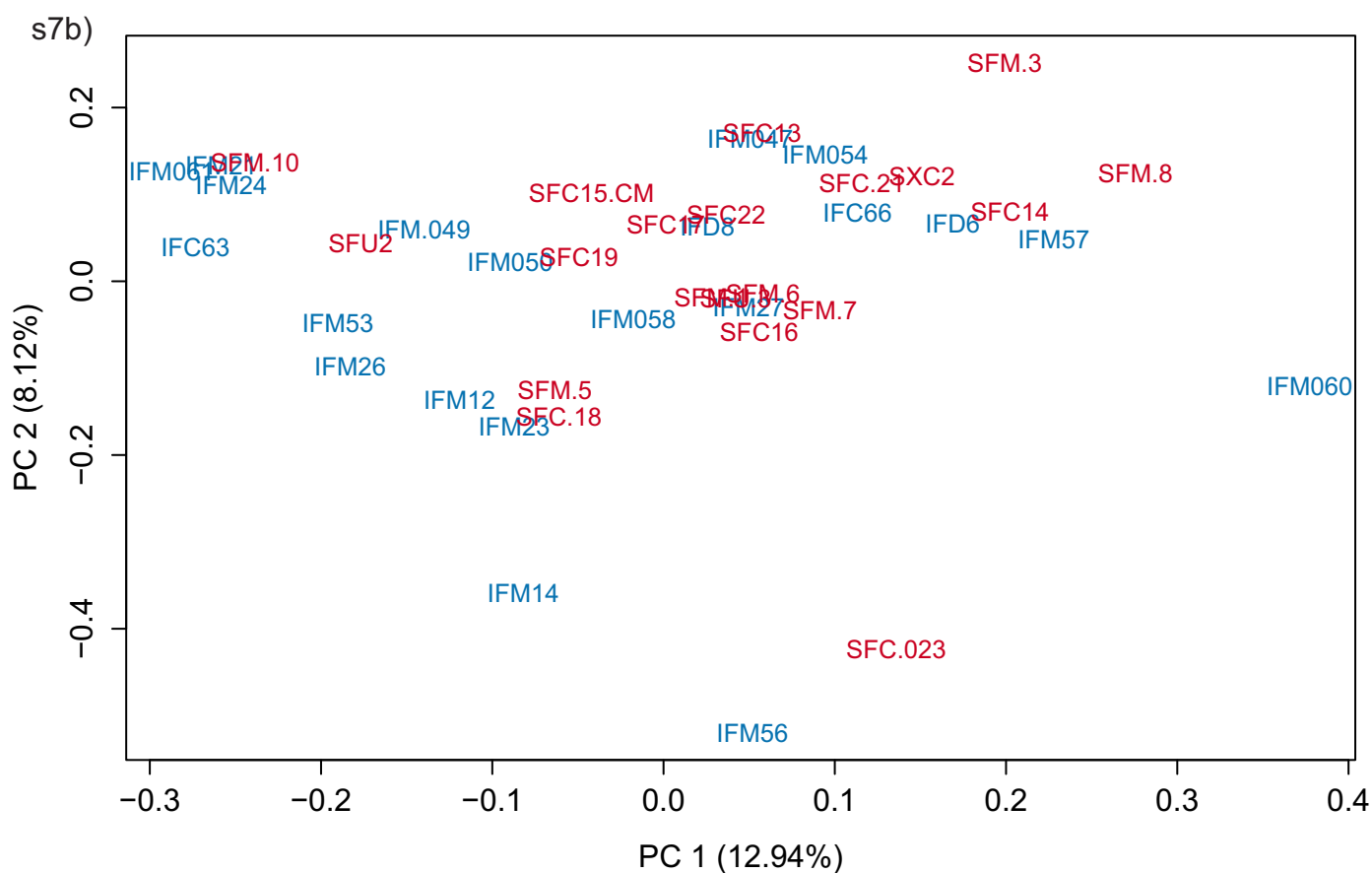

Supplement: S7 Fig — (A) Heatmap of expression level with patients’ samples and homology blocks grouped using complete linkage hierarchical clustering. (B) PCA plot of read counts annotated to homology blocks identified in transcripts from the separate sample assembly. This plot indicates that homology blocks do not separate severe disease as distinctly as the domain clusters. Raw read counts that were transformed for these figures are provided in S11 Data. (PDF) [file pbio.2004328.s007.pdf]

## DBL

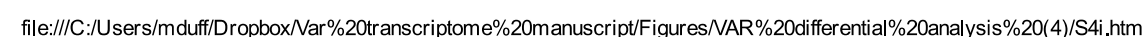

Supplement: S8 Fig — Logos of major domain classes produced using Skylign [101] on the output of Gismo [100]. Hierarchical clustering of segments was used for differential expression analysis. (PDF) [file pbio.2004328.s008.pdf]
