# Supplementary material for: The Plasmodium falciparum transcriptome in severe malaria reveals altered expression of genes involved in important processes including surface antigen–encoding var genes
Source: PLoS Biol. 2018 Mar 12;16(3):e2004328. doi: 10.1371/journal.pbio.2004328 (PMC5864071; doi:10.1371/journal.pbio.2004328)
Supplement: S6 Fig — Red dots are severe malaria samples SFC12, SFC14, SFC15, SFC17, SFC19, SFC22, SFM1, SFM3, SFU2, and SXC2; blue dots are uncomplicated malaria samples IFM049, IFM047, IFM050, IFM054, IFM12, IFM27, IFM53, and IFM56; black dots are samples that were excluded from the correlation analysis because they had high RPKM values, but the relevant transcripts lacked the Q-RT-PCR primer binding sites (670_X0.6—DBLε2, 348_X0.5—DBLγ3, 345_X0.5_DBLγ13.ns.1), or the PCR product had a different dissociation curve to all other products amplified with those primers (226_X0.6—DBLβ12). DBL, Duffy binding-like; Q-RT-PCR, quantitative reverse transcription PCR; RNAseq, RNA sequencing; RPKM, Reads Per Kilobase of transcript per Million mapped reads. (PDF) [file pbio.2004328.s006.pdf]

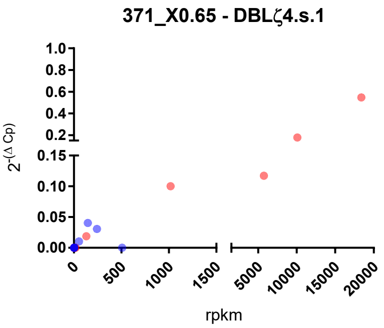

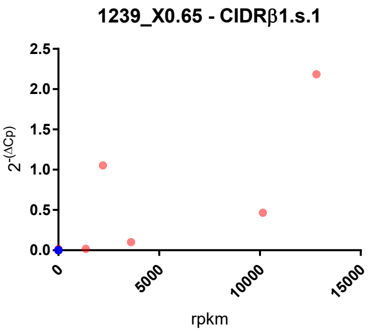

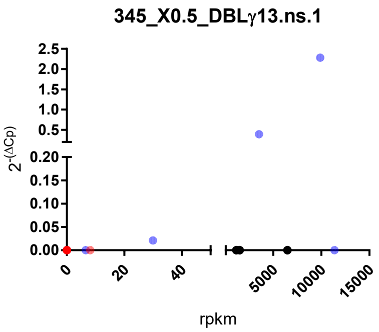

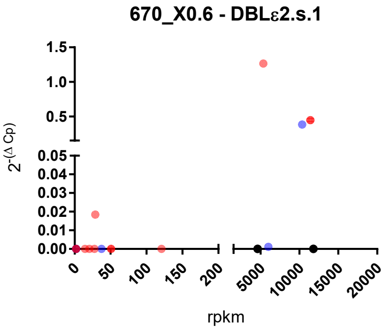

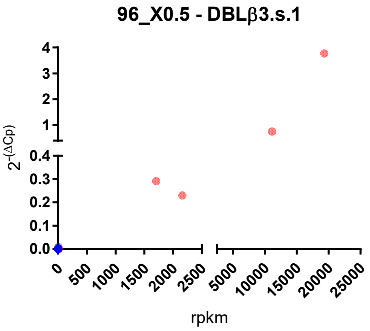

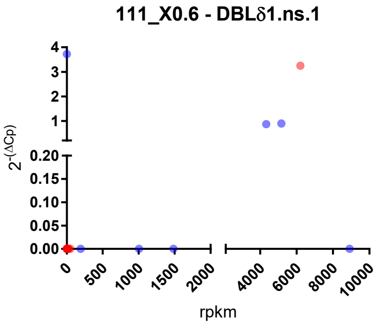

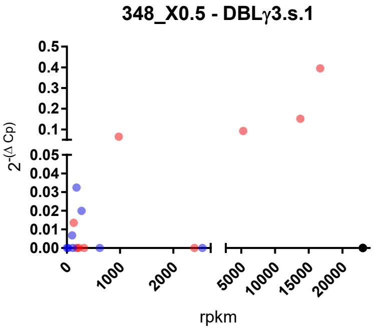

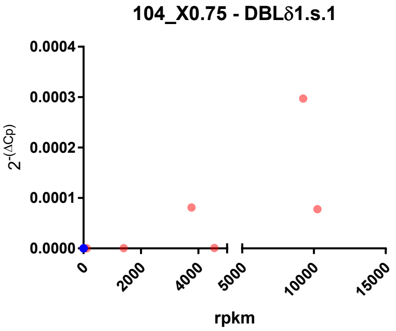

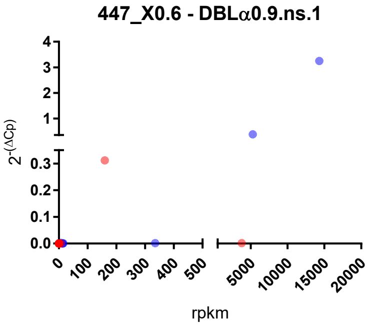

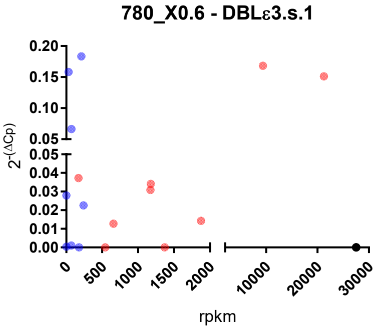

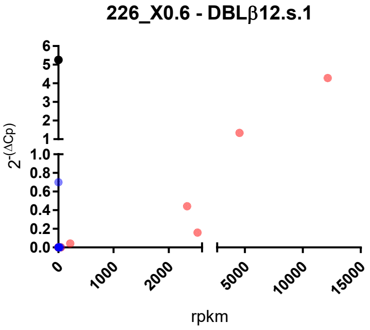

Correlations between quantitative RT-PCR and RNAseq

sequence	spearman R	p
371_X0.65 - DBLz4	0.8123	<0.0001
670_X0.6 - DBLe2	0.7007	0.0037
348_X0.5 - DBLg3	0.5318	0.0302
780_X0.6 - DBLe3	-0.04543	0.8579
1081_X0.97 - DBLe9	0.9951	<0.0001
1239_X0.65 - CIDRb1	0.8186	<0.0001
96_X0.5 - DBLb3	0.8514	<0.0001
104_X0.75 - DBLd1	0.8131	<0.0001
226_X0.6 - DBLb12	0.574	0.0181
Cluster-10.839_CIDRa2-6	0.5519	0.0176
345_X0.5_DBLg13.ns.1	0.5378	0.0255
111_X0.6 DBLd1.ns.1	0.6177	0.0063
447_X0.6_DBLa0.9.ns.1	0.831	<0.0001

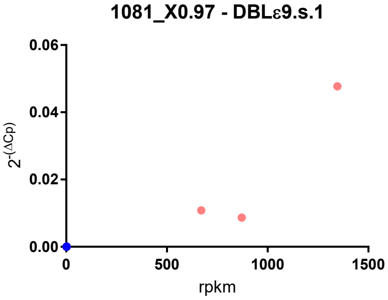

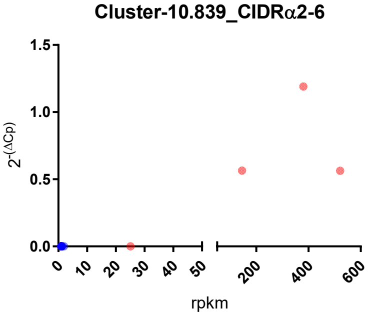

		IFM.049	IFM047	IFM050	IFM054	IFM12	IFM27	IFM53	IFM56	SFC13	SFC14	SFC15.CM	SFC17	SFC19	SFC22	SFM.1	SFM.3	SFU2	SXC2
371_x0.65-DBLz4.s.1	rpkm	147.1301	2	2.788144	2	53.9385	242.6204	508.1314	2	131.2794	10088.16	1017.993	2	2	9.156441	16.10279	5740.82	2	18378.94
371_x0.65-DBLz4.s.1	$2^{-(\Delta Cp)}$	0.040311	7.63E-06	0	0	0.010325	0.030784	0	0	0.01875	0.177736	0.100104	0	0	4.15E-06	0	0.11727	0	0.547911
670_x0.6-DBLe2.s.1	rpkm	50.72601	14.35406	5341.248	27.39356	2	20.51345	2	120.799	2	4603.054	11779.76	5999.575	2	37.23968	10331.92	2	28.64562	11397.99
670_x0.6-DBLe2.s.1	$2^{-(\Delta Cp)}$	2.74E-05	0	1.264312	0	0	0	0	0	0	0	0	0.001119	0	0	0.381716	1.67E-06	0.018385	0.446703
348_x0.5-DBLg3.s.1	rpkm	181.4941	24.75459	2546.778	2	100.1392	274.7951	616.7955	111.4062	127.6281	13758.63	975.5852	183.3647	324.0645	2395.444	23009.53	5267.604	222.8501	16694.3
348_x0.5-DBLg3.s.1	$2^{-(\Delta Cp)}$	0.032511	0	0	0	0.006783	0.019946	0	0	0.013527	0.152097	0.065293	0	0	2.43E-07	0	0.092638	0	0.39492
780_x0.6-DBLe3.s.1	rpkm	239.1202	71.36898	69.55505	30.51735	177.5214	209.9088	2	2	1176.491	27536.02	1874.794	1369.179	656.5576	170.1924	1171.814	21248.54	540.6216	9344.062
780_x0.6-DBLe3.s.1	$2^{-(\Delta Cp)}$	0.022546	0.066243	0.001024	0.158357	3.12E-05	0.18338	0.027843	0.0004	0.0341	2.75E-05	0.014241	0.000083	0.012715	0.037294	0.03086	0.151252	1.9E-06	0.168127
1081_x0.97-DBLe9.s.1	rpkm	2	2	2	2	2	2	2	2	1346.218	2	2	2	670.3314	2	2	870.8607	2	2
1081_x0.97-DBLe9.s.1	$2^{-(\Delta Cp)}$	0	0	0	0	0	0	0	0	0.047736	0	0	0	0.010835	0	0	0.00866	0	0
1239_x0.65-CIDRb1.s.1	rpkm	2	2	2	2	2	2	2	2	2213.323	1364.285	2	2	2	2	12807.19	3601.059	2	10143.18
1239_x0.65-CIDRb1.s.1	$2^{-(\Delta Cp)}$	0	7.32E-06	0	9.59E-06	0	0	0	0	1.052413	0.018323	1.23E-06	0.017127	0	0	2.183644	0.100739	6.49E-05	0.466629
96_x0.5-DBLb3.s.1	rpkm	2	2	4.240707	2	2	2	2	2	19324.29	2	2	2	1707.019	8.781961	2	11155.58	2	2162.645
96_x0.5-DBLb3.s.1	$2^{-(\Delta Cp)}$	0	0	0.007238	0	0	0	0	0	3.766605	0	0	0	0.290855	0	3.77E-06	0.752204	0	0.229844
104_x0.75-DBLd1.s.1	rpkm	2	2	2.674075	2	2	2	2	2	3761.333	1403.238	12.34461	14.43823	2	14.24136	9253.302	4556.673	113.072	10245.21
104_x0.75-DBLd1.s.1	$2^{-(\Delta Cp)}$	0	0	0	0	0	0	0	0	8.12E-05	4.1E-07	0	0	0	0	0.000297	7.57E-07	0	7.77E-05
226_x0.6-DBLb12.s.1	rpkm	13.77979	10.95999	4.111056	2	2	2	34.27811	2	12154.67	47.71223	2	2	4539.273	2	2331.429	2520.497	214.5771	2
226_x0.6-DBLb12.s.1	$2^{-(\Delta Cp)}$	3.28E-06	4.68E-06	3.59E-06	1.75E-05	5.260346	4.24E-06	0.000348	0.699982	4.279898	4.87E-07	7.38E-07	8.17E-06	1.33919	4.84E-07	0.442685	0.159079	0.041612	1.5E-06
cluster-10.839_CIDRa2.6	rpkm	1	1	1	1.962038	1.286983	1	1	1	380.1801	1	1	1	145.4145	25.09586	1	521.0357	1	1
cluster-10.839_CIDRa2.6	$2^{-(\Delta Cp)}$	1.59E-05	7.08E-06	1.17E-05	7.24E-05	1.34E-05	5.7E-06	0.000531	0.000159	1.19121	1.41E-06	8.94E-06	8.66E-06	0.564879	3.28E-06	1.05E-05	0.563605	5.02E-07	2.38E-07
345_x0.5-DBLg13.ns.1	rpkm	29.97967	9885.215	6480.32	3515.378	6.556605	1161.859	11377.51	0	0	0	0	1503.59	0	8.130724	0	0	0	0
345_x0.5-DBLg13.ns.1	$2^{-(\Delta Cp)}$	0.021052	2.281477	0	0.391522	0	0	0	0	0	0	0	0	0	0	0	6.23E-06	0	0
111_x0.6-DBLd1.ns.1	rpkm	191.8298	1484.853	5161.392	1005.602	0	4334.558	0	8913.825	47.38656	0	6206.763	0	0	12.24136	24.12334	0	18.512	0
111_x0.6-DBLd1.ns.1	$2^{-(\Delta Cp)}$	6.49E-06	5.54E-06	0.893314	3.69E-05	0.000001	0.865682	3.724809	1.21E-05	1.2E-06	2.46E-07	3.248032	4.86E-06	2.33E-07	1.89E-07	3.91E-06	1.11E-06	2.91E-07	2.78E-07
447_x0.6-DBLa09.ns.1	rpkm	13.58941	14315.96	6.494289	5269.098	11.88811	15.48989	335.1303	0	0	0	0	3774.787	0	7.371112	159.7841	0	0	0
447_x0.6-DBLa09.ns.1	$2^{-(\Delta Cp)}$	7.87E-05	3.247326	0.002816	0.382413	0.00023	0.00123	0.001319	9.48E-05	1.89E-06	0	0	0.001642	0	0	0.311959	0	3.62E-07	0
