# Supplementary material for: The Plasmodium falciparum transcriptome in severe malaria reveals altered expression of genes involved in important processes including surface antigen–encoding var genes
Source: PLoS Biol. 2018 Mar 12;16(3):e2004328. doi: 10.1371/journal.pbio.2004328 (PMC5864071; doi:10.1371/journal.pbio.2004328)
Supplement: S1 Text — (DOCX) [file pbio.2004328.s009.docx]

Supplementary methods

RNA extraction and RNAseq

CF11 cellulose was packed dry into 10 ml columns and then pre-wetted with 10 ml PBS. Blood was applied to the column and the column then washed with PBS until the erythrocytes had visibly eluted. The erythrocytes were pelleted by centrifugation at 500 g for 5 min at RT and then the erythrocyte pellet was resuspended in an equal volume of PBS and passed over another CF11 cellulose column as above. The resulting erythrocyte pellet was dissolved in 10 pellet volumes of TRIzol (Invitrogen Thermo Fisher Scientific MA USA) prewarmed to 37°C and then incubated at 37°C for 5 min prior to storage at -80°C.

Erythrocytes in TRIzol were thawed at 37°C, chloroform (1/5th of the TRIzol volume) was added and vortexed 15 sec, the solution was then subjected to centrifugation at 12,000 x g for 30 minutes at 4°C and the aqueous supernatant was aspirated and mixed with an equal volume of 70% ethanol in RNase free water. This solution was then directly applied to RNeasy mini columns (QIAGEN Germany), and RNA purification was performed with on-column DNasing as per the manufacturer’s instructions. DNasing was confirmed by quantitative PCR and if necessary was repeated by in solution DNasing followed by RNeasy mini cleanup as per manufacturer’s instructions.

One to three μg purified RNA was depleted of hemoglobin mRNA using the Globinclear human hemoglobin RNA depletion kit (Ambion, Thermo Fisher Scientific) and a modified protocol. Briefly the manufacturer’s protocol was followed until removal of globin-depleted RNA from the pelleted streptavidin magnetic beads with captured globin mRNA. The 60 μl aspirated globin-depleted RNA was then precipitated at -80°C overnight with 180 μl ethanol, 6 μl 3M sodium acetate pH 5.5 and 1.8 μl 5 mg/ml linear acylamide. The RNA was pelleted by centrifugation at 20,000 g, 4°C for 60 minutes and the pellet then washed in ice cold 70% ethanol with a 20 min centrifugation as above. The RNA pellet was air dried briefly then dissolved in 51 μl nuclease free water on ice. RNA integrity was assessed using Experion High sensitivity RNA chips (BioRad USA).

Sequencing

The NEBnext ultra directional RNA library prep kit for Illumina (New England Biolabs, MA USA) was used as per the manufacturer’s instructions with a few modifications (Supplementary methods). mRNA Fragmentation was empirically optimised as 94°C for 10 minutes, to generate 200 bp fragments. Adapter ligated DNA was size selected using 0.5 times sample volume AMPure XP beads in buffer to bind and remove large fragments and then 0.8 times sample volume beads buffer (i.e. 0.3 times sample volume fresh beads added) to bind and retain intermediate DNA fragments. This generated libraries of approximately 330 bp that contained approximately 200 bp inserts. Ligated RNA was amplified in a 50 μl reaction containing NEB User enzyme 3 μl (for excision of uracil in loop adapters), the NEBNext Universal PCR primer for Illumina and NEBNext Indexed primers for Illumina both at 0.5 μM, 0.3 mM each dNTP, 1 x Kapa HiFi buffer (containing TMAC) (Kapa Biosystems, Roche USA) and 1 unit of Kapa HiFi. The PCR reaction was incubated at 37°C for 15 min, 1 min at 98°C and then 15 cycles of 98°C for 10s and 65°C for 1 min followed by a 5 min extension at 65°C. The amplified library was then purified by binding to 0.8 times volume of AMPure XP beads and buffer. Libraries were quantitated by SYBR green quantitative PCR using P1 and P2 Illumina PCR primers and by qubit (invitrogen). Library quality was assessed using a Bioanalyser high sensitivity DNA chip (Agilent CA USA).

**Untargeted LC-MS profiling**

Samples were vortexed and centrifuged at 14,000 x g for 5 minutes at 4 °C. The supernatant was collected and transferred to MS vials for LC-MS . Data was converted to mzXML and analysed using the MAVEN software package (PMID: 22389014). M/z features were extracted with a 10 parts per million (ppm) mass window and a pairwise comparison between severe and uncomplicated samples was performed using a Benjamini-corrected t test. Statistical significance was determined using p < 0.01 and significant features were searched in the METLIN database for putative identification. Putative identifications were then curated for possible adducts and isotopologues and finally identification was confirmed using pure standards. In addition, known metabolites of interest were extracted using a reference library of approximately 150 standards and plotted as average and 95% confidence intervals.

***De novo assembly of var genes***

**Assembly**

Figure S2c indicates the pipeline used to assemble the VAR gene transcripts. Initially Trim Galore v0.3.7 [[1]](https://paperpile.com/c/0OP2FX/hNK3Y) (--phred33 --paired) was used to perform adapter and quality trimming. The reads were then aligned to the *H. sapiens*, *P. vivax* and *P. falciparum* reference genomes using the Subread aligner v1.4.6 [[2]](https://paperpile.com/c/0OP2FX/kyXEk) with default parameters. Any reads that aligned to the *var* gene regions of the *P.falciparum* genome were then extracted using bedtools v2.20.1 [[3]](https://paperpile.com/c/0OP2FX/SOqf7). These were grouped with reads that did not align to the reference genomes. As the majority of paired reads overlapped, Pear v0.9.0 [[4]](https://paperpile.com/c/0OP2FX/tR1Vm) was used to merge reads. Digital normalisation [[5]](https://paperpile.com/c/0OP2FX/nahhB) was then performed to improve the time performance of the assembly. Finally SoapDeNovo-Trans v1.03 [[6]](https://paperpile.com/c/0OP2FX/sOXVR) was run with Kmer values of 21, 31, 41, 51 and 61 (-K Kmer -L 50). The resulting contig files were then merged with cap3 version date 21/12/07 [[7]](https://paperpile.com/c/0OP2FX/6fmZ2) ( -p 99 -o 200) as described in the pipeline of [[8]](https://paperpile.com/c/0OP2FX/gVisn). The pipeline was implemented in python. The code used as well as the config file parameters used in the SoapDeNovo run and the *var* gene gff annotation files are available at <https://github.com/PapenfussLab/assemble_var>.

**Transcript translation and filtering**

After assembly, transcripts were kept if they were greater than 500nt in length and did not align to the *H. sapiens* or non-*var* *P.vivax* or *P.falciparum* genomes by more than 30% using BLAST [[9]](https://paperpile.com/c/0OP2FX/EjLZ1). Any remaining contigs were searched against NCBI’s nt database [[10]](https://paperpile.com/c/0OP2FX/NTGwp) and manually inspected to remove remaining contaminants. Transcripts were then translated into all six reading frames and the correct frame was identified by requiring that at least two distinct homology blocks of [[11]](https://paperpile.com/c/0OP2FX/ljhQm) aligned correctly using HMMER.

***All gene expression analysis***

Reads were first aligned to the *H. sapiens* and *P. falciparum* reference genomes using Subread-align v1.4.6 [[12]](https://paperpile.com/c/0OP2FX/8JlF7) with parameters -u -H.

**Read annotation**

Alignment to the *H. sapiens* and *P. falciparum* reference genomes was performed using Subread-align v1.4.6 [[12]](https://paperpile.com/c/0OP2FX/8JlF7) with parameters -u -H ensuring only uniquely mapped reads were aligned. Gene level counts were obtained using the R version of subreads featureCounts v1.20.2 [[13]](https://paperpile.com/c/0OP2FX/R7dcC) in paired-end mode summarising counts at the gene level. Otherwise default parameters were used.

**Estimating life cycle stage and other unwanted sources of variation**

To account for parasite lifecycle, each sample is estimated as a mixture of six parasite lifecycle stages; ring, early trophozoite, late trophozoite, schizont, gametocyte I and gametocyte II. These corresponded to six of the seven lifecycle stages sequenced in [[14]](https://paperpile.com/c/0OP2FX/zKA2B). As the samples were taken from human host it is unlikely that there are any ookinetes present, consequently this stage was left out of the mixture model.

Log2 transforms of the RPKM values for both our samples and the reference profiles from the different stages of [[14]](https://paperpile.com/c/0OP2FX/zKA2B) were taken. We then fit a mixture model, where we wish to choose proportions **π** of the stage profiles **S** which will explain the profile in one of our samples. Here $g_{i,s}$ represents the expression of the ith gene in sample s.

That is we wish to choose **π** for each sample to minimise

$$\sum_{i=1}^{N} (g_{i, sample}-\sum_{s\in S} \pi_{s}g_{i, s})^{2}$$

subject to the constraints

$$\sum_{s\in S} \pi_{s}=1$$

and

$$\pi_{s}\geq0$$

The function solve.QP from R’s quadprog package was used to fit this model.

Three factors of unwanted variation were estimated using the RUV4 function from the R package ruv v0.9.6 [[15]](https://paperpile.com/c/0OP2FX/F5LJr). Both the disease severity and the proportion of ring stage estimated by the mixture model were passed to RUV4 as known variables. The 1009 genes with the lowest p-values from [[16]](https://paperpile.com/c/0OP2FX/eiexG) which are also present in our dataset were used as controls. The choice of control genes was compared to using the least differentially expressed genes of [[17]](https://paperpile.com/c/0OP2FX/km9l5) which was found to give similar results.

**Voom/Limma analysis**

The gene counts along with the estimated ring stage factor and three factors of unwanted variation estimated by RUV4 were fed into the Limma/Voom [[18,19]](https://paperpile.com/c/0OP2FX/bMHIb+5I593) differential analysis pipeline. Default parameters were used except for the eBayes function where the robust option was implemented to account for dispersion outliers. The Benjamini-Hochberg [[20]](https://paperpile.com/c/0OP2FX/ijkVU) method was used to control for multiple testing. For a detailed outline of the specific commands run in the all gene analysis see Supplementary Text S1 in the github repository.

***Transcript level***

**Separate assembly - Corset**

First the reads were trimmed and quality filtered as described in the assembly pipeline. Bowtie v0.12.9 [[21]](https://paperpile.com/c/0OP2FX/ykmGV) was then used to align the reads to the assembled transcripts of each sample with option --all specified to allow for multiple alignments of the same read. The resulting sam files were converted to bam files with samtools v1.2 [[22]](https://paperpile.com/c/0OP2FX/YazoD). Corset v1.03 [[23]](https://paperpile.com/c/0OP2FX/V629W) was run on the bam files with default options and the classification of each sample into severe and non-severe disease. The resulting counts for each cluster were then filtered for low abundance clusters and differential expression estimates were made using the default DESeq2 pipeline [[24]](https://paperpile.com/c/0OP2FX/bKrti). A more detailed description can be found in the Supplementary Text S3 available in the github repository.

**Combined assembly - Subreads**

A combined assembly was produced using the assembly pipeline on the pooled reads of all samples. Subreads v1.4.6 [[12]](https://paperpile.com/c/0OP2FX/8JlF7) was used to align the reads for each sample to the combined assembly transcripts. Subreads was run with parameters -u -H to ensure only uniquely mapped reads were counted. A count matrix was produced using the R version of featureCounts v1.20.2 [[13]](https://paperpile.com/c/0OP2FX/R7dcC) in paired end mode and a SAF file to describe each transcript. The count matrix was then filtered for transcripts with low expression and DESeq2 with default parameters was used to estimate differential expression significance. The R markdown code for both these analyses is available in Supplementary Text S2.

***Type/Domain level***

**Domain annotation**

HMMER3’s hmmsearch v3.1b1 [[25]](https://paperpile.com/c/0OP2FX/OY6dd) was used to search the NTS, DBL and CIDR domain models of [[11]](https://paperpile.com/c/0OP2FX/ljhQm) against the assembled transcripts from each sample. The most significant domain was annotated first and then successively less significant domains with the requirement that two domains do not overlap. An E-value threshold of 1e-8 was chosen to minimise spurious annotations.

**Read Annotation**

Reads were annotated to the separate assembly transcripts using Subreads v1.4.6 taking the best match for each read. As we later cluster the domains based on domain type and sequence identity, taking the best match for each read is appropriate and has been shown to produce similar results to other approaches such as RSEM [[23]](https://paperpile.com/c/0OP2FX/V629W). FeatureCounts was used to allocate reads to domains, using a SAF file built from the HMMER3 annotations.

**Previous domain/block classification**

The read counts were aggregated based on the NTS, DBL and CIDR domain types of [[11]](https://paperpile.com/c/0OP2FX/ljhQm). Differential expression testing was conducted using the default DESeq2 [[24]](https://paperpile.com/c/0OP2FX/bKrti) pipeline. This was repeated using the homology blocks defined in [[11]](https://paperpile.com/c/0OP2FX/ljhQm).

**Novel domain classification**

**Hierarchical clustering of domains using Usearch**

Hierarchical clustering with USEARCH is achieved by first clustering the domains, sorted by length, by successively lower identity thresholds. The exemplars from the previous round of clustering are fed to the next round. For lower identity thresholds this does not guarantee that every sequence is within the threshold of its exemplar. However, it sensibly handles fragments of domains whilst also being computationally efficient. The read counts for each domain are then aggregated up this hierarchical tree. See code in the github repository for a more detailed explanation. The github repository also contains images of the hierarchical trees which make up each of these newly identified domain clusters and which were created using the ete2 python package [[26]](https://paperpile.com/c/0OP2FX/qVPtx) together with their respective multiple sequence alignments which were performed using muscle v3.8.31 [[27]](https://paperpile.com/c/0OP2FX/JX3vv).

**Differential expression**

The default DESeq2 pipeline was run with the counts for each cluster of the hierarchical tree.

The Benjamini-Yekutieli [[28]](https://paperpile.com/c/0OP2FX/hUWoM) method for multiple testing was used. This correction allows for the dependence between clusters in the hierarchy. After multiple testing correction, we iteratively reject the null hypothesis (p<0.05) of the most significant node before removing its ancestor and children nodes from those available. This ensures we select the most significant grouping of domains from which to form clusters. The Rmarkdown code for the domain analysis along with a more thorough description of the methods is available in Supplementary Text S4 in the github repository.

***Segment level***

**Homology block analysis**

HMMER3 [[29]](https://paperpile.com/c/0OP2FX/pOHyz) profile hidden Markov models of 613 of the possible 628 homology blocks of [[11]](https://paperpile.com/c/0OP2FX/ljhQm) were aligned to the separate assembly transcripts using a bit score cutoff of 9.97 as is described in [[11]](https://paperpile.com/c/0OP2FX/ljhQm). A SAF file was created from these alignments and featureCounts was used to summarise the read count for each homology block in each sample. Here we made use of the same read alignments as the domain level analysis. The resulting counts were then analysed for differential expression using the default DESeq2 pipeline [[30]](https://paperpile.com/c/0OP2FX/Xewve).

**Identification of novel conserved segments**

Initially VAR domains were identified as in the domain section. Major domain classes were aligned using gismo v2.0 [[31]](https://paperpile.com/c/0OP2FX/Mxv62). The resulting alignments were then segmented into regions of high and low occupancy. If seven or more consecutive columns within an alignment had an occupancy greater than 95% these columns were considered a conserved region. Terminal gaps were not counted in the occupancy calculations. The columns in between these conserved regions were considered as variable regions. Each domain sequence was then split into segments based on the regions and the segments were clustered hierarchically within each region.

**Hierarchical clustering**

Each region was clustered separately using CD-HIT v4.6 [[32]](https://paperpile.com/c/0OP2FX/XocI7) at various identity thresholds to produce a hierarchical clustering. Sequences were first ordered in descending order by the number of times they occurred in the region. This helps to prevent outlier sequences from forming the representative sequence of a cluster. Clustering was initially performed at 97% pairwise sequence identity with the representatives of each cluster being used in the subsequent clustering steps at 95, 90, 85, 80, 75, 70, 65, 60 and 50%. A python script was written to sequentially run cd-hit and combine the results and is available from the github repository.

**Differential expression**

Differential expression was performed in the same manner as in the hierarchical domain analysis. A more thorough description of the method for identifying the segments is given in Supplementary Text S6 available in the github repository.

1. [Krueger F. Trim Galore [Internet]. Available:](http://paperpile.com/b/0OP2FX/hNK3Y) <http://www.bioinformatics.babraham.ac.uk/projects/trim_galore/>

2. [Liao Y, Smyth GK, Shi W. The Subread aligner: fast, accurate and scalable read mapping by seed-and-vote. Nucleic Acids Res. 2013;41: e108.](http://paperpile.com/b/0OP2FX/kyXEk)

3. [Quinlan AR, Hall IM. BEDTools: a flexible suite of utilities for comparing genomic features. Bioinformatics. 2010;26: 841–842.](http://paperpile.com/b/0OP2FX/SOqf7)

4. [Zhang J, Kobert K, Flouri T, Stamatakis A. PEAR: a fast and accurate Illumina Paired-End reAd mergeR. Bioinformatics. 2014;30: 614–620.](http://paperpile.com/b/0OP2FX/tR1Vm)

5. [Brown CT, Howe A, Zhang Q, Pyrkosz AB, Brom TH. A Reference-Free Algorithm for Computational Normalization of Shotgun Sequencing Data. arXiv:12034802 [q-bio]. 2012; Available:](http://paperpile.com/b/0OP2FX/nahhB) <http://arxiv.org/abs/1203.4802>

6. [Xie Y, Wu G, Tang J, Luo R, Patterson J, Liu S, et al. SOAPdenovo-Trans: de novo transcriptome assembly with short RNA-Seq reads. Bioinformatics. 2014;30: 1660–1666.](http://paperpile.com/b/0OP2FX/sOXVR)

7. [Huang X, Madan A. CAP3: A DNA Sequence Assembly Program. Genome Res. 1999;9: 868–877.](http://paperpile.com/b/0OP2FX/6fmZ2)

8. [Yang Y, Ya Y, Smith SA. Optimizing de novo assembly of short-read RNA-seq data for phylogenomics. BMC Genomics. 2013;14: 328.](http://paperpile.com/b/0OP2FX/gVisn)

9. [Altschul SF, Gish W, Miller W, Myers EW, Lipman DJ. Basic local alignment search tool. J Mol Biol. 1990;215: 403–410.](http://paperpile.com/b/0OP2FX/EjLZ1)

10. [Camacho C, Coulouris G, Avagyan V, Ma N, Papadopoulos J, Bealer K, et al. BLAST+: architecture and applications. BMC Bioinformatics. 2009;10: 421.](http://paperpile.com/b/0OP2FX/NTGwp)

11. [Rask TS, Hansen DA, Theander TG, Gorm Pedersen A, Lavstsen T. Plasmodium falciparum Erythrocyte Membrane Protein 1 Diversity in Seven Genomes – Divide and Conquer. PLoS Comput Biol. 2010;6: e1000933.](http://paperpile.com/b/0OP2FX/ljhQm)

12. [Liao Y, Smyth GK, Shi W. The Subread aligner: fast, accurate and scalable read mapping by seed-and-vote. Nucleic Acids Res. 2013;41: e108.](http://paperpile.com/b/0OP2FX/8JlF7)

13. [Liao Y, Smyth GK, Shi W. featureCounts: an efficient general purpose program for assigning sequence reads to genomic features. Bioinformatics. 2014;30: 923–930.](http://paperpile.com/b/0OP2FX/R7dcC)

14. [López-Barragán MJ, Lemieux J, Quiñones M, Williamson KC, Molina-Cruz A, Cui K, et al. Directional gene expression and antisense transcripts in sexual and asexual stages of Plasmodium falciparum. BMC Genomics. 2011;12: 587.](http://paperpile.com/b/0OP2FX/zKA2B)

15. [Gagnon-Bartsch JA, Speed TP. Using control genes to correct for unwanted variation in microarray data. Biostatistics. 2012;13: 539–552.](http://paperpile.com/b/0OP2FX/F5LJr)

16. [Vignali M, Armour CD, Chen J, Morrison R, Castle JC, Biery MC, et al. NSR-seq transcriptional profiling enables identification of a gene signature of Plasmodium falciparum parasites infecting children. J Clin Invest. 2011;121: 1119–1129.](http://paperpile.com/b/0OP2FX/eiexG)

17. [Yamagishi J, Natori A, Tolba MEM, Mongan AE, Sugimoto C, Katayama T, et al. Interactive transcriptome analysis of malaria patients and infecting Plasmodium falciparum. Genome Res. 2014;24: 1433–1444.](http://paperpile.com/b/0OP2FX/km9l5)

18. [Law CW, Chen Y, Shi W, Smyth GK. voom: Precision weights unlock linear model analysis tools for RNA-seq read counts. Genome Biol. 2014;15: R29.](http://paperpile.com/b/0OP2FX/bMHIb)

19. [Smyth GK. limma: Linear Models for Microarray Data. Bioinformatics and Computational Biology Solutions Using R and Bioconductor. Springer New York; 2005. pp. 397–420.](http://paperpile.com/b/0OP2FX/5I593)

20. [Benjamini Y, Hochberg Y. Controlling the False Discovery Rate: A Practical and Powerful Approach to Multiple Testing. J R Stat Soc Series B Stat Methodol. 1995;57: 289–300.](http://paperpile.com/b/0OP2FX/ijkVU)

21. [Langmead B, Trapnell C, Pop M, Salzberg SL. Ultrafast and memory-efficient alignment of short DNA sequences to the human genome. Genome Biol. 2009;10: R25.](http://paperpile.com/b/0OP2FX/ykmGV)

22. [Li H, Handsaker B, Wysoker A, Fennell T, Ruan J, Homer N, et al. The Sequence Alignment/Map format and SAMtools. Bioinformatics. 2009;25: 2078–2079.](http://paperpile.com/b/0OP2FX/YazoD)

23. [Davidson NM, Oshlack A. Corset: enabling differential gene expression analysis for de novo assembled transcriptomes. Genome Biol. 2014;15: 410.](http://paperpile.com/b/0OP2FX/V629W)

24. [Love MI, Huber W, Anders S. Moderated estimation of fold change and dispersion for RNA-seq data with DESeq2. Genome Biol. 2014;15: 550.](http://paperpile.com/b/0OP2FX/bKrti)

25. [Eddy SR. A new generation of homology search tools based on probabilistic inference. Genome Inform. 2009;23: 205–211.](http://paperpile.com/b/0OP2FX/OY6dd)

26. [Huerta-Cepas J, Dopazo J, Gabaldón T. ETE: a python Environment for Tree Exploration. BMC Bioinformatics. 2010;11: 24.](http://paperpile.com/b/0OP2FX/qVPtx)

27. [Edgar RC. MUSCLE: multiple sequence alignment with high accuracy and high throughput. Nucleic Acids Res. 2004;32: 1792–1797.](http://paperpile.com/b/0OP2FX/JX3vv)

28. [Benjamini Y, Yekutieli D. The Control of the False Discovery Rate in Multiple Testing under Dependency. Ann Stat. 2001;29: 1165–1188.](http://paperpile.com/b/0OP2FX/hUWoM)

29. [Eddy SR. A new generation of homology search tools based on probabilistic inference. Genome Inform. 2009;23: 205–211.](http://paperpile.com/b/0OP2FX/pOHyz)

30. [Love MI, Huber W, Anders S. Moderated estimation of fold change and dispersion for RNA-seq data with DESeq2. Genome Biol. 2014;15: 550.](http://paperpile.com/b/0OP2FX/Xewve)

31. [Neuwald AF, Altschul SF. Bayesian Top-Down Protein Sequence Alignment with Inferred Position-Specific Gap Penalties. PLoS Comput Biol. 2016;12: e1004936.](http://paperpile.com/b/0OP2FX/Mxv62)

32. [Li W, Godzik A. Cd-hit: a fast program for clustering and comparing large sets of protein or nucleotide sequences. Bioinformatics. 2006;22: 1658–1659.](http://paperpile.com/b/0OP2FX/XocI7)
