# Supplementary material for: The Plasmodium falciparum transcriptome in severe malaria reveals altered expression of genes involved in important processes including surface antigen–encoding var genes
Source: PLoS Biol. 2018 Mar 12;16(3):e2004328. doi: 10.1371/journal.pbio.2004328 (PMC5864071; doi:10.1371/journal.pbio.2004328)
Supplement: S2 Text — CIDR, cysteine-rich interdomain region; DBL, Duffy binding-like; DC, domain cassette. (PDF) [file pbio.2004328.s010.pdf]

Supplementary domain trees

Gerry Tonkin-Hill

2017-02-17

Load libraries

```
library(ggplot2)
library(ape)
```

Phylogentic method

Protein domains were aligned using MUSCLE v3.8.31 and phylogentic trees were constructed using FastTree version 2.1.7 SSE3

```
muscle -in domains.fasta -out MA_domains.fasta
FastTree < MA_domains.fasta > MA_domains.tree
```

DC5 Comparison

Bengtsson et al. found that DC4 may be related to PfEMP1 proteins binding to ICAM-1. In particular they identified a ditinct clade of DBLb3 domains that bound to ICAM-1. The tree drawn below indicates that the first DBLb3 domain of PFD125w (one of those found in the clade) clusters closest to the severe cluster DBLb.s.2

Lavsten et al 2012 also found DBLb3 to be more expressed in children with severe malaria.

```
dbl_b3 <- read.tree("./data/compare_DBLb3/combined_vardom_severe_DBLb3.phylip")

labels <- "black"
labels[grepl("\\.s\\.1", dbl_b3$tip.label)] <- "#4daf4a"
labels[grepl("\\.s\\.2", dbl_b3$tip.label)] <- "#f781bf"
labels[grepl("PFD1235w", dbl_b3$tip.label)] <- "#e41a1c"

plot.phylo(dbl_b3, type = "phylogram", use.edge.length = FALSE, show.node.label = TRUE,
    cex = 0.5, tip.color = labels, align.tip.label = TRUE)
legend(1, -1, legend = c("DBLb3", "DBLb3.s.1", "DBLb3.s.2", "PFD1235w - Bengsston"),
    fill = c("black", "#4daf4a", "#f781bf", "#e41a1c"), xpd = NA, cex = 0.5)
```

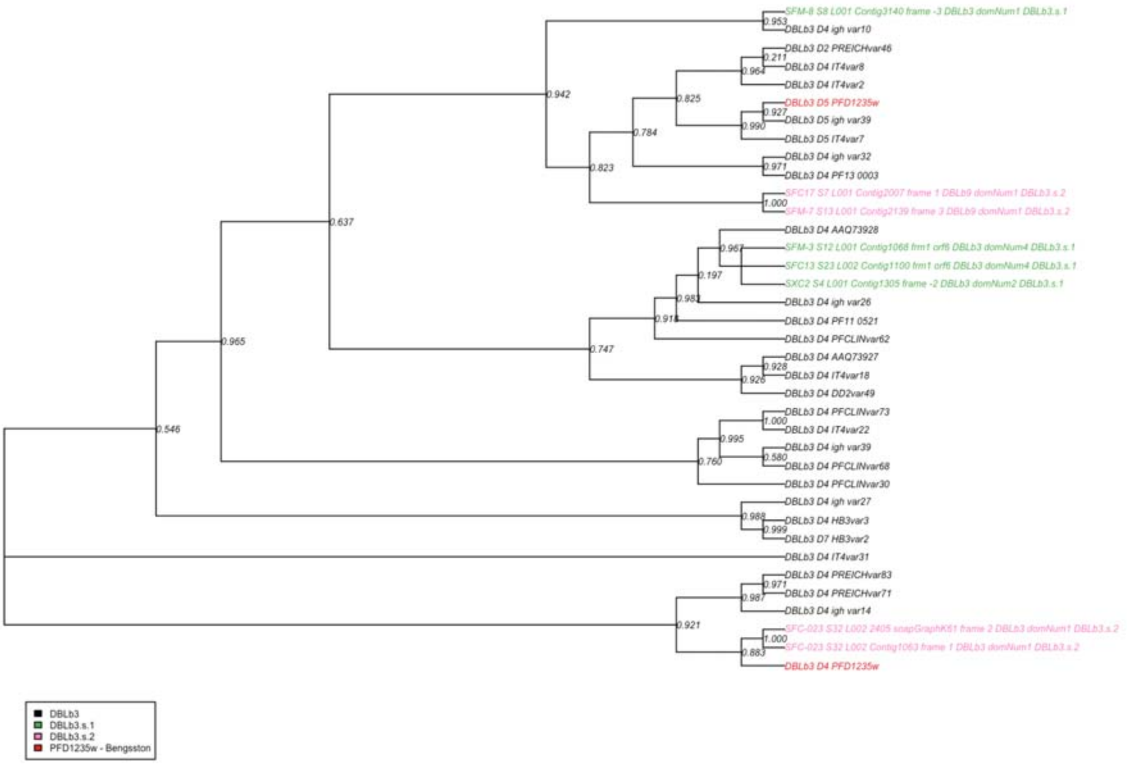

DC8 Comparison

Domain cassette 8, first decribed by Rask et al has been associated with severe forms of malaria. Here, the severe clusters identified in the domain analysis are clustered with those from domain cassette 8 of Rask et al. Specifically we look at DBLb12.s.1, DBLd1.s.1-4 and CIDRb1.s.1

DBLd1

The DBLd1 domains from DC8 containing proteins of Rask et al are failry well distributed amoungst the remaining DBLd1 domains of Rask et al. Notably, two DC8 DBLd1 domains cluster closely with severe domain clusters DBLd1.s.3 and DBLd1.s.4.

Severe clusters DBLd1.s.1 and DBLd1.s.2 also have DC8 DBLd1 domains nearby but are more closely clustered to a number of non DC8 DBLd1 domains.

```
tree <- read.tree("./data/compare_DC8/MA_combined_raskDBLd1_DBLd1s.fasta.phy")

labels <- "black"
labels[grepl("\\.s\\.1", tree$tip.label)] <- "#f781bf"
labels[grepl("\\.s\\.2", tree$tip.label)] <- "#984ea3"
labels[grepl("\\.s\\.3", tree$tip.label)] <- "#cab2d6"
labels[grepl("\\.s\\.4", tree$tip.label)] <- "#ff7f00"
labels[grepl("^DC8", tree$tip.label)] <- "#e41a1c"


plot.phylo(tree, type = "phylogram", use.edge.length = FALSE, show.node.label = TRUE,
    cex = 0.5, tip.color = labels, align.tip.label = TRUE)
legend(1, -1, legend = c("DBLd1", "DBLd1.s.1", "DBLd1.s.2", "DBLd1.s.3", "DBLd1.s.4",
    "DBLd1 from DC8"), fill = c("black", "#f781bf", "#984ea3", "#cab2d6", "#ff7f00",
    "#e41a1c"), xpd = NA, cex = 0.5)
```

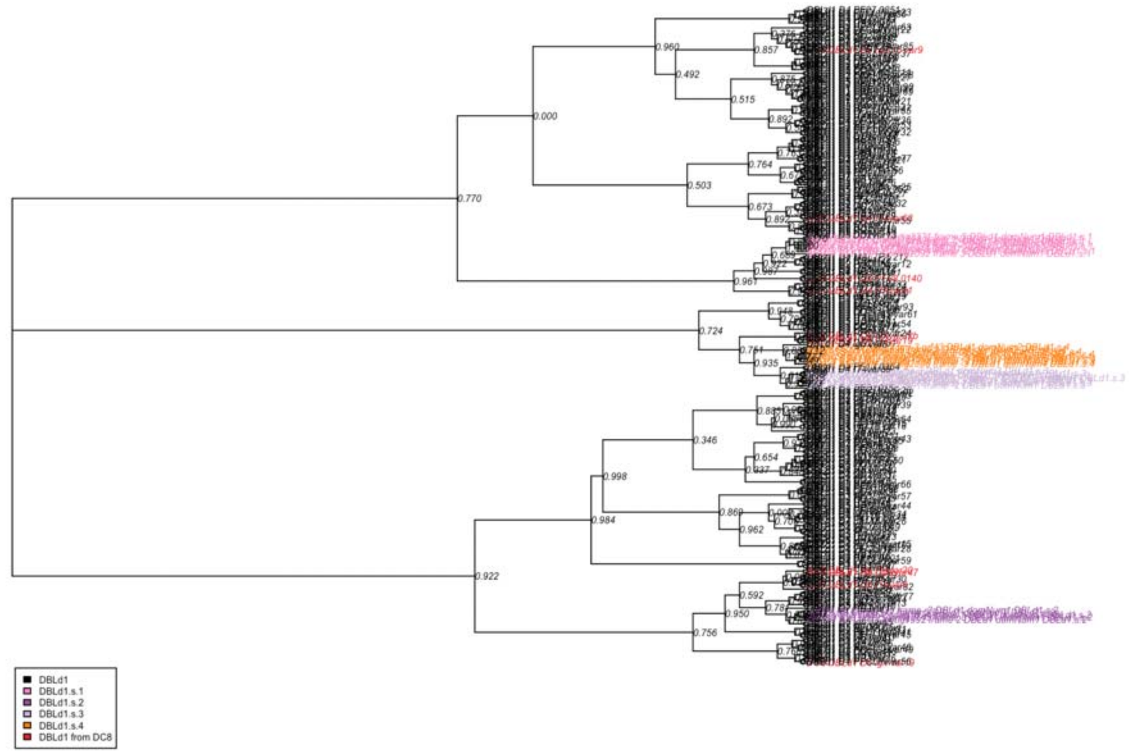

DBLb12

The tree below is not very informative due to the very high level of conservation found in the de novo assembled DBLb12 transcripts. However the DBLb12 transcripts from PFEMP1 domains of Rask et al split into two groups which are equally distant from each other as they are from the tightly clustered severe group. This suggests that the severe DBLb12 cluster could be grouped with those from DC8 as there is just as much variation between DC8 DBLb12 domains as there is between the severe group and DC8 DBLb12 domains.

```
tree <- read.tree("./data/compare_DC8/MA_combined_raskDBLb12_DBLb12s.fasta.phy")

labels <- "black"
labels[grepl("\\.s\\.1", tree$tip.label)] <- "#ff7f00"
labels[grepl("^DC8", tree$tip.label)] <- "#e41a1c"


plot.phylo(tree, type = "phylogram", use.edge.length = FALSE, show.node.label = TRUE,
    cex = 0.5, tip.color = labels, align.tip.label = TRUE)
legend(1, -1, legend = c("DBLb12", "DBLb12", "DBLb12 from DC8"), fill = c("black",
    "#ff7f00", "#e41a1c"), xpd = NA, cex = 0.5)
```

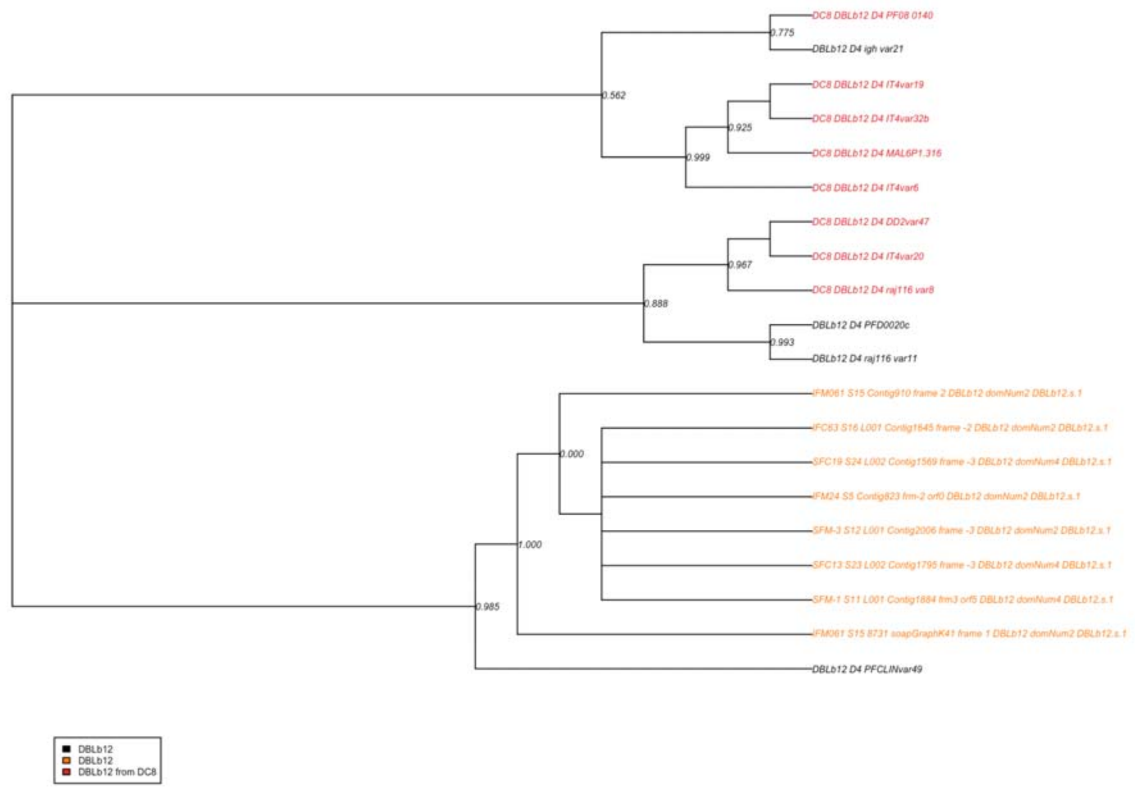

CIDRb1

The severe CIDRb1 domain clusters together with one DC8 CIDRb1 domain from Rask et al. The remaining CIDRb1 domains from proteins that conatin DC8 in Rask et al are distributed across the remianing CIDRb1 domains from Rask et al and don't form a disctinct group.

```
tree <- read.tree("./data/compare_DC8/MA_combined_raskCIDRb1_CIDRb1s.fasta.phy")

labels <- "black"
labels[grepl("\\.s\\.1", tree$tip.label)] <- "#ff7f00"
labels[grepl("^DC8", tree$tip.label)] <- "#e41a1c"


plot.phylo(tree, type = "phylogram", use.edge.length = FALSE, show.node.label = TRUE,
    cex = 0.5, tip.color = labels, align.tip.label = TRUE)
legend(1, -1, legend = c("CIDRb1", "CIDRb1.s.1", "CIDRb1 from DC8"), fill = c("black",
    "#ff7f00", "#e41a1c"), xpd = NA, cex = 0.5)
```

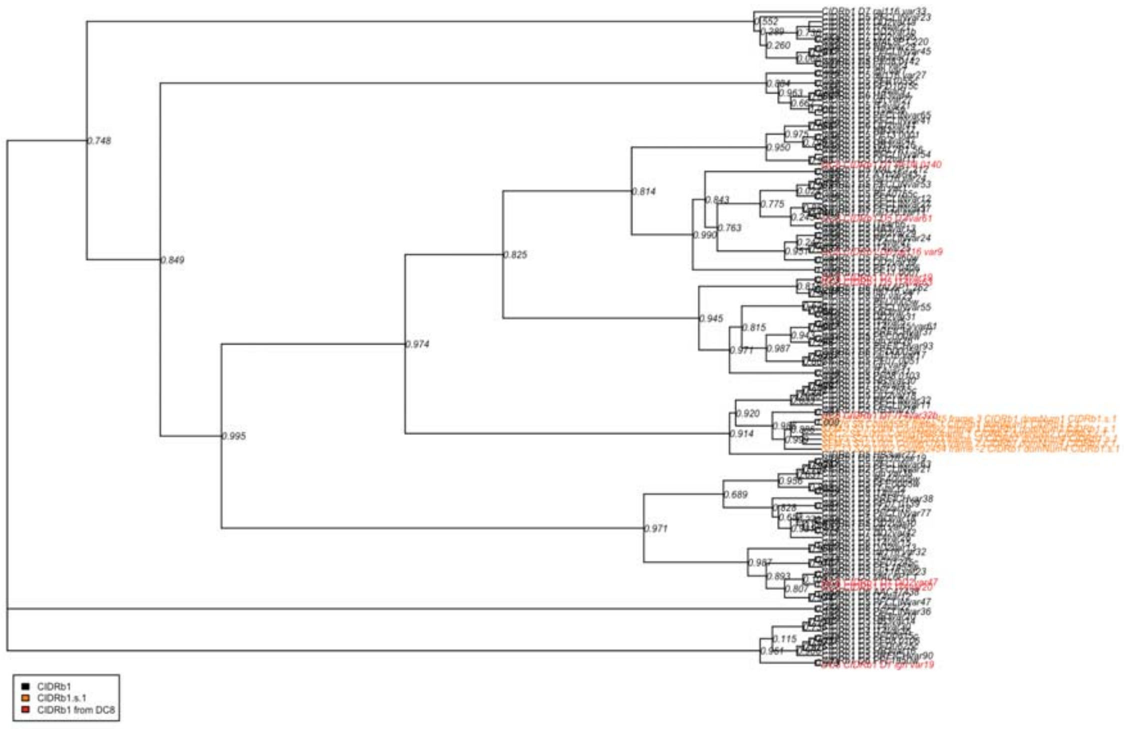

System Info

```
sessionInfo()
```

```
## R version 3.2.0 (2015-04-16)
## Platform: x86_64-apple-darwin15.4.0 (64-bit)
## Running under: OS X 10.11.4 (unknown)
##
## locale:
## [1] en_AU.UTF-8/en_AU.UTF-8/en_AU.UTF-8/C/en_AU.UTF-8/en_AU.UTF-8
##
## attached base packages:
## [1] stats     graphics  grDevices utils     datasets  methods   base
##
## other attached packages:
## [1] ape_4.1       ggplot2_2.2.1
##
## loaded via a namespace (and not attached):
##  [1] Rcpp_0.12.8      knitr_1.15.1     magrittr_1.5     munsell_0.4.3
##  [5] colorspace_1.3-2 lattice_0.20-31  stringr_1.1.0    plyr_1.8.4
##  [9] tools_3.2.0      parallel_3.2.0   grid_3.2.0       gtable_0.2.0
## [13] nlme_3.1-120     htmltools_0.3.5  yaml_2.1.14      lazyeval_0.2.0
## [17] rprojroot_1.1    digest_0.6.11    assertthat_0.1   tibble_1.2
## [21] formatR_1.4      evaluate_0.10    rmarkdown_1.3    stringi_1.1.2
## [25] scales_0.4.1     backports_1.0.4
```
