# Supplementary material for: The Plasmodium falciparum transcriptome in severe malaria reveals altered expression of genes involved in important processes including surface antigen–encoding var genes
Source: PLoS Biol. 2018 Mar 12;16(3):e2004328. doi: 10.1371/journal.pbio.2004328 (PMC5864071; doi:10.1371/journal.pbio.2004328)
Supplement: S1 Table — (PDF) [file pbio.2004328.s011.pdf]

S1 Table

Sample	Total	Human	Plasmodium falciparum	% Pf
IFC63	15834046	1269865	12803639	80.86
IFC66	12193434	2279696	8087337	66.33
IFD6	26316752	3250073	20485017	77.84
IFD8	34749712	6002524	22992058	66.16
IFM047	24478126	3732045	17790521	72.68
IFM049	27561002	8427559	4170351	15.13
IFM050	247859790	27710069	188483562	76.04
IFM054	18245068	2815650	13319325	73.00
IFM058	20168610	1071420	17180348	85.18
IFM060	6267320	932961	3771949	60.18
IFM061	17787684	829939	14984462	84.24
IFM12	27550076	768591	13834795	50.22
IFM14	21497208	1908349	4897916	22.78
IFM21	21571272	6268955	10061881	46.64
IFM23	24134898	11522063	7174755	29.73
IFM24	27184802	10754627	9337869	34.35
IFM26	24085582	395305	11180142	46.42
IFM27	21121462	1040771	6442191	30.50
IFM53	14394956	3530395	8468227	58.83
IFM56	11651938	6167390	2658755	22.82
IFM57	12404792	2259614	8225809	66.31
SFC023	12571658	1577309	8578807	68.24
SFC025	19194316	14230859	472323	2.46
SFC13	19549062	1359002	15599830	79.80
SFC14	16352520	71643	13819205	84.51
SFC15CM	14943012	183806	12365425	82.75
SFC16	12465608	31555	10829552	86.88
SFC17	11763562	33941	10326571	87.78
SFC18	12854344	893435	10305610	80.17
SFC19	15159600	99932	13349515	88.06
SFC21	12916510	957686	10190168	78.89
SFC22	26962436	642634	22655596	84.03
SFD1CM	17644040	13680384	18644	0.11
SFM10	21545814	134275	19028198	88.32
SFM1	12588172	366724	10544106	83.76
SFM3	14579124	490183	12164343	83.44
SFM5	6626030	2021312	2731652	41.23
SFM6	10573378	305179	9002853	85.15
SFM7	13914032	68139	12281107	88.26
SFM8	43474950	345290	38449165	88.44
SFM9	17054	770	3283	19.25
SFU2	11351208	155107	9739939	85.81
SFU3	10030170	137487	8218196	81.93
SXC2	11341534	45450	9899212	87.28

Raw read counts by sample for *human* and *Plasmodium falciparum*
