# Supplementary material for: The Plasmodium falciparum transcriptome in severe malaria reveals altered expression of genes involved in important processes including surface antigen–encoding var genes
Source: PLoS Biol. 2018 Mar 12;16(3):e2004328. doi: 10.1371/journal.pbio.2004328 (PMC5864071; doi:10.1371/journal.pbio.2004328)
Supplement: S2 Table — EIC and ECS are separate subcultures of the E8B clone of the ItG isolate; CS2 is a subclone of E8B. (PDF) [file pbio.2004328.s012.pdf]

S2 Table: comparison of approaches for *var* gene de novo assembly

| | Soap Cap3 | Soap K21 | Soap K51 | Oases Cap3 | Oases K21 | Oases K37 | Oases K61 | Trinity | Soap K21 no filter | Soap K51 no filter | Soap K51 human filter |
|---|---|---|---|---|---|---|---|---|---|---|---|
| **Correctly assembled ECS** | Y | N | Y | Y | Y | Y | Y | Y | N | N | N |
| **Correctly assembled CS2** | Y | N | N | Y | N | Y | Y | N | N | N | N |
| **Redundancy in EIC** | 1.048 | 1.003 | 1.005 | 1.389 | 3.010 | 2.307 | 1.601 | 1.464 | 1.004 | 1.004 | 1.004 |
| **# of genes found by both qPCR and assembly in EIC** | 40 | 23 | 37 | 40 | 28 | 36 | 35 | 30 | 23 | 39 | 40 |
| **Pearson Correlation between qPCR and assembly RPKM** | 0.877 | 0.961 | 0.928 | 0.559 | 0.925 | 0.595 | 0.460 | 0.765 | 0.886 | 0.786 | 0.804 |
