# Supplementary material for: The Plasmodium falciparum transcriptome in severe malaria reveals altered expression of genes involved in important processes including surface antigen–encoding var genes
Source: PLoS Biol. 2018 Mar 12;16(3):e2004328. doi: 10.1371/journal.pbio.2004328 (PMC5864071; doi:10.1371/journal.pbio.2004328)
Supplement: S3 Table — (PDF) [file pbio.2004328.s013.pdf]

S3 Table: *de novo* assembled *var* transcript statistics by sample.

	Mean Length	Major N50	Max Length	Number of Transcripts >500nt
IFC63	2123.415385	3747	7029	65
IFC66	1671.536036	2414	9273	222
IFD6	1276.065789	1719	8539	76
IFD8	1249.730769	1620	8516	78
IFM-049	1506.085938	2008	9662	128
IFM047	1565.803681	2001	9445	163
IFM050	1612.517928	2126	7498	251
IFM054	1729.94	2089	5527	100
IFM058	1379.366667	1712	8277	120
IFM060	1102.615385	1180	3131	13
IFM061	1896.72381	3303	10262	105
IFM12	1701.59434	2536	10527	106
IFM14	1591.5	2630	5725	20
IFM21	1149.219512	1311	5399	123
IFM23	2102.081967	4645	10559	61
IFM24	2133.462687	3694	10188	67
IFM26	1439.72619	1705	8491	84
IFM27	1553.52	2433	6696	125
IFM53	1626.158537	2704	10385	82
IFM56	1794.9	3258	6946	20
IFM57	1825.924812	2558	10083	133
SFC-023	1918.052632	4105	10021	19
SFC-025	890.6923077	848	1708	13
SFC-18	1740.881579	2711	6724	76
SFC-21	1276.698529	1654	6586	136
SFC13	1500.98125	1859	9174	160
SFC14	1682.010204	2044	9506	98
SFC15-CM	1678.641509	2630	9372	106
SFC16	2183.109375	4042	10193	64
SFC17	1761.725275	2685	8099	182
SFC19	2135.554054	4306	9404	74
SFC22	1621.348624	2264	10225	218
SFM-10	1669.978261	2754	7596	138
SFM-1	1717.333333	2575	7708	117
SFM-3	1845.181034	2617	10074	116
SFM-5	795.725	749	1893	40
SFM-6	1313.197917	1654	4558	96
SFM-7	1800.178571	2441	8467	112
SFM-8	1787.385475	2675	8650	179
SFU-3	1384.282258	1752	6159	124
SFU2	1760.556604	2883	8594	106
SXC2	1781.240602	2566	9964	133
Total Individual Assemblies	1638.35581	2336	10559	4449
Combined Assembly	819.5322581	802	6502	3472
