# Supplementary material for: The Plasmodium falciparum transcriptome in severe malaria reveals altered expression of genes involved in important processes including surface antigen–encoding var genes
Source: PLoS Biol. 2018 Mar 12;16(3):e2004328. doi: 10.1371/journal.pbio.2004328 (PMC5864071; doi:10.1371/journal.pbio.2004328)
Supplement: S4 Table — FDR, false discovery rate; Prp, proportion. (DOCX) [file pbio.2004328.s014.docx]

S4 Table De-regulation in severe malaria of *P. falciparum* gene-sets previously reported to be de-regulated *in vivo*

| Gene set^a^ | N Genes | Prop Down | Prop Up | Direction | P Value | FDR | PValue.  Mixed | FDR.  Mixed |
| --- | --- | --- | --- | --- | --- | --- | --- | --- |
| tricarboxylic | 11 | 0.1818 | 0.3636 | Up | 0.0177 | 0.0265 | 0.0012 | 0.0069 |
| glycolysis | 19 | 0.5789 | 0.0526 | Down | 0.017 | 0.0265 | 0.0157 | 0.0188 |

a Up *in vitro* (genes that were up in cluster 2 *in vitro* ring stage like profile from Daily *et al*  2007); Up starvation (genes that were up in cluster 1 starvation like profile from Daily *et al* 2007): glycolysis (glycolysis gene-set from Daily *et al*  2007); tricarboxylic (TCA cycle gene-set from Daily *et al*  2007).
